# The Yin and Yang of Yeast Transcription: Elements of a Global Feedback System between Metabolism and Chromatin

**DOI:** 10.1371/journal.pone.0037906

**Published:** 2012-06-07

**Authors:** Rainer Machné, Douglas B. Murray

**Affiliations:** 1 Institute for Theoretical Chemistry, University of Vienna, Vienna, Austria; 2 Institute for Advanced Biosciences, Keio University, Tsuruoka, Japan; Université Joseph Fourier, France

## Abstract

When grown in continuous culture, budding yeast cells tend to synchronize their respiratory activity to form a stable oscillation that percolates throughout cellular physiology and involves the majority of the protein-coding transcriptome. Oscillations in batch culture and at single cell level support the idea that these dynamics constitute a general growth principle. The precise molecular mechanisms and biological functions of the oscillation remain elusive. Fourier analysis of transcriptome time series datasets from two different oscillation periods (0.7 h and 5 h) reveals seven distinct co-expression clusters common to both systems (34% of all yeast ORF), which consolidate into two superclusters when correlated with a compilation of 1,327 unrelated transcriptome datasets. These superclusters encode for cell growth and anabolism during the phase of high, and mitochondrial growth, catabolism and stress response during the phase of low oxygen uptake. The promoters of each cluster are characterized by different nucleotide contents, promoter nucleosome configurations, and dependence on ATP-dependent nucleosome remodeling complexes. We show that the ATP:ADP ratio oscillates, compatible with alternating metabolic activity of the two superclusters and differential feedback on their transcription *via* activating (RSC) and repressive (Isw2) types of promoter structure remodeling. We propose a novel feedback mechanism, where the energetic state of the cell, reflected in the ATP:ADP ratio, gates the transcription of large, but functionally coherent groups of genes *via* differential effects of ATP-dependent nucleosome remodeling machineries. Besides providing a mechanistic hypothesis for the delayed negative feedback that results in the oscillatory phenotype, this mechanism may underpin the continuous adaptation of growth to environmental conditions.

## Introduction

Stable oscillatory dynamics in continuously grown budding yeast were first observed almost 60 years ago. The authors concluded that “the phenomenon appears to arise from the inherent feedback in the system coupled with a metabolic lag” [1], [2], in line with the current paradigm in systems biology where a “negative feedback with delay” [3] is thought to underlie biochemical oscillators [4], [5]. However, the nature of this putative feedback remains elusive for the case of yeast respiratory oscillations, partially due to the extent to which they percolate throughout cellular physiology: many measured metabolites oscillate, notably central carbon intermediates [6], amino acids [7], [8] nucleotide precursors [8] and a majority of the measured protein-coding transcriptome [9]–[12]. The period is strain- and condition-dependent and ranges between half an hour [13], [14] and several hours [1], [15], [16]. Each cycle alternates between a phase of high oxygen uptake (oxidative phase) and a phase of low oxygen uptake (reductive phase) [17]. Resistance to diverse cellular stress conditions varies over the cycle [18] and oxidative damage, measured by lipid peroxidation, was shown to be at maximum during the oxidative phase [19]. Moreover, S-phase cells are enriched during a temporal window of each cycle [9], [10], [15], [20] leading to the hypothesis that the major function of the oscillation is the partitioning of DNA replication from reactive oxygen species produced during the oxidative phase [9], [21]. However, DNA replication can occur in the oxidative phase under low glucose conditions [20] and the oscillation can persist in cultures close to a non-growing state [12]. Thus, it remains largely unclear whether the oscillation serves a biological function or is a condition-specific artefact of the many non-linear feedback systems that regulate cellular growth [16]. However, evidence of single cell oscillations [22], [23] and coherence of oscillatory processes over several time-scales [24] indicate that this cycling behavior may well constitute a general principle of growth.

A range of mechanistic models have been proposed, but none can accomodate the full range of experimental observations [25]. Previously, we defined a biosynthetic program, where cytoplasmic ribosomal transcripts were upregulated at the beginning of the oxidative phase, followed by sequential upregulation of many transcripts involved in biosynthetic pathways. The end of this program was characterized by the upregulation of mitochondrial ribosomal and stress response transcripts during the reductive phase [9]. Further analysis based on the yeast transcription factor network [8] could only give a partial picture of the regulatory events underlying the oscillation. These analyses were based on a system that oscillates with a period of 0.7 h. A subsequent transcriptome experiment from a culture that oscillated at a period of 5 h (but at comparable culture doubling times of 7–8.5 h) revealed a similar picture [10], but the exact relation between the systems remains unclear [26]–[28]. In this work, we directly compare these two systems. Recently, a strong correlation of the oscillatory transcriptome to the “environmental stress response” (ESR), where hundreds of genes are either upregulated or downregulated upon infliction of a variety of cellular stress conditions [29], [30], had been noted [31]. It was hypothesized that even in steady-state cultures single cells may still undergo an oscillatory growth program [23], and that the stress response is in fact just a culture average signal resulting from a shift in the relative lengths of the phases of high and low oxygen consumption in individually oscillating but non-synchronized cells [20]. This hypothesis has far-reaching consequences for the interpretation of all previous experimental data taken from steady-state cultures. A complementary interpretation of the stress response was based on a refined functional analysis and postulated that it serves to “balance energetic supply/demand and coordinate growth with the cell cycle” [32]. Both, the stress response and respiratory oscillations, involve a fast genome-wide remodeling of transcription, implying a more general mechanism of gene regulation, beyond the activity of specific transcription factors with only small sets of target genes. Unlike so-called house-keeping genes, the genes that are activated by stress were found to be enriched with TATA Boxes [33], depend on the SAGA complex (Spt-Ada-Gcn5-Acetyl transferase) for transcriptional initiation [34] and have a more “bendable” promoter DNA that is thought to favor nucleosome binding [35]. Recent genome-wide nucleosome occupancy data allowed to distinguish four different types of promoter nucleosome configuration [36], and such differential nucleosome occupancy and positioning are thought to arise in part from DNA sequence motifs or more general sequence properties [37]–[39] and in part from “nucleosome remodeling”, the enzymatic shifting or ejection of nucleosomes away from eneregetically favorable sites on DNA [40]–[43]. Recently, *in vivo* -like promoter nucleosome configurations (“positioning, spacing and occupancy levels”) were observed *in vitro* when Adenosine-5′-triphosphate (ATP) was added to a mixture of whole-cell extract and nucleosomes reconstituted on genomic DNA of budding yeast. This suggests a major role of ATP-dependent remodeling in the establishment and maintenance of different types of promoter nucleosome configurations [43]. ATP is one of the major intracellular “currency metabolites” that channels chemical energy from nutrient-catabolic processes into a multitude of cellular growth and maintenance functions. Such direct links between central energy metabolism and genome structure, impacting on gene expression, have recently been implicated also in mammalian regulatory systems such as the circadian clock [44] and cancer cell growth [45], [46], and are also suspected to play a major role in eubacterial growth regulation *via* negative supercoiling and ATP-dependent gyrase [47]–[51], which by itself was observed to underlie the genome-wide circadian remodeling of gene expression in cyanobacteria [52], [53].

Thus, a vague line of interrelations exists in literature, from stress-regulation *via* sequence properties of promoters to their differential nucleosome configurations, and from central energy metabolism to feedback on DNA structure. We reasoned that the phenomenon of respiratory oscillations could clarify and consolidate these various detail observations. We developed a novel clustering strategy, based on the discrete Fourier transform (DFT) of raw transcriptome time series taken from the two systems oscillating at periods of 0.7 h [11] and 5 h [10]. This allowed to define a temporal sequence of co-expression cohorts common to both systems and to characterize the differences. This consensus clustering then served to systematically interrogate a large set of published experimental data, and interpret the underlying biological concepts in the context of oscillatory growth dynamics. The respiratory oscillation transcriptomes untangle the enigmatic stress response and integrates it with the recent observations of general gene and promoter structures into a temporally and functionally coherent growth program. Taken together, a surprisingly simple perspective on global feedback mechanisms of eukaryotic growth emerges, suggesting that the energetic state of the cell gates transcription *via* co-factor dependent chromatin modifications to express either cell growth and anabolic, or mitochondrial growth and catabolic gene groups.

## Results

### Co-expression Cohorts Common to Both Systems

**Figure 1:**
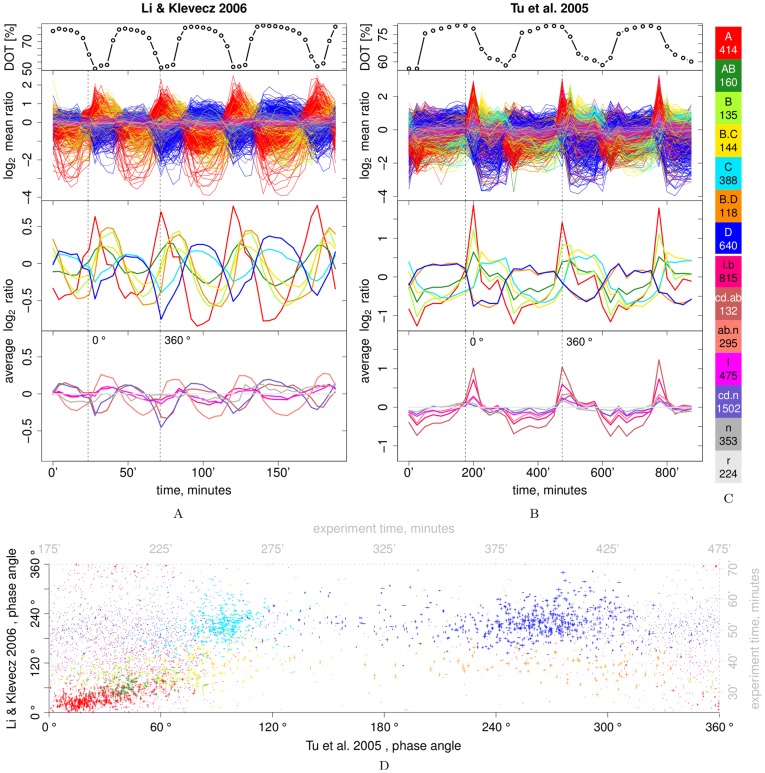
Clustered transcript time course profiles. 0 and 0: overlaid time courses of summarized microarray fluorescence for each yeast gene, as the 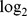 of the mean-ratio (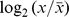), for the 0.7 h [11] and 5 h [10] period datasets, respectively. The bottom two panels show cluster averages for consensus and background clusters. The top panel shows the time courses of the dissolved O_2_ trace (DOT) in the culture medium in percent of the saturated concentration. Cluster colors and sizes (number of genes in each cluster) are given in the legend in Figure 1C. For clarity of visualization the time course data was normalized to a reference set that was selected for significant lack of oscillation (see Text S1 for fundamental problems with normalization of these datasets). Individual time courses for each cluster are plotted in Figure S2. 1D: phase-phase plot comparing the phase-angles 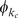 of all transcripts in the two experiments. The phase angles were shifted such that cluster A phase angles are just above 0° in both datasets. Mapping back from frequency- to time-domain, we can locate the shifted phase angles of one cycle (0° and 360°) in the time series plot (vertical lines in Figures 1A and 1B), and use the same mapping in the top and right axes (in gray) of the phase-phase plot. The x- and y-extensions of each point scale with the transcript’s scaled amplitude 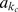 in the respective dataset, where the non-consensus clusters (lower case letters) have a smaller initial size. Dataset S1 provides raw summarized microarray intensities, and the clustering of all analyzed yeast genes.

Here we compare two previously published microarray-based transcriptome time series from cultures oscillating with periods of 0.7 h [11] (Figure 1A) or 5 h [10] (Figure 1B). The two experiments were performed with different yeast strains (*Saccharomyces cerevisiae* IFO 0233 or CEN.PK122) and different media composition (20 or 10 g L^−1^ glucose and 13 or 6.5 mmol L^−1^ H_2_SO_4_; see Table S1). Phenelzine was added at the end of the first cycle of the 0.7 h system, inducing a period increase from 0.7 h to 1.2 h during the experiment [11]. The DFT of microarray time series has previously proven useful in identifying periodic changes in mRNA abundance [54], [55]. Here it allows for a direct comparison of the two transcriptome time series by a scatter-plot of the phase angles at the respective phenotypic oscillation periods (indicated by the dissolved O_2_ concentration in the culture medium). This phase-phase plot reveals at least three density peaks (Figure 1D and Text S1). To further characterize these co-expression cohorts, an apt model-based clustering algorithm flowClust [56] was used to cluster selected and scaled DFT components of all transcript time series. This clustering strategy is very similar to a previously used approach [57], [58] and naturally allows to cluster by the pattern of change of fluorescence levels, *i.e.*, account for the time series nature of the datasets. Amplitude scaling and the tailed distribution model of the clustering algorithm are different from the previous work and serve to further de-emphasize the only semiquantitative amplitude information in favor of overall change patterns. Simultaneously, this strategy allows to avoid a problematic data normalization step, since the array-to-array noise can be expected in high-frequency components of the DFT. The Methods section gives all technical details of data processing and clustering, while in Text S1 we provide detailed accounts of normalization problems, selection of DFT components and the choice of the clustering algorithm.

The resulting clusters were sorted by the density peaks of their phase angles for each dataset. The significance of overlaps between the two individual clusterings was established by cumulative hypergeometric distribution tests (Figure S1) and guided the definition of a temporal sequence of five co-expression clusters common to both systems: A
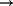
AB
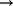
B
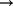
C/D in the 0.7 h period and A/AB/B
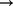
C
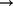
D in the 5 h period oscillation (Figures 1A & 1B). Genes in clusters B.C and B.D are differentially expressed between the two systems, *i*.e., similar to clusters A to B in the 0.7 h cycle and similar to cluster C and D in the 5 h cycle. Each of these 7 consensus clusters comprises 118 to 640 genes (Figure 1C), totaling 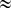
34% of the yeast genome (1,999 of 5,795 yeast protein-coding genes in our reference genome release). The remaining transcripts could be assigned to low-amplitude clusters l.b (815 genes, similar to cluster B) and cd.n (1,502, similar to cluster C/D), to noisy and/or non-consensus time series (cd.ab, ab.n, l, n) or were not present on the microarray (r). Transcript abundance of cluster A genes peaks when respiratory activity is maximal (0.7 h) or accelerating (5 h). The more frequently sampled dataset from the 0.7 h period (sample resolution: 4 min) reveals a rapid temporal sequence of peaks A
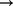
AB
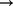
B (Figure 1A). The transition between the oxidative and reductive phase coincides with maxima of cluster B/B.C/B.D (0.7 h) or C/B.C (5 h) transcript abundance. While cluster C time series are in phase with cluster D in the 0.7 h cycle, their phase angle density peaks are shifted by 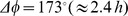 in the 5 h cycle (Table S2, Figure 1B). The end of the reductive phase corresponds to a decrease in abundance of cluster D transcripts and then the cycle resets. In summary, the DFT-based clustering analysis shows that there is a defined series of events that occurs in each cycle and common to both the 0.7 h and the 5 h systems.

### A Functionally Coherent Program: Anabolism *vs.* Catabolism

We next analyze gene ontology (GO) terms and “subsystem” annotations in a genome-scale metabolic network model [59] (Table 1, Tables S3 & S4) to identify the cellular processes that are temporally regulated, and to expand and refine the pictures drawn previously [8]–[10]. Large groups of cellular growth machinery (A & AB: ribosomes of the cytosol, C: ribosomes of the mitochondria) and architecture (A: nucleolus, B.C & C: mitochondria, D: peroxisomes, vacuoles) are associated with enrichment in certain metabolic pathways, which indicate apt shifts of metabolic flux towards the specific requirements of the respective oscillation phase. Purine (A) and amino acid synthesis (B) genes are expressed in time to “feed” the protein translation program of clusters A and AB. Transcripts encoding for sulfate uptake and methionine synthesis are associated with cluster A and thus precede the rest of the amino acid synthetic program. Cluster B.C is enriched with genes encoding for the DNA replication machinery (S-phase), apparently at the start of a cell division program that is followed by M-phase functions enriched in clusters C (spindle and kinetochore) and D (cytokinesis). Clusters AB, B.C and B.D together comprise genes encoding for the amphibolic core carbon backbone (glycolysis/gluconeogenesis, TCA/glyoxylate bypass). Mitochondrial regeneration or growth, mediated by ribosomes encoded in cluster C, and the catabolic genes in cluster D, would then switch flux around this backbone towards oxidation and energy generation for the next oxidative phase. Cluster D further is enriched in genes involved in cell redox homeostasis and response to stress, which may prepare for the oxidative stress during the next oxidative phase. In line with their time courses’ similarity to the main consensus clusters, cluster l.b is enriched with genes encoding for general transcription, mRNA processing, chromatin remodelers and cell-cycle functionality required for both G_1_/S and G_2_/M transitions, and cluster cd.n with protein-degradation and autophagy. Taken together, a cell growth and anabolic supercluster (A, AB & B) is expressed in the oxidative (energy-mobilizing) phase of the cycle, while the reductive phase supercluster (C & D) encodes for mitochondrial growth and catabolism, *i*.e. mediates energy mobilization during the subsequent oxidative phase.

**Table 1:** Significantly enriched GO terms of consensus clusters.

cluster	Cell Structure & Growth	Metabolism	Cell Division & Life Cycle
A (414)	nucleolus (137/175), *PolI* (14/14), *PolIII* (14/17),ribosome biogenesis (171/199) & export(5/10, *LSU*: 6/11)	sulfate assimilation (7/10), methionine*BSP* (4/6), purine nucleotide*BSP* (7/11)	
AB (160)	cytoplasmic *RP* (*LSU*:55/87, *SSU*: 43/62),translation (102/270)	glycolysis (4/16), gluconeogenesis(4/15)	
B (135)	protein *de novo* (2/3) & re- (3/9) folding,actin cap (4/12), plasma membrane(13/215)	amino acid* *BSP* (25/43), purine base*BSP* (3/5), amino acid transport (3/14),allantoin *CP* (5/7), nitrogenutilization (3/9)	
B.C (144)	mitochondrion (57/988)	glutamate *BSP* (6/13), citrate *MP* (3/4),tricarboxylic acid cycle (8/15), glyoxylatecycle (2/4), *RCC* II (2/4), III (4/10) & IV(3/12), ATP synthesis coupled protontransport (7/20)	DNA replication (6/24), replication fork (7/14), lagging strand elongation (7/16), DNA synthesis during DNA repair (3/3), mitotic sister chromatid cohesion (9/22)
C (388)	mitochondrion (225/988), mito. *RP* (*LSU*: 42/44,*SSU*: 31/33), translation (88/270), structuralconstituent of the cytoskeleton (11/51)	aerobic respiration (23/69), mito. proton-transporting ATP synthase complexassembly (3/3), *RCC* IV assembly (6/9)	septin complex (3/4), spindle (4/10) & kinetochore microtubule (3/6)
B.D (118)	mito. matrix (6/61), peroxisomalmatrix (3/12)	arginine *BSP* (6/10), proline *CP* (2/3),ammonium transport (2/6), siderophoretransport (2/3), heme binding (2/3),carnitine *MP* (3/3), propionate *MP* (3/5),gluconeogenesis (3/15)	fungal-type cell wall (7/87)
D (640)	fungal-type vacuole (26/99), peroxisome (11/27),cell redox homeostasis (6/11), response tostress (29/68), protein kinase activity (14/48),unknown process (188/1313) & function(266/2049)	vacuolar protein *CP* (9/12), trehalose*CP* (3/3), D-xylose *CP* (3/4), arabinose*CP* (3/4), neg. reg. of gluconeogenesis(5/9), ethanol *MP* (3/4), carbohydrate*MP* (6/12), glutathione *MP* (5/8), fatty acid 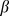 -oxidation (6/9), glycogen *BSP* (5/9),trehalose *BSP* (5/7)	cytokinesis, completion of separation (5/11), fungal-type cell wall (18/87)

Cellular functions and metabolic activities are indicated by gene ontology (GO) categories that are significantly enriched in clusters (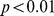 in cumulative hypergeometric distribution tests). GO terms were taken from the SGD genome annotation file and only direct annotations were used, *i*.e., annotations were not propagated to their parent terms in the GO structure. Redundant terms were manually filtered and categorized into the three columns of the table. Only consensus clusters are shown and the rest of clusters are given in Table S3. The full data, all GO terms and p-values for all clusters, are provided as Dataset S2. The numbers in brackets show the number of genes in the cluster and the total number of genes with the respective annotation. Abbreviations: mito., mitochondrial; neg.reg., negative regulation; *PolI* and *PolII*, DNA-directed RNA polymerase complex I and III, respectively; *RP*, ribosomal protein; *LSU* and *SSU*, large and small ribosomal subunit, respectively; mito., mitochondrial; *RCC*, respiratory chain complex; *BSP*, biosynthetic process; *CP*, catabolic process; *MP*, metabolic process; *ER*, endoplasmatic reticulum. (*) reported is the sum of all significantly enriched amino acid biosynthetic pathways, *i*.e., lysine (*via* aminoadipic acid, 6/8), branched chain (5/7), aromatic (3/5), leucine (3/5), histidine (4/14), asparagine(glutamate-hydrolyzing, 2/2) and arginine (metabolic process, 2/2).

### Growth and Stress *vs.* Cellular Energetics

**Figure 2:**
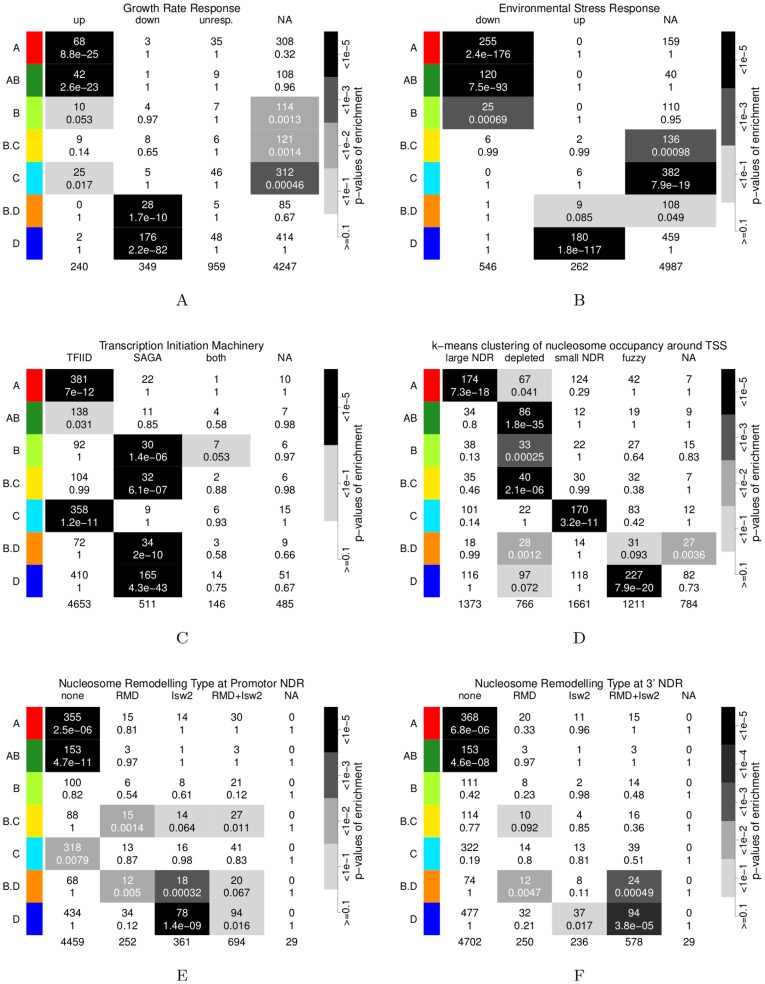
Overlap of the consensus clusters with other gene clusterings. Clusters were tested for enrichment in other gene categorizations by cumulative hypergeometric distribution tests. The text in the fields gives the number of genes in the respective overlap (top line) and the p-values (bottom line). The p-values are further indicated by gray-scale (see legend to the right of each panel). The bottom row gives the total number of genes in each tested category. Figures S4 & S5 give results for all 14 clusters and Dataset S7 provides the original gene classifications. “NA” indicates that no classification was available for these genes in the respective dataset. 2A: genes whose expression positively (“up”) or negatively (“down”) correlates with, or does not respond (“unresp.”) to growth rates in nutrient-limited conditions, data from [31]. 2B: genes which are upregulated (“up”) or downregulated (“down”) in response to a variety of stress conditions, data from [29] *via* supplementary material of [31]. 2C: dependence on transcription initiation complexes “TFIID”, “SAGA” or “both”, from [34]. 2D: genes with fuzzy nucleosome positioning (“fuzzy”), nucleosome-depleted promoters (“depleted”), a large and pronounced NDR (“large NDR”) or a small but pronounced NDR (“small NDR”), from [36]. 2E: genes with no Isw2(K215R) binding but remodeling at promoter NDR (“RMD”), with Isw2(K215R) binding but no remodeling (“Isw2”), with Isw2(K215R) binding and remodeling (“RMD+Isw2”) or neither binding nor remodeling (“none”), data from [40]. 2F: as Figure 2E but for the NDR at 3′ ends of genes.

**Figure 3:**
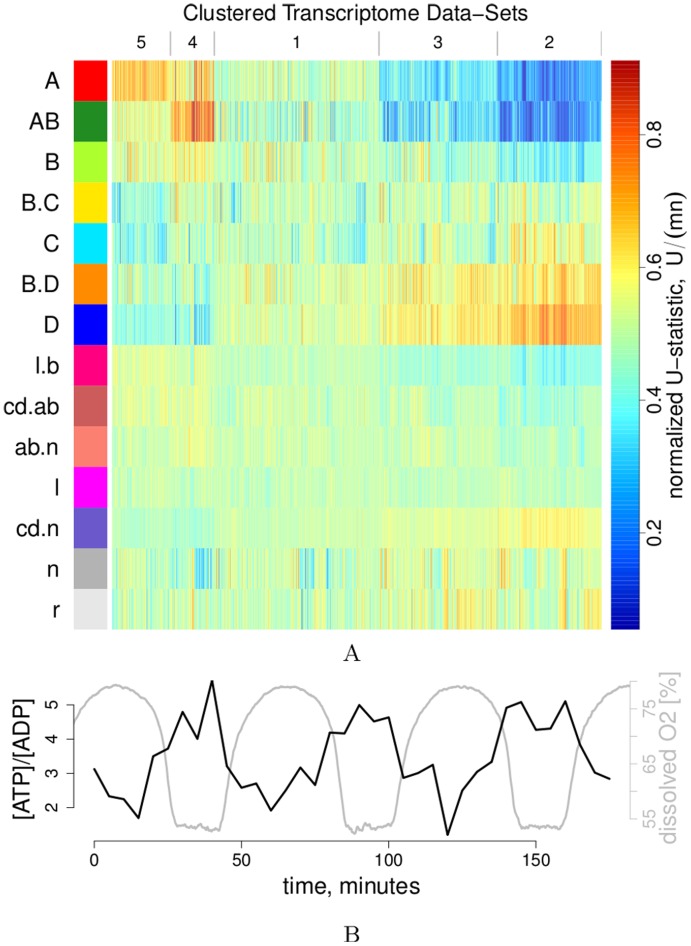
Cluster transcriptome meta-analysis & ATP:ADP ratio. 3A: Relative RNA expression profiles of redox clusters (rows) in a collection 1,327 microarray hybridization datasets [60] (columns). The normalized rank sum 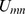 indicates a bias of the cluster genes towards upregulation (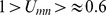, red to yellow) or downregulation (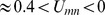, cyan to blue) in the respective experiment. Experiments were sorted into 5 clusters (column numbers) by the SOTA algorithm [104] and plotted in decreasing order (from left to right) of the means of cluster A’s 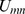 values. The Dataset S3 gives SOTA assignments, 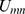 values and p-values from two-sided Mann-Whitney-Wilcoxon tests. 3B: The ATP:ADP ratio was measured enzymatically every 5 minutes over three cycles of a respiratory oscillation and culture system that corresponds to the 0.7 h period dataset (available as Dataset S8).

The functional profiles of the clusters, especially of the two antiphase clusters A and D, are reminiscent of the environmental stress response (ESR) to various cellular stress conditions [29], [30], [32]. This relation had been previously noted [20], [31] and is reflected in sequence motif and binding site enrichments in the promoters of cluster genes (Table S5, Figure S3 and Datasets S5 & S6), *e*.g., the RRPE and PAC motifs in cluster A, and STRE motif and Msn2/Msn4 binding sites in cluster D [32]. We find highly significant overlaps of clusters A & AB with gene groups [29], [31] downregulated in response to stress and positively correlating with growth rate and of clusters D & B.D with those upregulated upon stress and negatively correlating with growth rate (Figures 2A, 2B & S7C). A statistical analysis of the cluster distributions of transcript levels in a previously published collection of 1,327 individiual transcriptome microarray hybridizations [60] confirms a general anti-correlation in expression between clusters A, AB & B, and clusters D & B.D (Figure 3A). Cluster C expression is more diverse but overall correlates positively with cluster D, *i*.e. Spearman’s correlation of the normalized rank sums in Figure 3A is 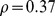 (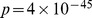). The regulatory antagonism, *i*.e., when one gene group is downregulated the other is upregulated, is most apparent between clusters A and D (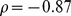, 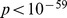) and is further reflected in strong biases in various measures of expression kinetics, such as transcriptional frequency, protein level and noise (Figure S7).

The ESR has been proposed to balance cellular energetics by downregulating costly translation and upregulating catabolic (energy-mobilizing) programs [32]. Free ATP has been shown to oscillate [13]. Since cells are growing and total nucleotide levels may vary, the ATP:ADP ratio provides a better estimate of the energetic state, and we find that it oscillates between 1.2–2 in the middle of reductive phase, and 5–5.7 in the oxidative phase (Figure 3B). Thus, transcript abundance of cluster A genes coincides with high and of cluster D genes with low energy states, in agreement with the suspected role of energy limitation in the ESR [32].

### A “Dual Dichotomy”: Stress-regulated or House-keeping *vs.* TATA or TATA-less Genes

Besides a variety of specific transcription factors, general DNA-structural properties or transcription initiation machineries have been implicated in differential regulation of large gene classes. In particular, genes that do not contain a TATA Box code for “house-keeping” genes [33], have a stiff promoter [35] with a pronounced nucleosome-depleted region (NDR) [36]; their expression depends on the TFIID-type transcription initiation machinery [34] and protein levels are less noisy [61]. These genes are thought to differ in all above features from genes classified as “stress-regulated”. The rRNA-processing and mitochondrial ribosome clusters A and C consist primarily of TFIID-controlled genes (Figure 2C), while clusters B, B.C, B.D & D are all significantly enriched in the smaller class of genes under control of the SAGA transcription initiation complex. Consistent with this, only 23–29% of cluster A, AB and C genes, but 41–52% of genes from clusters B, B.C, B.D and D harbor a consensus TATA Box [33] within 350 nucleotides upstream of their start codons (row TATA.350 in Figure S3A). Clusters A & C further share a bias towards low RNA half-lives (Figure S8A), possibly indicating induced mRNA degradation. The proteins Puf4p and Puf3p promote mRNA degradation and their binding motifs [62] are enriched in the 3′UTR of clusters A & C, respectively (PUF4p.3p and PUF3.3p in Figure S3A). The latter enrichment had already been observed for the 5 h period system [63]. Clusters A & C, but also the low amplitude background clusters, differ by a low chromatin regulation score (CRE, Figure S8B), defined by the expression response to a range of perturbations of chromatin regulation machineries [64]. All other main clusters, especially clusters B.D & D, are characterized by high CRE scores (all p-values <10^−4^). In summary, our analyses show that the broad classification of genes into cell growth and energy-mobilizing superclusters, reflected in a plethora of independent transcriptome and transcription kinetics datasets (Figures 3A, S7 & S8), is orthogonal to previously observed promoter-structural categories. Temporally, clusters A and C, encoding for cytoplasmic and mitochondrial ribosome biogenesis, lead the anabolic and catabolic superclusters, respectively. These are exclusively TFIID-regulated, deprived of TATA Boxes and are targeted by Puf proteins. Each supercluster then develops to express metabolic genes, whose promoters are enriched in TATA Boxes and SAGA-regulation, *i*.e., clusters B and D.

### Differential Chromatin Structure: Broad Gene Classes

**Figure 4:**
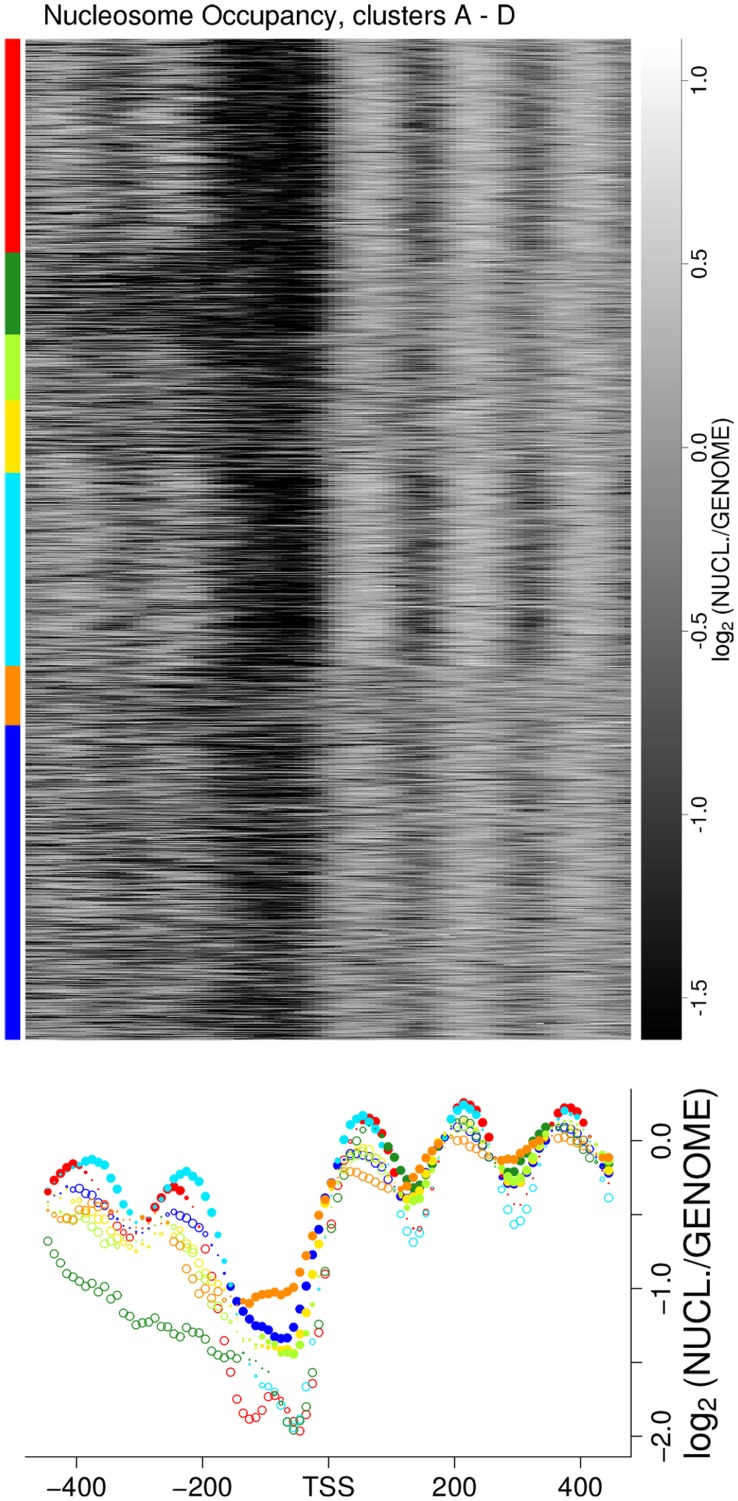
Nucleosome Occupancy: Heatmap and SDP Construction. A heatmap of nucleosome occupancy data from [36], and construction of Statistical DNA Profiles (SDP) for the consensus clusters. Top panel: heatmaps of nucleosome occupancy data from a tiling array in 4 bp resolution [36], around the transcription start sites (TSS) of the 5,176 yeast genes for which a TSS could be derived from a combination of datasets [68], [99], [100] (see Methods section & Table S2). Original values (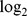 of nucleosomal over genomic DNA signals) varied between –6.25 and 1.66 but were cut at –1.6 and 1.1 for clarity. Genes are sorted by clusters, and within each cluster by their order on the genome, as given by the genome annotation file (SGD, Feb. 2008). Bottom panel: Statistical DNA Profile (SDP) of nucleosome occupancy data. See Methods for details; in short: an SDP of cluster genes shows the cluster mean values (y-axis) at nucleotide positions upstream and downstream (x-axis) of the TSS, in bins of (here) 10 bp (basepairs). The plot symbols reflect the direction of a bias in the distribution of values in *m* cluster genes compared to the distribution of all (*n*) other genes at the given binned position. They were calculated from the relative rank-sums, 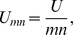 where filled circles indicate a bias towards higher 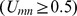, and open circles a bias towards lower 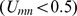 values then the rest of the genome. The plot symbol size scales with the p-value 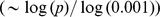 such that the largest symbols represent a significance cutoff at 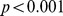 and the smallest a non-significance cutoff at 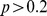. Figure S10 shows the same for all clusters and example distributions at position bin −10 to −1 of the TSS for clusters A & D.

**Figure 5:**
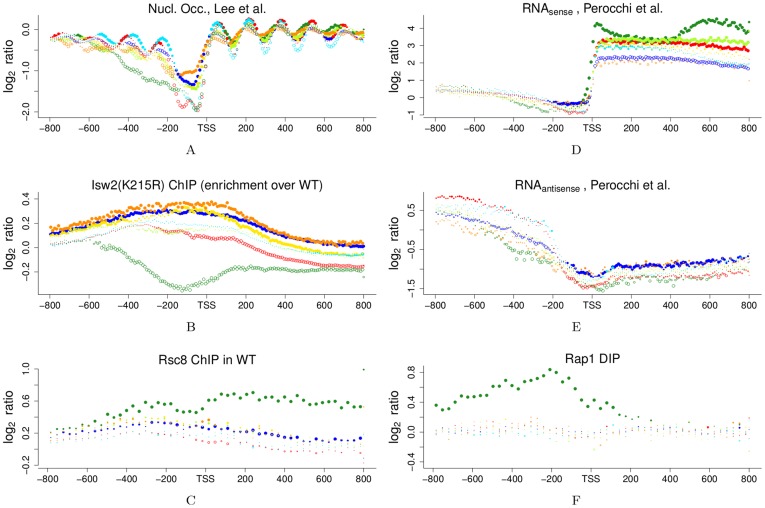
Statistical DNA profiles (SDP) of nucleosome occupancy, Isw2(K215R) ChIP, Rap1p DIP, Rsc8p ChIP & transcriptome tiling array datasets. SDP were constructed as desribed for Figure 4. Figure 1C provides a color legend. Only results for consensus clusters are shown here, see Figure S11 for background clusters. Nucleosome occupancy data from 5A: tiling array dataset in 4 bp resolution [36]; 5B: Isw2(K215R) ChIP-tiling array data in 5 bp resolution [40]. 5D: transcriptome tiling array data in 8 bp resolution [68] on the sense strand; 5E: same as 5D but for the signal from the antisense strand. 5C & 5F: data are from [41] with resolution & SDP bin size: 32 bp; 5C: Rsc8-TAP ChIP-chip data in wildtype cells. 5F: Rap1 DIP-chip data (*in vitro* “DNA immunoprecipitation-chip” of genomic DNA by Rap1p).

Eukaryotic transcription appears to be initiated at NDR [36]. Nucleosome occupancy measurements take a population average, and nucleosomes that have a stable position in many cells give a pronounced signal with shorter distances between adjacent nucleosomes and are often denoted as “well-positioned”, while “fuzzy” positioning refers to a shallower signal with longer distances. Promoters are either found depleted of or occupied by nucleosomes in a given measurement. Four different types of promoter nucleosome configurations were distinguished by k-means clustering of nucleosome profiles around transcription start sites (TSS) [36], and we find highly significant enrichment of clusters with these gene types (Figure 2D). This enrichment can also be clearly seen in a heatmap of nucleosome occupancy data sorted by cluster genes and aligned at TSS, and in position-dependent Statistical DNA Profiles (SDP) of the same dataset (Figures 4 & 5A). Similar patterns can be seen in several other of nucleosome occupancy datasets [37], [40], [65] (Figure S12). Cluster A & C are clearly enriched with genes with wide and narrow NDR, respectively. Both of these classes have arrays of very well-positioned nucleosomes upstream and downstream [36]. Cluster AB genes are strongly depleted of nucleosomes in promoter and downstream regions, and this may result from the very high transcriptional frequencies (Figure S7A) of ribosomal protein genes [36]. Such genes are also significantly enriched in clusters B, B.C & B.D, but at a low percentage (Figure 2D). The heatmap (Figure 4) and statistical profiles (Figure 5A) show that these clusters additionally contain genes with a higher nucleosome occupancy at the promoter, a property shared with clusters B.D & D. Lastly, clusters B.D & D are enriched with genes that are characterized by a fuzzy nucleosome positioning. Thus, a gene classification based solely on the nucleosome configurations around the TSS distinguishes the ribosomal clusters A & C, from metabolic clusters B & D. Moreover, specific properties, such as promoter occupancy, NDR-size and stability of nucleosome positioning, differentiates between the anabolic and catabolic superclusters.

### Differential Chromatin Dynamics: a Candidate Mechanism

Nucleosomes can be shifted laterally along the DNA, away from energetically favorable positions, or evicted completely by ATP-dependent nucleosome remodeling machineries. Two opposing effects of remodeling on transcription have been reported. An ISWI class remodeler (Isw2) shifts nucleosomes from the coding region into the promoter NDR and loss of this activity resulted in de-repression of transcription [40], [66]. In contrast, RSC-type remodelers are required to maintain promoters nucleosome-free and thus transcriptionally competent [42]. The *in vivo* binding sites of Isw2 are thought to be better reflected by the catalytically inactive Isw2(K215R) protein [67], and these are highly enriched around cluster B.C, B.D & D promoters (Figure 5B), and *knock-out* of Isw2 activity results in shifted nucleosome positions for these clusters [40] (Figure 2E). Significant fractions of clusters B.D & D genes are also affected by remodeling of the NDR at the 3′ end of the genes (Figure 2F), which is at some loci required to repress antisense transcription [40], [66]. Indeed, antisense transcription [68] is significantly increased in cluster B.D & D average profiles (Figure 5E). In contrast to repression by Isw2, activity of the RSC complex is required to maintain a promoter NDR and transcriptional competence in many genes. RSC inactivation (by induced intein-splicing) resulted in the collapse of the promoter NDR in 76 of the 136 tested genes on chromosome III [42]. Within this small subset of the yeast genome we still find differential enrichment of cluster genes (Figure S6A), *i*.e., 8 of 12 cluster A 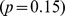 but only 2 of the 11 cluster D promoters 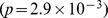 are affected. The strongest enrichment is found for the large low-amplitude cluster l.b (16 of 17 genes, 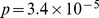), which shares several properties with cluster A, *e*.g., co-regulation in the transcriptome meta-analysis (Figure 3A), a tendency towards broader NDR and enrichment in TFIID-dependent genes (Figures S4C, S10 & S4D). However, an opposite RSC enrichment pattern is found in a previous chromatin immunoprecipitation (ChIP) dataset for several RSC subunits [69] (Figure S6B). High-resolution ChIP data [41] showed that Rsc8p (RSC subunit) is highly enriched in the ribosomal protein genes that comprise cluster AB, still significantly enriched in clusters B, B.C, B.D & D but not enriched in clusters A & C (Figure 5C). Mutations of the highly similar RSC components Rsc3p and Rsc30p have been reported to differentially affect the expression of ribosomal protein (cluster AB), and cell wall component and stress response (enriched in clusters B.D and D, see Table 1) genes [70] (Figures S9A & S9B). Both proteins bind to DNA and recruit RSC to target sites and their proposed binding motifs [71] are slightly enriched in both, AB and B.D promoters, but with low significance (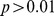, 2.5–4% of genes *vs.* 1–2% genome-wide, Figure S3 & Dataset S6). Similarly, the subunit Rsc9p was found to relocate from genes of clusters AB, B, B.D & D to genes of clusters C & D upon exposure to H_2_O_2_ (all 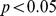, Figures S9C & S9D) [72]. In summary, Isw2 clearly targets clusters B.D & D, while RSC affects both anabolic and catabolic gene groups, but likely with differential outcome or under different conditions.

### Nucleosome Configurations *vs.* Transcriptional States in Mutants

The dataset provided by Badis *et al.* [41] compared nucleosome occupancy and transcript levels in seven temperature-sensitive mutants of different DNA-binding proteins to their isogenic control strains, where both cultures were grown at the restrictive temperature of 37°C. Here we analyze cluster SDP of the relative signal 
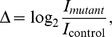
 as provided by the authors, and additionally refer to enrichment of binding motifs or experimental protein binding sites taken from references [71] and [73] (Table S5, Figure S3 and Datasets S5 & S6), respectively. The observed effects may partially be specific and local, *i*.e., in the vicinity of the DNA-binding sites of the proteins, or alternatively merely reflect general stress or a change in growth rate. Moreover, these transcription factors have been called “general regulatory factors” (GRF) that act as insulators for “silenced” histone deacetylation domains, including subtelomeric regions [74], and the mutations may well have genome-wide effects on chromatin structure.

**Figure 6:**
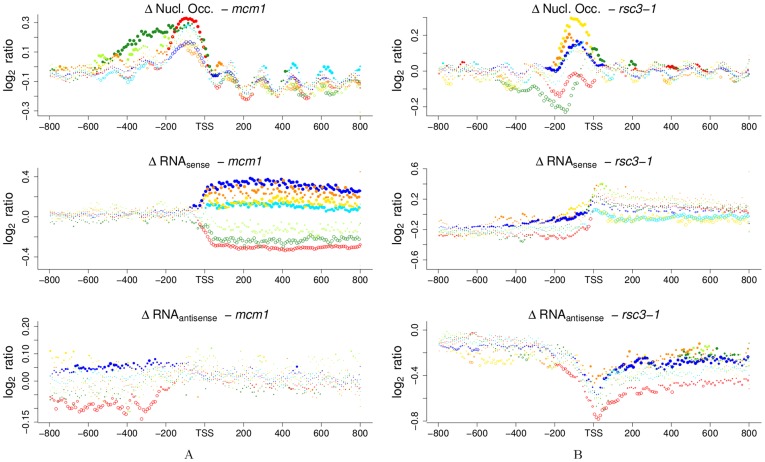
Changes in nucleosome occupancy and transcription in *mcm1-1* and *rsc3-1* strains. SDP plots were constructed as described for Figure 4. Figure 1C provides a color legend. All data are from [41] and were provided (by the original authors) as shown, *i*.e., 
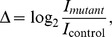
 where *I* are the processed signal intensities from the individual experiments in mutant and isogenic control strains. In all figures, the top panel shows change of nucleosome occupancy from tiling arrays in 4 bp resolution, the middle and bottom panels show the change in transcriptome tiling array signal in 8 bp resolution from the sense and the antisense strands, respectively. 6A: *mcm1-1*; 6B: *rsc3-1*. Results for background clusters are shown in Figures S13 & S18.

The *mcm1-1* (Figures 6A & S13) and *tbf1* (Figure S14) strains showed a typical ESR transcriptional response, *i*.e., cluster A, AB & B are downregulated and clusters B.D & D upregulated. Both strains show a higher average nucleosome occupancy at the promoters of all clusters (all 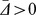 just before TSS), but this increase is significantly lower in the upregulated cluster B.D & D genes and significantly higher in the downregulated clusters A & AB. The change of occupancy in clusters B.C & C is similar between *mcm1-1* and *tbf1* strains, yet, the transcriptome shows a differential response, *i*.e., B.C & C are downregulated in *tbf1* but upregulated in *mcm1-1*, perhaps reflecting the differences between the 0.7 h and the 5 h period cycles (Figure 1). Mcm1p binding sites are slightly enriched in clusters B.C (4% of cluster genes, 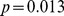), and D (3%, 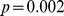), and the binding motif of Tbf1p is enriched in cluster D promoters (21%, 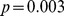). The *cep3*, *abf1-101* and *rap1-1* strains (Figures S15, S16, S17) also show a ESR-like response, but with more subtle features. In *cep3*, the total nucleosome occupancy seems increased over the control strain, indicated by 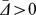 in all clusters over the complete analyzed range, but the occupancy increase is significantly higher in promoters of clusters A, AB & C. Clusters B.C & C are uncoupled from the ESR and downregulated. Cep3p binds to centromers and we find no enrichment of it’s binding motif in any cluster. In contrast, Abf1p binding sites are highly enriched in cluster A (11%, 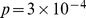) and Rap1p in clusters AB (50%, 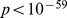, Figure 5F). Thus, the strong downregulation of cluster A in *abf1-101*, and of AB in *rap1-1* may in part be related to specific and local effects of these proteins. In both mutants, nucleosome occupancy of cluster AB promoters is strongly increased, and we observe an increase of transcription upstream of the TSS, a moderate downregulation at the 5′ end, most likely stemming from the introns that are enriched in 5′ regions of these ribosomal protein genes, and strong downregulation 3′ of this intronic region. And lastly, nucleosome occupancy at the promoters of clusters A & AB is significantly decreased in the *rsc3-1* (Figure 6B) and *reb1-212* (Figure S19) strains, but without concurrent increase in transcript levels, suggesting that these growth clusters are highly expressed in the control strains. Clusters B, B.C, B.D & D have increased nucleosome occupancy in *rsc3-1*. While in the *reb1-212* mutant all clusters show a slight global decrease in nucleosome occupancy just before the TSS (all 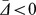), the decrease is less in clusters B, B.C, B.D & D. Only the mitochondrial clusters B.C & C are significantly downregulated in both mutants. In the *rsc3-1* strain, clusters B.C, B.D & D all show increased transcription upstream of the TSS (Figure 6B, middle panel). The signal from the antisense strand of this mutant is generally lower than in the control strain (all 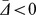, right of TSS), but the decrease is significantly less in clusters B.D & D compared to other clusters (Figure 6B, bottom panel). A unique uncoupling of clusters B.D and D was observed in the *reb1-212* strain where only B.D is significantly upregulated, coinciding with an unusual signal peak of the intronic region of cluster AB genes. This may result from premature transcription termination, indicated also by small peaks around the TSS of all clusters. In summary, the observed effects reach well beyond specific promoter binding sites of the tested set of GRF mutants, implying a stress-response or change of growth rates in these cell lines, accompanied by genome-wide remodeling of chromatin structure. The mutant cell lines tested by Badis *et al.* [41] thus clearly show, that distinct nucleosome occupancy states are indeed associated with transcriptional states akin to the transcriptional phases observed during synchronized respiratory cycling of budding yeast cell cultures.

## Discussion

**Figure 7:**
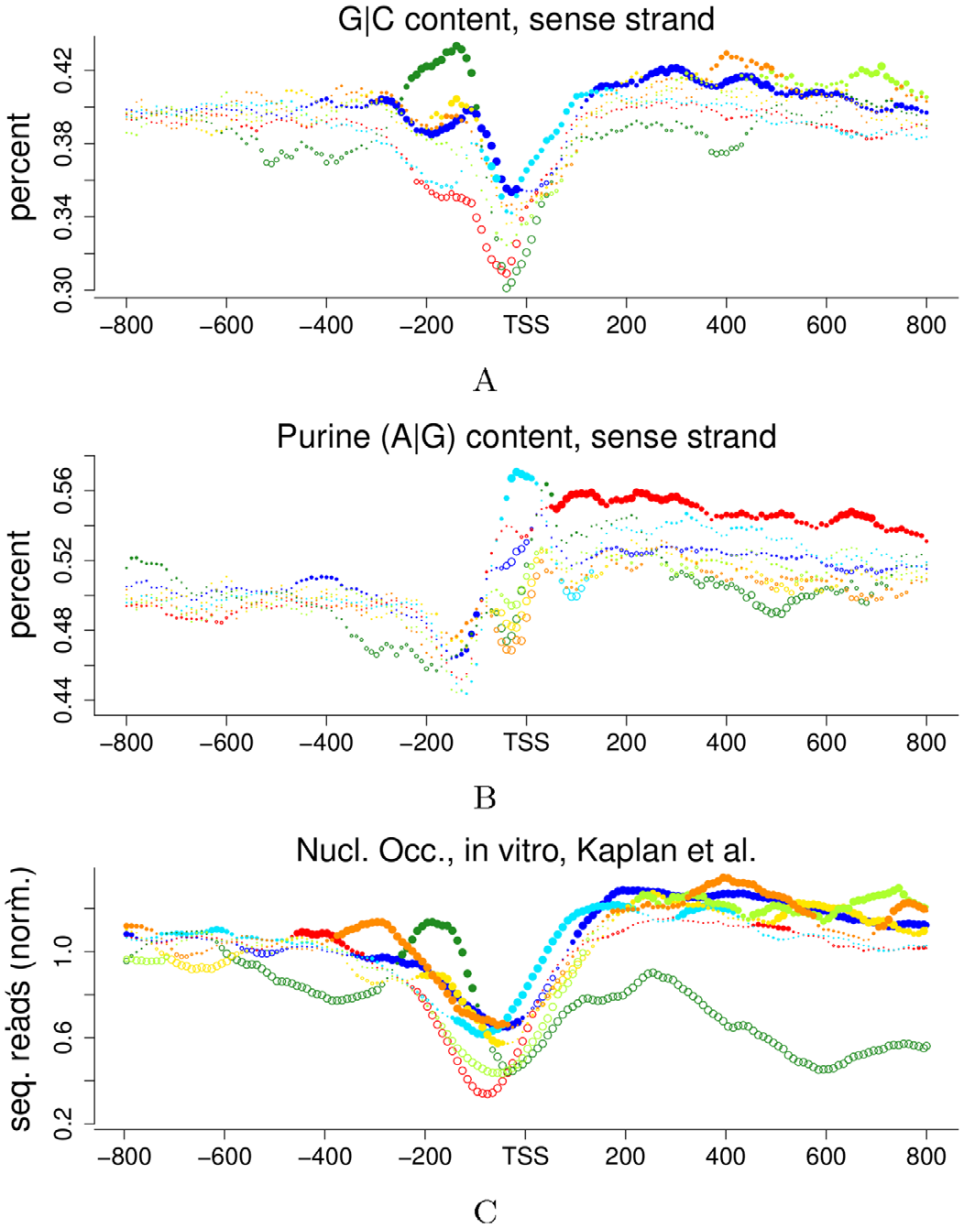
Summary of results & proposed feedback model. 7A: temporal flow of expression and functional relationships of cluster transcripts in the 0.7 h system (left to right) and the 5 h system (top to bottom). 7B: summary of observed properties (significant enrichment or biases) of the main gene clusters. 7C: Potential regulatory interactions of broad cellular functionality *via* the energetic status of the cell, reflected, *e*.g., in ATP:ADP ratios. In the oxidative phase catabolic activity leads to a high ATP synthesis rate. At high ATP:ADP ratios promoters of anabolic genes are active, potentially mediated by ATP-dependent nucleosome remodeling, which at the same time keeps promoters of catabolic genes in a repressed state. When respiratory activity suddenly slows down in the reductive phase the activity of the anabolic genes, *i*.e., amino acid and protein synthesis, leads to a decrease of the ATP:ADP ratio and the promoters of catabolic genes become active. Diverse cellular stresses may result in a sudden drop in the cellular ATP:ADP ratio due to the energetic costs of immediate biochemical stress response.

In this work, we have identified seven consensus clusters of genes, whose transcripts show periodic time-series during both, the 0.7 h [11] and the 5 h [10] period respiratory oscillations. Specifically, clusters A, AB, B, C and D define a common temporal gene expression program (Figures 1 & 7A). Their relation to respiratory activity and their functional enrichment profiles (Tables 1, S3 & S4) support a distinction of two superclusters. The cell growth supercluster (A
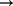
AB
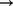
B) is expressed during the oxidative phase, and the energy-mobilizing supercluster (C
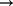
D) is expressed in the reductive phase. Each supercluster develops from predominantly TATA-less and TFIID-controlled genes that encode for ribosome biogenesis (A/AB: cytoplasmic or C: mitochondrial), to gene groups that are enriched in TATA Boxes and SAGA-control and encode for metabolic functions (B: amino acid synthesis or D: catabolism and stress-response) (Figure 7B).

Clusters C and D are co-expressed in the 0.7 h but anti-phase in the 5 h system, accompanied by differential regulation of the amphibolic core carbon backbone of metabolism and DNA replication machineries in clusters B.C and B.D. These differences may be due to differential extent of S-phase synchrony (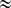
10% or 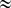
50%) in the two systems. This difference is reflected in differential association of average cluster C transcript levels in the transcription factor mutant dataset of [41] (*e*.g., Figures 6A *vs.* S14). Genes encoding for mitochondrial functions, *i*.e., cluster C, were switched from wide (cluster A-like) to narrow NDR configurations, concurrent with the evolution of the respiro-fermentative lifestyle after a whole genome duplication event [75], and could also be distinguished in a detailed analysis of stress-response cohorts [32]. In line with the direct feedback interactions discussed below, mitochondrial activity, reactive oxygen species or, more specifically, NAD^+^-mediated regulation of both chromatin [76], [77] and the flux direction along the core carbon backbone [78]–[80] may well play a role for the differential regulation. Further data on systems with different extent of S-phase synchrony or an experimental system to reproducibly vary the oscillation periods will be required to go beyond this only descriptive discussion of the differences between the two analyzed systems.

Common to both systems, however, is the antiphase relation of the two superclusters. This and their anti-correlation in our transcriptome meta-analysis (Figure 3A) and the correlation with the “environmental stress response” [20], [31] (Figures 2A & 2B) point to a common regulator with opposing effects on the expression of the two superclusters. A detailed analysis of the functional annotations of co-regulated gene groups lead to an interpretation of the stress response as a general reaction to energy-limitation, where the costly translation program is downregulated and concurrently energy-mobilizing processes are upregulated [32]. We have previously shown that various measures of the energetic flux of the cell strongly vary over the cycle, *e*.g., the cytochrome oxidation state and mitochondrial morphology [17]. Anabolism is, however, energetically driven by a concentration gradient between ATP and ADP. We report here an oscillation of the ATP:ADP ratio (Figure 3B) that is compatible with this energetic interpretation of the stress response. When ATP:ADP is high (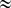
5–6), the growth supercluster is expressed. A subsequent activity of this growth program, concurrent with low respiratory activity, would explain the decrease of the ATP:ADP ratio in the reductive phase (down to 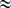
1–2). This phase is paralleled by increase in expression of catabolic and respiratory genes whose activity subsequently would replenish ATP in the next cycle. These consequences of the metabolic activity of the two superclusters are depicted as positive or negative influence on ATP in Figure 7C. Could, in turn, the energetic state or specifically the ATP:ADP ratio directly and differentially feed back on the expression of the anabolic and catabolic superclusters?

**Figure 8:**
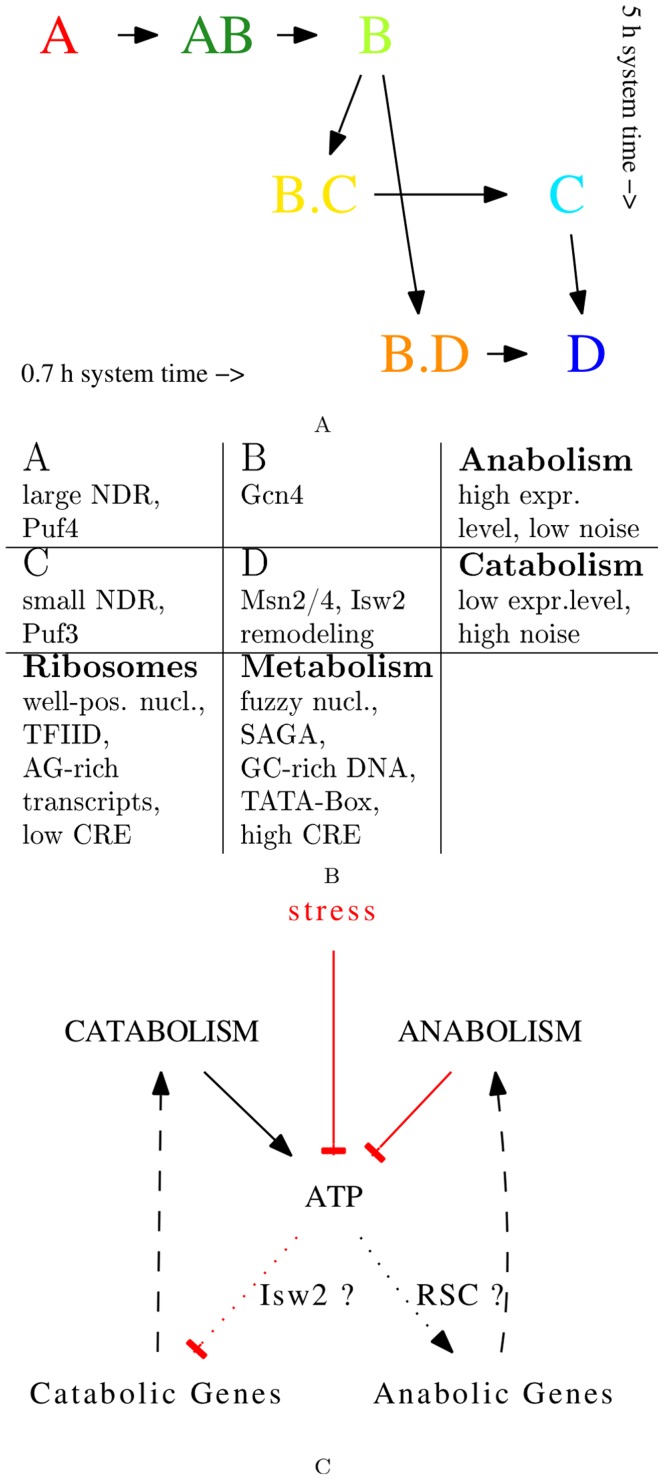
Nucleotide content & *in vitro* nucleosome occupancy. Figure 1C provides a color legend. 8A & 8B: local GC- and purine frequencies were first calculated for each gene and each position in sliding windows (size: 71 nt.), and then SDP were constructed using *t*-tests for statistics visualization. Tests were performed without prior binning of values, and instead values are shown only for each 10^th^ nucleotide position for visual clarity. 8C: *in vitro* nucleosome reconstitution at low histone levels [37], 1 bp resolution, SDP with bin size 10 bp and Mann-Whitney-Wilcoxon tests. Background clusters are shown in Figure S20.

Such a direct feedback between energetic state and gene expression is known from bacteria, where the ATP:ADP ratio correlates with the extent of negative supercoiling that is introduced by ATP-dependent gyrase [47], [48] which in turn differentially affects transcription of the gene encoding for the gyrase [81] and for anabolic and catabolic enzymes [50]. While in *Escherichia coli* the resulting feedback was interpreted in terms of a homeostatic regulation system, rhythmic changes in DNA structure were observed over the circadian cycle of the cyanobacterium *Synechococcus elongatus* PCC7942 [52]. Negative supercoiling is increased during the photosynthetic phase and is required for transcription from GC-rich genes [53]. In our system, all clusters are significantly enriched in one of four distinct promoter nucleosome configurations (Figures 2D & 5A) [36]. Nucleosome occupancy partially depends on sequence properties, *e*.g., the GC-content [38]. Cluster A transcripts are purine-rich and cluster D genes are GC-rich (Figures 8A & 8B). Thus, the clusters may differ in sequence-dependent “default” nucleosome configurations or overall occupancy, which is also reflected in the differential *in vitro* occupancy (Figure 8C) [37] and could lay the grounds for differential regulation. A candidate mechanism is ATP-dependent nucleosome remodeling, where ATP hydrolysis provides the mechanical force to generate negative superhelical torque [82] and break DNA-histone contacts [83]. The addition of ATP to naked DNA, histones and cell extract allowed the *in vitro* reconstitution of *in vivo* promoter nucleosome configurations, suggesting a major role of ATP-dependent remodeling in the establishment and maintenance of different types of promoter nucleosome configuration [43]. The differential consequences of promoter nucleosome remodeling by the RSC- and Isw2-types of remodeling machineries, and their differential association with cluster genes (Figures 2E, 5B, 5C, S5, S6 & S9) elegantly complement the proposed feedback model between anabolic and catabolic pathways (Figure 7C). At high ATP:ADP ratio, RSC would keep promoters of anabolic genes open and competent for transcription, while Isw2 would actively repress catabolic gene promoters. When the ATP:ADP ratio drops both remodelers may become less active, and gene expression would switch from growth to catabolic genes. ADP promotes the dissociation of Isw2 from DNA [84], further supporting a direct influence of the ATP:ADP ratio. In this scenario, ATP-dependent nucleosome remodeling literally gates gene expression by opening or closing promoter regions apt to the current energetic state of the cell. However, the diverse targets of RSC remain elusive and are difficult to establish experimentally [42]. Interestingly, the step-length of RSC-mediated remodeling, *i*.e., the distance over which a given nucleosome is moved along the DNA in one remodeling cycle, has recently been observed to depend on the ATP concentration *in vitro* [85], which *in vivo* could lead to differential rotational positioning, and thus exposure or covering, of regulatory motifs [86] such as the TATA-Box in the metabolic cohorts B and D of the two superclusters. Oscillating levels of acetyl-CoA- and SAGA-dependent histone acetylation have been found to enable rapid transcription of growth genes (clusters AB, B) in the oxidative phase, while the SAGA complex binds to stress-regulated genes (D) during the reductive phase of a 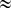
5 h oscillation [87]. Thus, RSC and SAGA, or ATP-dependent nucleosome remodeling and acetyl-CoA-dependent histone acetylation, may cooperate [88] at both anabolic and catabolic gene clusters, and relate the metabolic state of the cell to an appropriate transcriptional output.

The combined dataset provided by Badis *et al.* [41] clearly shows that indeed differential promoter occupancy of the cluster genes is associated with differential transcript levels (Figures 6 & S13, S14, S15, S16, S17, S18, S19), where the observed effects reach well beyond local binding sites of the tested mutants of DNA-binding proteins. The diverse detail observations in this dataset point to further processes involved. Upstream non-coding and antisense transcription around the stress-activated clusters D and B.D indicate a role of noncoding RNA transcription [89], [90], potentially in transcriptional silencing [40], [91], [92]. And finally, the global bias in nucleosome occupancy (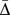
Nucl.Occ.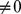 in all clusters) or positioning (periodic 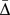
Nucl.Occ. downstream of TSS) in some of the mutants may point towards genome-wide chromatin re-arrangements. We interpret this as further strong evidence of genome-wide chromatin remodeling cycles and complex transcriptional landscapes during the respiratory oscillation.

In summary, our systematic statistical comparison of large data compendia provide an integrated perspective on the possible interactions between metabolism, chromatin structure and transcription. Such direct links between central metabolism and chromatin dynamics have recently been implicated also in mammalian regulatory systems such as the circadian clock [44] and cancer [45], [46]. Here, we proposed an analogy in prokaryote systems, *i*.e., the circadian supercoiling dynamics in cyanobacteria [52], [53]. For the case of respiratory oscillation in yeast continuous culture, we defined a gene expression program (Figure 7A) that is coherent in both, function and time, and proposed a first mechanistic interpretation of not only the oscillatory gene expression common to the 0.7 h and 5 h systems, but also for the often observed, yet still enigmatic stress response of transcription (Figure 7C). We expect that ATP-dependent nucleosome remodeling plays an important role, most likely in interaction with the co-factor dependences of post-translational histone modifications [87]. It has recently been proposed that even in the absence of culture synchrony, individual cells may always undergo an oscillatory growth program, and that a given sample merely reflects a mixture of cells that are in either the reductive or the oxidative phase. An observed stress response would then just reflect a decreased overall growth rate where individual cells remain longer in the reductive phase [20]. This would require a re-interpretation of all previous experiments on steady-state and batch cultures, including all chromatin-structural data analyzed herein. Our analysis and interpretations are fully compatible with this hypothesis. Time series data on chromatin structure over the respiratory cycle will be required to understand the dynamics of local and global chromatin and transcription landscapes. We predict that oscillatory continuous culture will become an invaluable experimental system for an integrative mechanistic understanding of both chromatin biology and growth regulation, since the synchronized culture naturally cycles between transcription from genes with both, complementary functions in cellular growth, and differential chromatin structure and dynamics.

## Methods

Automated data collection and preprocessing were handled by scripts in the Perl programming language. All statistic analyses and data visualization were performed using the R statistics package, version 2.11.

### Microarray Processing

Time series data from the two microarray experiments were based on the Yeast_2 (0.7 h period dataset) and the YG_S98 (5 h period dataset) Affymetrix microarrays. Raw data was obtained from microarray image files directly (R package affy, without background or mismatch correction, or normalization), using the FARMS summarization method [93] (parameters: weight = 0, µ = 0, with “robust” and “weighted mean settings” settings active). Since several properties of the respiratory oscillation may lead to a violation of central assumptions underlying common experimental and computational normalization procedures, raw data was used for Fourier analysis and clustering. A custom-made normalization, akin to a previously suggested strategy [94] but adapted for periodic data, was used only for clarity of visual display of the average cluster time courses (Figures 1A and 1B). Text S1, section S1.1, provides a more detailed discussion of these problems and the chosen normalization strategy. The files Yeast_2.na27.annot.csv and yeast2_best_match.txt, as provided by Affymetrix, were used to map the datasets to the 5,795 protein-coding genes annotated in our reference genome release (Feb. 2008 SGD release), resulting in 5,571 (0.7 h) and 5,315 (5 h) individual time series. The raw time series data are available in Dataset S1.

### Discrete Fourier Transform (DFT)

A time series of *N* measurements 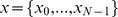, taken at equally spaced measurement time points 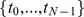, can be approximated in frequency-space by applying the Discrete Fourier Transform (DFT):
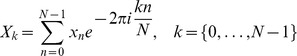



where *X* is a vector of complex numbers representing the decomposition of the original time series into an offset value (at *k* = 0, also known as “direct current” DC in signal processing) and a series of harmonic oscillations around this offset with periods 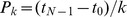. Amplitude 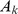 and phase angle 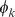 at a given DFT component *k* can be calculated as 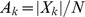 and 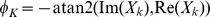. The index *k* corresponds to the number of full cycles with period 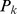 in the time series. The two experiments analyzed here were taken over 4 and 3 full cycles of the respiratory oscillation, and we define the number 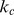 of phenotypic cycles (here indicated by dissolved O_2_ concentration, but in other scenarios the phenotypic cycle could be the cell division or a circadian cycle), where 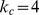 for the 0.7 h and 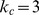 for the 5 h period dataset. The amplitude 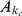 corresponds to previously used measures of periodicity in mRNA time series [54], [55], [95]. Additionally, the phase angle 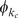 is a good approximation of the peak time of a given transcript’s abundance within the cycle.

The microarray fluorescence intensity depends on sequence-specific hybridization energies. Thus, individual time series are usually interpreted relative to their mean signal (commonly as the 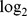 of the mean-ratio, 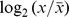). For our purpose, a similar normalization in the frequency domain proved useful: the scaled amplitude 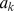 is the amplitude at cycle number *k* divided by the mean of amplitudes at all other non-zero cycle numbers (except the “half-sampling” or Nyquist frequency at N/2), 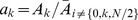. Phase angles 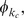 scaled amplitudes 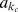 and p-values 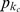 from a permutation test (see Text S1 for details) are available in Dataset S1.

### DFT-based Clustering

Based on the observed DFT spectra and general considerations of DFT properties, the cycle numbers 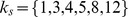 and 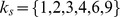 were selected for clustering analysis of the 0.7 h [11] and the 5 h [10] period datasets, respectively. Text S1, section S1.2, outlines the reasoning underlying our DFT component selection. The scaled real and imaginary parts of these components were re-calculated from phase angles 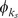 and scaled amplitudes 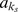. The model-based clustering algorithm flowClust [56] (with default parameters of its R library, version 2.6.0; 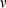 = 4, 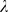 = 1, 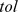 = 1e-5) was applied to these 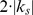-dimensional datasets. Text S1, section S1.3, gives a detailed account on the reasoning behind data processing and the choice of this algorithm. The algorithm is based on *t*-mixture models with a Box-Cox transformation and an expectation-maximization algorithm handles optimization of the parameters of the *t*-distributions and the data transformation (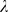) simultaneously [96]. The Box-Cox transformation parameter remained close to 1 for both datasets, 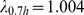 and 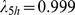. The optimal number of clusters in each dataset was evaluated by the Bayesian Information Criterion, as outlined in the flowClust publication [56], and by 2-objective plots of variance and connectivity [97] of the original time series (as the 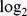 mean ratio), but the final decision was based on visual inspection of the clustered raw time series data. The clustering algorithm involves random partitioning of the data for its initialization procedure and therefore the final cluster assignments and BIC development depend on the order of the input data (originating from the order of probes on the array). Thus the order employed is given in the Dataset 9.0.1 to ensure full reproducibility.

The two individual clusterings were then sorted by their circular phase angle density peaks at cycle number 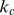 and re-labeled accordingly. For convenience, all phase angles 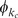 were shifted before this sorting such that the later cluster A transcripts are just above 0° in both datasets (Figure 1). This phase shift does not affect the clustering, since the data is correctly treated as circular. The significance of overlaps between the two clusterings was established by cumulative hypergeometric distribution tests and guided the definition of the final consensus clusters (Figure S1). This manual step accounted for the higher temporal resolution of the 0.7 h period dataset (4 min), *e*.g., the rapid transition from clusters A to B are well resolved in this dataset but mixed in the 5 h period dataset (25 min sample resolution). The latter dataset thus served mainly to define a consensus gene set, *i*.e., to filter potentially mis-associated outliers of the two individual clusterings (as an alternative to p-value cut-offs) and to identify gene groups that are differentially regulated between the two systems, *i*.e. C *vs.* D, B.C and B.D. The original DFT-based clusterings and the final overlap clustering are available in Dataset S1.

### Genome Data Sources

The main gene list and genome sequence underlying this analysis is based on the Sacchormyces Genome Database (SGD) [98] release from February 2, 2008, featuring 5,795 *bona-fide* protein-coding genes. Outdated gene IDs in analyzed datasets were updated or removed, and coordinate-based data were aligned to this genome release by accounting for coordinate changes (insertions and deletions) between the genome release underlying the respective dataset and the release used herein, as defined in the online annotation history at http://yeastgenome.org/. When a downloaded gene list contained multiple entries for a given gene (*e*.g., as a result of the employed microarrays or of gene merging in the annotation history), the first entry was taken. Continuous and categorical gene data analyzed in this work is available in Dataset S7. Coordinate-based datasets, aligned to the genome in the SGD release from Feb. 2008, are available at http://www.tbi.univie.ac.at/raim/data/2011/yeast/clusters/geneData.tar.gz and Table S8 maps data IDs, SDP plot labels and the original publications. Table S6 gives the URLs where the data were downloaded from. Table S7 further lists the yeast strains that were used in the respective studies.

### Transcription Start Sites (TSS)

TSS coordinates were collected from three different sources [68], [99], [100] and weighted centers of multiple start sites within windows of 73 nucleotides (ca. half a nucleosome length) were calculated as consensus positions. Then the site closest to a gene’s start codon (within –400 nucleotides upstream) was used as the TSS. Consensus TSS for 5,176 protein coding genes could be defined (Table S2) and are available in Dataset S7.

### Statistical Analyses, Categorical Data

The overlaps between the initial clusterings of the two datasets as well as the overlap of the final clusters with other gene classifications were analyzed by cumulative hypergeometric distribution tests. Given *m* genes in a certain cluster (*e*.g., 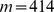 genes in cluster A), we can calculate the probability 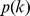 of finding at least *k* genes of this cluster within the *n* genes of a test category (*e*.g., *k* = 68 of *n* = 240 genes with positive growth rate correlation, Figure 1) drawn from all 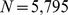 protein-coding genes as 
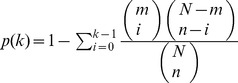
. The enrichment *E* of the tested category in the given cluster is the ratio of the frequency in cluster genes over the genomic frequency: 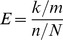, where 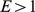 if the cluster has a higher frequency of genes of the tested category then the total genome.

### Statistical Analyses, Numerical Data

A bias of the distribution of numerical data between *n* genes of a given cluster and 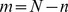 genes of the rest of all genes in our analysis (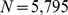, or less if data was not available for all genes) was analyzed by two-sided Mann-Whitney-Wilcoxon tests, where probabilities (p-values) were calculated using the Shift-Algorithm by Streitberg & Röhmel (see R function wilcox.exact). The normalized test statistic, 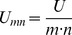, where *U* is the rank sum, was calculated when the direction and extend of a bias was of interest, *i*.e., for Figure 3A and all SDP figures. 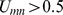 if the tested cluster tends to higher values then the rest of genes, and 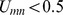 otherwise. For normally distributed data, *i*.e., the nucleotide frequencies in Figure 8, a Welch’s *t*-test was applied. The *t*-value indicates the direction of the bias, *i*.e., 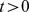 or 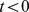 for higher or lower values in the cluster then in all other genes, respectively.

### Functional Analysis

We use a very basic analysis of gene ontology (GO) annotation, based on cumulative hypergeometric distribution tests of only the direct annotations given in the SGD genome annotation file, which contains in total 3107 unique GO terms. We do not take into account the directed acyclic graph structure of GO, *i*.e., we do not propagate annotation terms upwards in this GO structure. While this approach may miss enriched lower level annotations in clusters that consist of very well defined genes, *e*.g., “amino acid biosynthesis” in cluster B, it avoids to miss higher level GO annotations, such as the “unknown” categorizations in cluster D. The full results of the GO analysis are available as Dataset S2.

### Statistical DNA Profiles (SDP)

High-resolution data of DNA structure, such as tiling arrays of protein-bound DNA fragments, local nucleotide content or positions of small sequence motifs, are often analyzed by aligning a given group of genes at a specific site, *e*.g., experimentally derived transcription start sites (TSS), start or stop codons of the coding region, and calculating average values at positions upstream and downstream of this alignment site. The values can be binned over a range of bases surrounding the given position. For an SDP this simple approach is extended by visualizing the results of individual statistic tests, where the distribution of values of a certain group of genes (a cluster) is compared with the distribution of values of all other genes of the genome at each position (or bin). For numerical data Welch’s *t*-tests are used if the values are normally distributed and Mann-Whitney-Wilcoxon tests otherwise. For categorical data cumulative hypergeometric distribution tests could be applied. The symbol type of the individual data points indicate the direction of the bias, *i*.e., whether the respective cluster gene distribution is higher (filled circle) or lower (empty circle) than the rest of the genes, which can be readily derived from the *t*-value of a *t*-test, the normalized rank sum 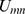 of a rank sum test or the enrichment *E* for categorical data (see paragraphs on “Statistical Analysis” above). Additionally, the symbol size scales with the test’s p-value, 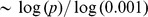, such that the largest symbols represent a significance cutoff at 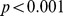 and the smallest a non-significance cutoff at 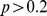. Smaller clusters often are less significant at the same average value as a larger cluster. We thus plot clusters in order of decreasing size (number of genes) to avoid hiding smaller plot symbols behind those of larger clusters.

The SDP de-emphasize regions where a cluster’s distribution does not deviate (significantly) from the rest of the genome which increases the plot clarity and allows to inspect multiple clusters in one plot. On the other hand, an SDP allows to directly compare a given clusters’ average profile with the genomic average. For example, when applied to the periodic nucleosome occupancy data, an SDP indicates whether a given nucleosome is shifted upstream or downstream in the tested gene group compared to the average configuration in rest of the genome, or can reveal the relative regularity of nucleosome positioning in a cluster. The Figure S10A compares such an SDP (bottom panel) with the occupancy of individual genes visualized in a heatmap (top panel), and Figures S10B & S10C show the full distributions for clusters A and D at a given coordinate.

A large archive of all data underlying the SDP plots is available at http://www.tbi.univie.ac.at/raim/data/2011/yeast/clusters/geneData.tar.gz and and Table S8 maps data IDs, SDP plot labels and the original publications.

### DNA Sequence Motifs and Protein:DNA Binding Data

A collection of DNA binding motifs, either as position weight matrices (PWM) taken from [71] or as IUPAC consensus sequences from diverse sources (Dataset S4), was used to search for motif occurrence upstream and downstream of genes. For position weight matrices, a simple cut-off at 80% of the maximum score of the given PWM was used. The search range was 500 nucleotides either upstream of the START or downstream of the STOP codon of the respective gene. Except for motifs called “TATA.350” (between START and −350; a commonly used range for TATA Box discovery) and “TATA.500” (between −351 and −500). A search range downstream of the STOP codon is indicated by the suffix “.3p”.

Experimental transcription factor binding sites were taken from [73], using the set without any conservation constraints and at a p-value cutoff of 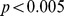, and a search range between −600 and +100 nucleotides of the START codon (the array employed by the original authors featured only promoter probes). Additionally a binary classification of binding data for Rap1, Sir2, Sir3, and Sir4 proteins from [101] was used (indicated by the suffix “.lieb01”), where the original authors distinguished binding to coding, intron or intergenic sequences; here, the latter two are indicated by prefixes “in” and “ig”/“ig2”, respectively. The percentage of cluster genes containing a given site or motif, and enrichment *E* over the genomic fraction are plotted in Figure 11. To test for significance of enrichment, cumulative hypergeometric distribution tests were applied and all motifs and sites with a p-value 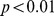 are shown in Table S5. All values, enrichments and p-values are given in Datasets S5 & S6.

### Strain, Culture Techniques, ADP and ATP Measurement

The strain used for this study was *Saccharomyces cerevisiae* IFO 0233. All cultures conditions were the same as described in the supporting methods of [8]. Unless otherwise stated all chemicals were supplied by Wako Pure Chemicals Industries. Metabolites were extracted by mixing culture (1 mL) with perchloric acid (0.150 mL, 60%) and TRIS-HCl (333 mM; 0.450 mL; pH 7.4) (adapted from [102]) in a 1.5 mL tube. Tubes were incubated for 1 h at 0°C on a rotary mixer (5 rpm). The extraction was halted by neutralizing using 10 N KOH. The samples were then centrifuged at 12,000×g for 5 min at 0°C. Aliquots of samples (0.1 mL) were stored at −80°C until analysis. Standards of ADP or ATP (0.01–10 µM) were prepared by adding 1 mL of standard with perchloric acid (0.150 mL, 60%, Wako Pure Chemicals Industries) and EDTA (200 mM; 0.450 mL) in a 1.5 mL tube. Tubes were incubated for 1 h at 0°C on a rotary mixer (5 rpm). Standards were then neutralized using 10 N KOH. The samples were then centrifuged at 12,000×g for 10 min at 0°C. Aliquots of standards (0.1 mL) were stored at −80°C until analysis. ADP was first converted to ATP enzymatically (adapted from [103]). Briefly an aliquot (0.1 mL) or ADP standard was mixed with 50 µL reaction buffer. Reaction buffer comprised of 50 mM phosphoenol pyruvate, 100 mM TRIS-HCl (pH7.4), 35 mM KCl, 6 mM MgCl_2_ and 150 IU/mL pyruvate kinase. The reactions were incubated at room temperature for 1 h. ATP samples and standards were treated in a similar way except the reaction buffer did not contain 50 mM phosphoenol pyruvate. This yielded two sample sets one consisting of native ATP and one consisting of native ATP + ADP converted into ATP from the enzymatic conversion. [ATP] and [ADP+ATP] in µM were then measured using a luciferase assay kit (Kinsiro) as per manufacturer’s instructions. Measurements were carried out in black 96-well microplates (promega) using a Luminescence Microplate Reader (SpectraMax M5e, Molecular Devices). ATP:ADP ratios were calculated thus:
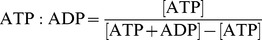



The measured ATP:ADP ratios and the dissolved O_2_ concentration during the measurement are available in Dataset S8.

## Supporting Information

Figure S1
**Overlap table of the two individual clusterings.** 8 & 8: Individual flowClust clusterings of microarray fluorescence time series (shown is the log-ratio of raw data) from the 0.7 h (8) and 5 h (8) systems, after sorting by (phase-shifted) circular density peaks of the phase angles 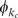 and re-labeling. The y-axis labels give the cluster assignments and the number of genes in each cluster. The thick and thin colored lines are the cluster mean and upper and lower quartiles, respectively, and gray lines are individual transcript time series. 8: Overlap table of the two individual sorted and re-labeled clusterings. For this plot, non-oscillatory clusters (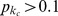 in 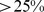 of cluster genes) were additionally moved to the end, just before the not-on-array clusters “r”, *i. e.*, cluster 4 in the 0.7 h system, while clusters 7 & 8 in the 5 h system did not require this step. The first row in each field gives the final cluster assignments used in this work, the middle row gives the number of genes in each field, and the bottom row gives the p-value from cumulative hypergeometric distribution tests. The p-values are additionally indicated by the gray-scale of the fields (see legend on the right axis). All clusterings are available in Dataset S1.(TIFF)

Figure S2
**Normalized cluster time courses.** Individual cluster time courses. Individual time courses of microarray fluorescence (as 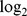 of the mean-ratio) of the final overlap clusters. The thick and thin colored lines are the cluster mean and upper and lower quantiles, respectively, and gray lines are individual transcript time series. S2A: 0.7 h period system [11] and S2B: 5 h period system [10]. Normalization was performed with “least-oscillating” gene sets as normalization reference, see Text S1 for details. The raw data is available in Dataset S1.(TIFF)

Figure S3
**Transcription factor binding sites and motifs.** Transcription factor motifs (10) and experimental binding sites (10), counts per cluster. Motifs and binding sites were obtained as described in the Methods section of the main article. Numbers give the percentage of cluster genes which have at least one occurrence of the given motif or protein binding (suffix “.3p” indicates occurrence downstream of the STOP codon). The enrichment *E* (see Methods) is color-coded, with a cut-off at 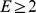. Rows were ordered by clustering the *E* values with hclust [105]. Table S5 lists all cluster motif/site combinations with a p-value 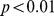 in cumulative hypergeometric distribution tests and Datasets S5 & S6 provide all results. For binding sites and motifs associated with a specific proteins, the cluster assignments of the respective transcripts are shown as row colors.(TIFF)

Figure S4 **Overlap of the consensus clusters with promoter classes, and stress & growth rate response genes.** As Figures 2A–2D of the main article, but for all clusters. All data are available in Dataset S7.
(TIFF)

Figure S5 **Isw2-bound and affected genes.** As Figures 2E & 2F of the main article but for all clusters. All data are available in Dataset S7.
(TIFF)

Figure S6
**RSC-bound and -affected promoter classes.** S6A: promoters on chromosome III were “affected” or “unaffected” (or not analyzed, “NA”) upon inactivation (by induced intein-splicing) of Sth1, the catalytic component of the RSC complex, from [42]. S6B: genes bound by the RSC complex defined *via* a “combined p-value” calculated from several complex components in [69], “TRUE”: 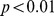 and “FALSE”: 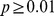. All data are available in Dataset S7.(TIFF)

Figure S7 **Transcriptional frequency, noise & growth-rate.** Statistical biases that distinguish anabolic from catabolic superclusters. Cluster distributions are shown as bean-plots [106]. S7A: transcriptional frequencies, data from [107]; S7B: numbers of proteins per cell, data from [108]; S7D: transcriptional noise, data from [61]; S7C: correlation of expression with growth rates in nutrient-limiting conditions, data from [31]. Two-sided Wilcoxon rank-sum tests were applied to compare the distribution of *n* values in each cluster to the *m* values of all other genes. The number of cluster genes (*n*) for which a value was available in the given dataset is shown in the bottom row, and the total number of available values (*m* + *n*) is shown on the right y-axis. The dotted and solid lines show the total and cluster medians, respectively. The resulting p-values are shown above each plot and the text color indicates whether the cluster distribution is higher (black) or lower (red) then the distribution of the respective rest of the genome. All data are available in Dataset S7.
(TIFF)

Figure S8 **mRNA half-lives and Chromatin Regulation Scores.** Statistical biases that distinguish ribosomal from metabolic superclusters. Same as Fig. S7 but for S8A: RNA half-lives, data from [109]; and S8B: chromatin-regulation score (CRE), data from [64]. Axis annotations as described for Fig. S7. All data are available in Dataset S7.
(TIFF)

Figure S9
**Expression in** ***rsc3-2*** **and** ***rsc30*** 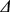 **strains and Rsc9p location.** Change of transcript levels in strains carrying the *rsc3-1 3-2* (16) and *rsc30* 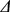 (16) mutations; data from [70]. Rsc9p binding in untreated (16 and H_2_O_2_-treated cells, from [72]. Axis annotations as described for Fig. S7. All data are available in Dataset S7.(TIFF)

Figure S10
**Nucleosome Occupancy: Heatmap and SDP construction.** S10A: as Figure 4 of the main article, but for all clusters. Figures S10B and S10C show distrubtions and test results for the bin between positions -10 and -1 (from the TSS) for clusters A and D, respectively. The “relative W” value corresponds to 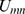.(TIFF)

Figure S11 **Statistical DNA profiles (SDP) of nucleosome occupancy, Isw2(K215R) ChIP, Rap1p DIP, Rsc8p ChIP & transcriptome tiling array datasets.** Same as Figure 5 of the main article, but for background clusters.
(TIFF)

Figure S12 **Statistical DNA profiles (SDP) of additional nucleosome occupancy datasets.** SDP were constructed as described for Figure 4 of the main article, but for additional nucleosome occupancy datasets. The left panels show main and the right panels show background clusters. S12A: tiling-array data in 5 bp resolution [40]; S12B: sequencing-based data in 1 bp resolution [65]; S12C: sequencing-based data in 1 bp resolution from cells grown on galactose [37].
(TIFF)

Figure S13 **Changes in nucleosome occupancy and transcription in the** ***mcm1-1*** **strain.** Same as Figure 6A of the main article but for all clusters.
(TIFF)

Figure S14 **Changes in nucleosome occupancy and transcription in the** ***tbf1*** **strain.** Same as Figure 6 of the main article but for all clusters and data from the *tbf1* strain.
(TIFF)

Figure S15 **Changes in nucleosome occupancy and transcription in the** ***cep3*** **strain.** Same as Figure 6 of the main article but for all clusters and data from the *cep3* strain.
(TIFF)

Figure S16 **Changes in nucleosome occupancy and transcription in the** ***abf1-101*** **strain.** Same as Figure 6 of the main article but for all clusters and data from the *abf1-101* strain.
(TIFF)

Figure S17 **Changes in nucleosome occupancy and transcription in the** ***rap1-1*** **strain.** Same as Figure 6 of the main article but for all clusters and data from the *rap1-1* strain.
(TIFF)

Figure S18 **Changes in nucleosome occupancy and transcription in the** ***rsc3-1*** **strain.** Same as Figure 5 of the main article but for all clusters.
(TIFF)

Figure S19 **Changes in nucleosome occupancy and transcription in the** ***reb1-212*** **strain.** Same as Figure 6 of the main article but for all clusters and data from the *reb1-212* strain.
(TIFF)

Figure S20 **Nucleotide content &** ***in vitro*** **nucleosome occupancy.** As Figure 8 of the main article but for background clusters.
(TIFF)

Table S1 **Strains and culture conditions used for the respiratory oscillation datasets.**
(PDF)

Table S2 **Cluster size, TSS fraction and phase angle density peaks.** Cluster size, TSS fraction and phase angle density peaks. Number of genes in each cluster, fraction of cluster genes for which TSS could be found (see Methods section “Transcription Start Sites”), circular density peaks of cluster gene phase angles, and peak time (time of experiment, with the first sample as origin time 0) in the first cycle, estimated from phase angle density peaks and the cycle periods (42 min and 300 min, respectively).
(PDF)

Table S3 **Significantly enriched GO terms of background clusters.** Functional analysis of background Clusters. Same as Table 1 of the main article (see there for abbreviations), but for background clusters. Results for all GO terms and clusters are provided as Dataset S2.
(PDF)

Table S4
**Significantly enriched metabolic subsystems of clusters.** Metabolic activities of clusters. Metabolic pathway or subsystem annotations for each gene were derived from a full-scale reconstruction of the metabolic network of baker’s yeast [59]. The “SUBSYSTEM” annotation was only available in the first version v1.0 of the network. Cumulative hypergeometric distribution tests were performed as described for GO analysis, and only significantly enriched subsystems are shown (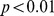). The number of genes (cluster/total) and p-values (“*p*”) for enrichment are given in brackets.(PDF)

Table S5
**Enriched transcription factor binding sites and motifs.** Enriched transcription factor binding sites and motifs. The presence of experimental protein binding sites (left) and DNA sequence motifs (right) in promoters and 3′UTRs were establishedd as described in the Methods section of the main article. Only significantly enriched sites/motifs (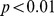 in cumulative hypergeometric distribution tests) are shown. The numbers in brackets show the number of genes in the cluster and the total number of genes with one or more occurrences of the given motif or site in the promoter region or downstream of 3′ends (indicated by suffix “.3p”). The full set of tested bindings sites and motifs are shown in Figure S3 and provided as Datasets S5 & S6.(PDF)

Table S6 **Data sources: URLs from which the original data was downloaded.** Data Sources. The URLs from which the analyzed data was originally downloaded. If the links are not active anymore, the data can be obtained from the authors on request.
(PDF)

Table S7 ***Saccharomyces cerevisiae*** **strains used in analyzed datasets.** Strain information for all datasets used in this study, derived from original publications.
(PDF)

Table S8 **Coordinate-based Data for SDP Plots.** This table maps y-axis labels of SDP plots to a data ID used in the underlying data collection. This collection is provided as a big archive file (295 MB) at http://www.tbi.univie.ac.at/raim/data/2011/yeast/clusters/geneData.tar.gz. Each file in the archive corresponds to one SDP. The rows are all genes for which a TSS could be defined (see Methods of the main paper), and the columns give values for each position from −1500 upstream to +1500 downstream of the TSS (+1). TSS were aligned to the genome in the SGD release from Feb. 2008.
The main results and underlying data of this paper are made available in CSV format (comma-separated values) at http://www.tbi.univie.ac.at/raim/data/2011/yeast/clusters/. In the following, the content of each file (column headers are in quotes) is described in detail:
(PDF)

Dataset S1
**Time Courses and Clusterings: tuliCoarse.results.csv.** This file contains for each protein-coding yeast gene in our reference genome release: • Yeast gene identifier (“ID”), “name” and SGD identifier (“SGD ID”);• The “Overlap Clustering” analyzed in this work;• The “Order” of the probe sets in the the data structure after parsing the microarray image files with the R affy package. This is required for reproduction of clustering with flowClust; • Raw time series data (identified by the names of the underlying. CEL image files); • Oscillation characteristics at the phenotypic cycle numbers 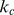, *i.e.*, 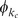 (“phase angle”), 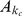 (“amplitude”), 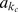 (“scaled amplitude”) and 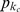 (“p-value” of periodicity); • Individual DFT-based clusterings of the two time series datasets (“clusters”); where column name prefixes “li06_” identify data based on the 0.7 h period dataset [11] and “tu05_” data based on the 5 h period dataset [10].(CSV)

Dataset S2 **GO Analysis: tuliCoarse.GO.results.csv.** A list of all 3,107 GO terms found in our reference genome annotation, including their definition (“description”), the “total” number of genes annotated with the respective term, the “number” of genes in all clusters, and the “p-value” for all clusters (from cumulative hypergeometric distribution tests, see Methods).
(CSV)

Dataset S3
**Meta-Transcriptome Analysis: tuliCoarse.transcriptome.results.csv.** A list of 1,327 transcriptome (microarray) experiments, including PubMed ID (“PMID”), a short experiment description (“Condition Name”), an experiment “index”, all exactly as provided by the original publication of this data collection [60], and the SOTA-based clustering used for column-sorting in Figure 3A of the main article (“SOTA cluster”), and for all clusters the scaled rank-sum 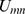 (“U/(m*n)”) and a “p-value” derived from two-sided Wilcoxon tests, comparing the distribution of cluster genes with the respective rest of the genome.(CSV)

Dataset S4 **IUPAC Motifs: iupac.motifs.csv.** A list of consensus DNA motifs in IUPAC format with an “ID”, as used in Table S5 and Figure S3A (see Methods section of the main article), the IUPAC “SEQUENCE PATTERN”, and a “DESCRIPTION”, including PubMed IDs of the original publications where the motifs were taken from.
(CSV)

Dataset S5 **Protein Binding Analysis: tuliCoarse.ChIP.results.csv.** A list of all 135 protein binding sites in promoter regions from experiments in [73] (“macisaac06.5.1” in column “SOURCE”) and [101] (“lieb01.rap_sir”) as used for Table S5 and Figure S3B. The column “total” gives the total number of genes in our reference genome annotation bound by the given protein as described in the Methods section of the main article, and columns “number” and “p-value” give the number of genes in the cluster and the p-value for enrichment in cumulative hypergeometric distribution tests.
(CSV)

Dataset S6 **Sequence Motif Analysis: tuliCoarse.motifs.results.csv.** A list of all 146 DNA motifs found in promoter regions. The motifs were either given as a position weight matrix [71] (“zhu09.pwms” in column “SOURCE”) or as consensus motifs in IUPAC motifs from diverse sources (“IUPAC.motifs”, see results file “iupac.motifs.csv” for definition and sources) as used for Table S5 and Figure S3A. The column “total” gives the total number of genes in our reference genome annotation harboring one or more instances of a given motif as described in the Methods section of the main article, and columns “number” and “p-value” give the number of genes in the cluster and the p-value for enrichment in cumulative hypergeometric distribution tests.
(CSV)

Dataset S7 **Categorical and Numerical Gene Data: gene.data.csv.** This file contains published data on yeast genes collected from various sources. The table below gives the column ID used, a short description and the source of the data set. Note, that Table 7 gives the URLs where the data were downloaded from. All original source data is also available from the authors on request.
(CSV)

Dataset S8 **ATP:ADP Measurement: atp_adp.results.csv.** Column “time, minutes” gives the experiment time in minutes, starting with 0’ at the first taken sample, column “dissolved O2, %” gives the measured dissolved oxygen concentration in percent of the saturation concentration, and column “ATP/ADP” gives the ratio, calculated as described in the Methods section of the main article.
(CSV)

Text S1 **Text S1 outlines problems with global microarray normalization and the choice of a “least-oscillating set” of genes as an alternative normalization reference (S1.1), the choice of DFT components for clustering (S1.2) and a general reasoning behind our clustering approach and the chosen algorithm (S1.3).**
(PDF)
