# Correction: The Yin and Yang of Yeast Transcription: Elements of a Global Feedback System between Metabolism and Chromatin

**DOI:** 10.1371/annotation/4a80d65a-35f9-4944-9254-4f8c566978f4

**Published:** 2012-06-15

**Authors:** Rainer Machné, Douglas B. Murray

Correction #1: Error in figure order.

 The images for Figures 7 and 8 were incorrectly switched. The image that appears as Figure 7 should be Figure 8, and the image that appears as Figure 8 should be Figure 7. The figure legends appear in the correct order.

Correction #2: Error in Figure 1 legend.

There is an error in the Figure 1 legend. The correct legend for Figure 1 is:

1A and 1B: overlaid time courses of summarized microarray fluorescence for each yeast gene, as the of the mean-ratio (), for the 0.7 h [11] and 5 h [10] period datasets, respectively. The bottom two panels show cluster averages for consensus and background clusters. The top panel shows the time courses of the dissolved O2 trace (DOT) in the culture medium in percent of the saturated concentration. Cluster colors and sizes (number of genes in each cluster) are given in the legend in Figure 1C. For clarity of visualization the time course data was normalized to a reference set that was selected for significant lack of oscillation (see Text S1 for fundamental problems with normalization of these datasets). Individual time courses for each cluster are plotted in Figure S2. 1D: phase-phase plot comparing the phase-angles of all transcripts in the two experiments. The phase angles were shifted such that cluster A phase angles are just above 0° in both datasets. Mapping back from frequency- to time-domain, we can locate the shifted phase angles of one cycle (0° and 360°) in the time series plot (vertical lines in Figures 1A and 1B), and use the same mapping in the top and right axes (in gray) of the phase-phase plot. The x- and y-extensions of each point scale with the transcript’s scaled amplitude in the respective dataset, where the non-consensus clusters (lower case letters) have a smaller initial size. Dataset S1 provides raw summarized microarray intensities, and the clustering of all analyzed yeast genes.

Correction #3: Errors in multiple URLs.

There are several links in this article that are incorrect. The correct versions of these links are:

The correct link found in the Genome Data Sources of the Discussion section is:

http://www.tbi.univie.ac.at/~raim/data/2011/yeast/clusters/geneData.tar.gz [^] [^]

The correct link found in the last paragraph of the Statistical DNA Profiles (SDP) section is:

http://www.tbi.univie.ac.at/~raim/data/2011/yeast/clusters/geneData.tar.gz [^] [^]

The correct links found in the Table S8 Supporting Information section are:

http://www.tbi.univie.ac.at/~raim/data/2011/yeast/clusters/geneData.tar.gz [^] [^]

and

http://www.tbi.univie.ac.at/~raim/data/2011/yeast/clusters/ [^] [^] 

