# Supplementary figures and images for: The Yin and Yang of Yeast Transcription: Elements of a Global Feedback System between Metabolism and Chromatin
Source: PLoS One. 2012 Jun 7;7(6):e37906. doi: 10.1371/journal.pone.0037906 (PMC3369881; doi:10.1371/journal.pone.0037906)

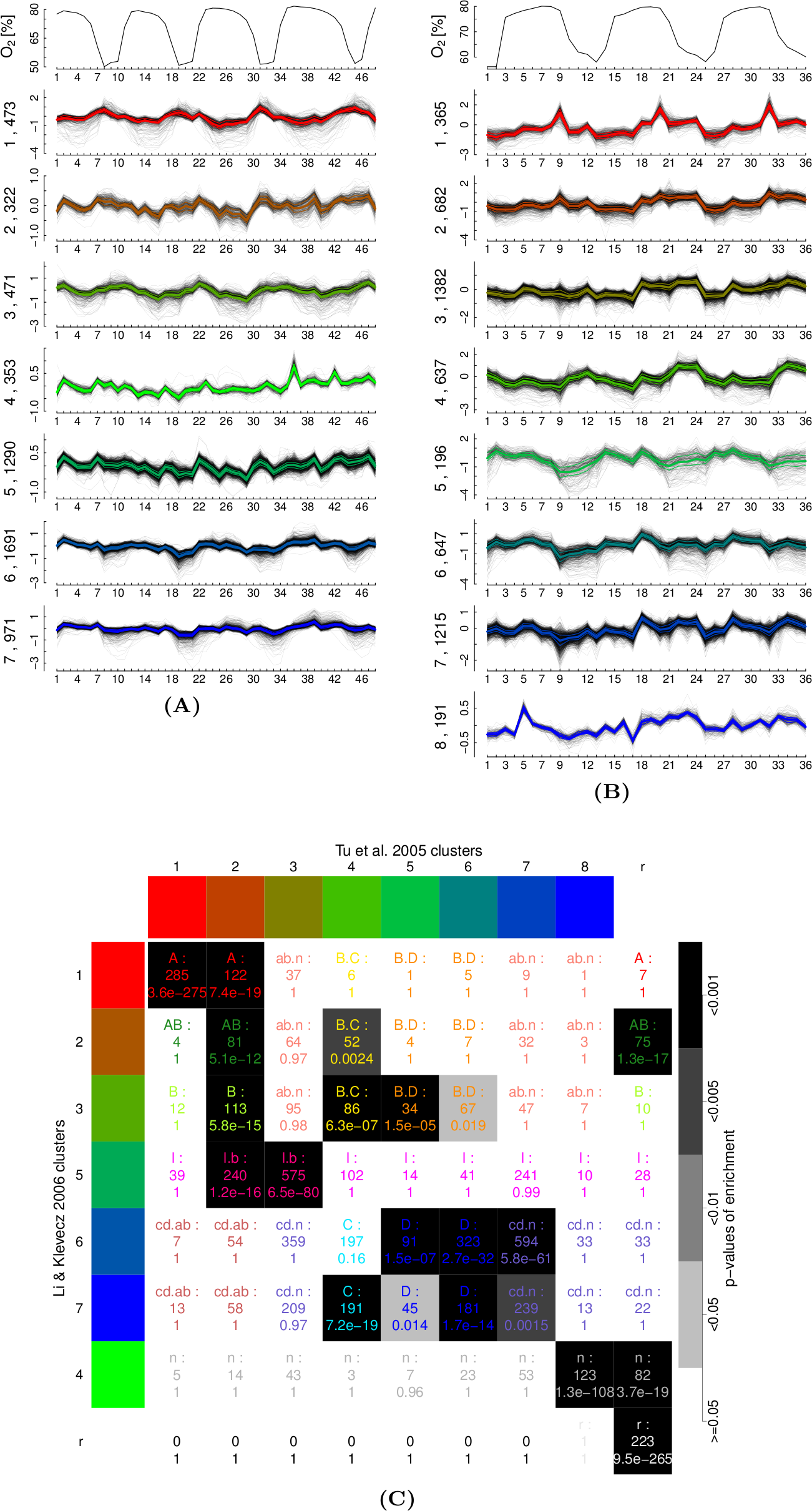

Supplement: Figure S1 — Overlap table of the two individual clusterings. 8 & 8: Individual flowClust clusterings of microarray fluorescence time series (shown is the log-ratio of raw data) from the 0.7 h (8) and 5 h (8) systems, after sorting by (phase-shifted) circular density peaks of the phase angles and re-labeling. The y-axis labels give the cluster assignments and the number of genes in each cluster. The thick and thin colored lines are the cluster mean and upper and lower quartiles, respectively, and gray lines are individual transcript time series. 8: Overlap table of the two individual sorted and re-labeled clusterings. For this plot, non-oscillatory clusters ( in of cluster genes) were additionally moved to the end, just before the not-on-array clusters “r”, i. e., cluster 4 in the 0.7 h system, while clusters 7 & 8 in the 5 h system did not require this step. The first row in each field gives the final cluster assignments used in this work, the middle row gives the number of genes in each field, and the bottom row gives the p-value from cumulative hypergeometric distribution tests. The p-values are additionally indicated by the gray-scale of the fields (see legend on the right axis). All clusterings are available in Dataset S1. (TIFF) [file pone.0037906.s001.tiff]

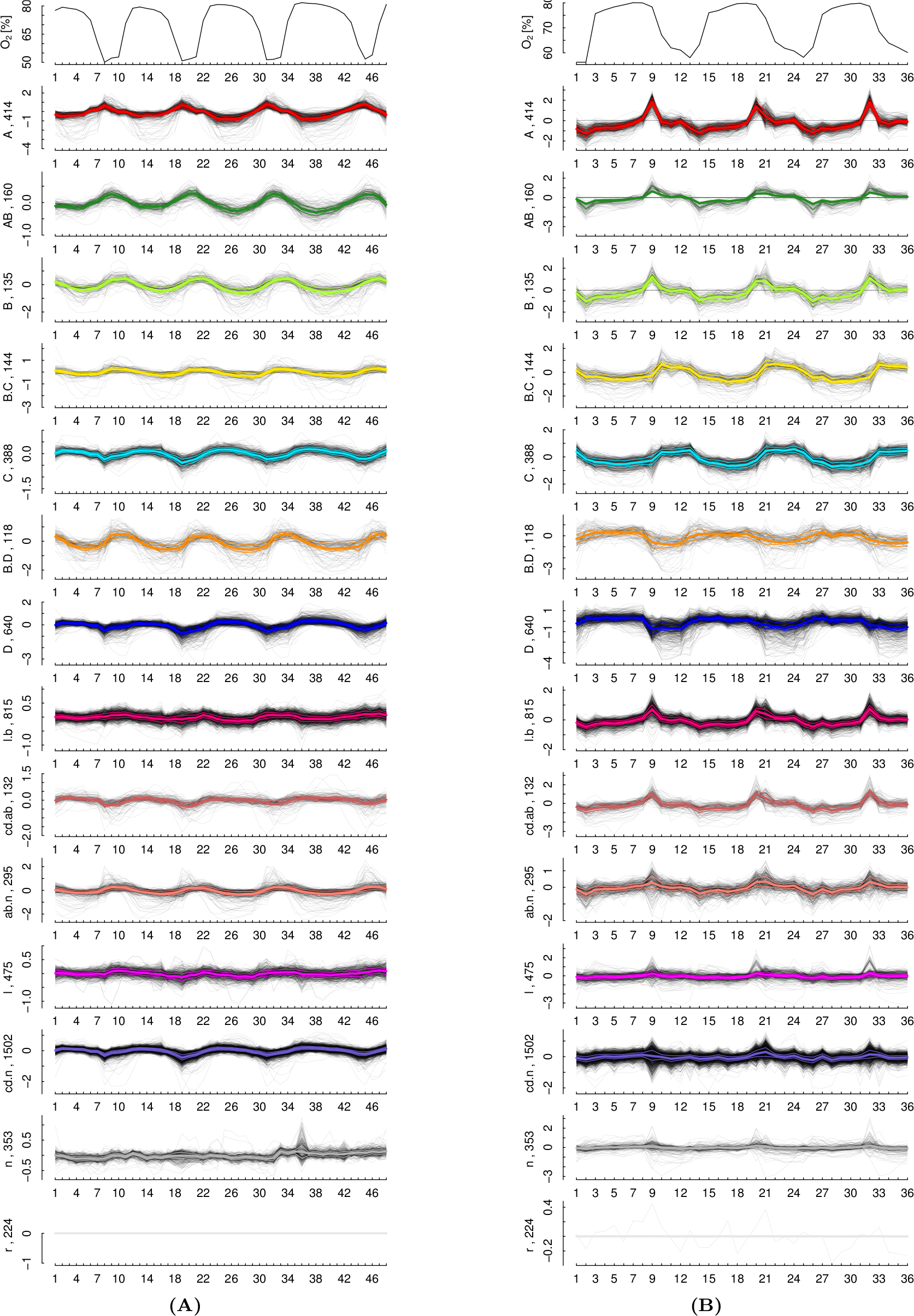

Supplement: Figure S2 — Normalized cluster time courses. Individual cluster time courses. Individual time courses of microarray fluorescence (as of the mean-ratio) of the final overlap clusters. The thick and thin colored lines are the cluster mean and upper and lower quantiles, respectively, and gray lines are individual transcript time series. S2A: 0.7 h period system [11] and S2B: 5 h period system [10]. Normalization was performed with “least-oscillating” gene sets as normalization reference, see Text S1 for details. The raw data is available in Dataset S1. (TIFF) [file pone.0037906.s002.tiff]

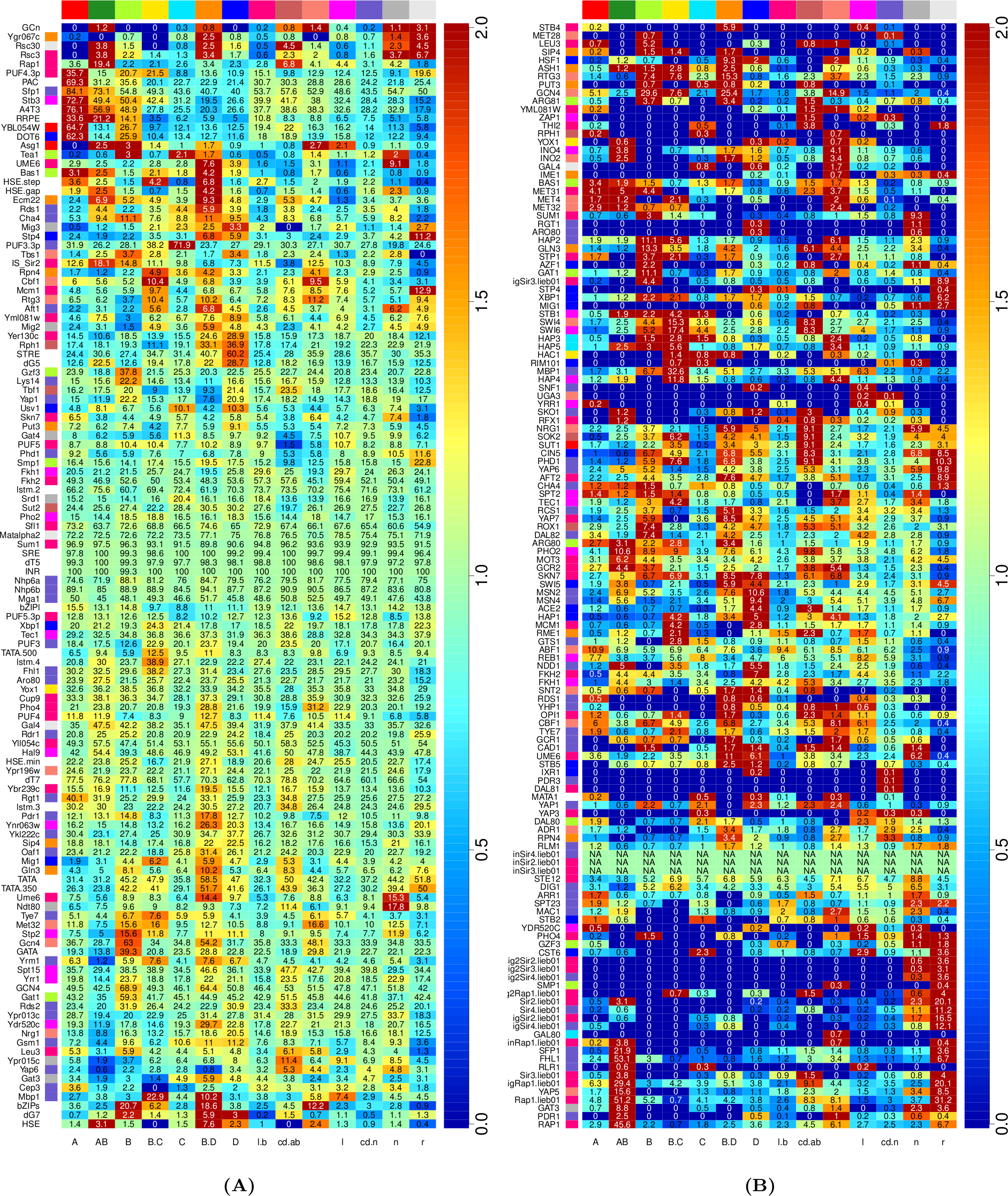

Supplement: Figure S3 — Transcription factor binding sites and motifs. Transcription factor motifs (10) and experimental binding sites (10), counts per cluster. Motifs and binding sites were obtained as described in the Methods section of the main article. Numbers give the percentage of cluster genes which have at least one occurrence of the given motif or protein binding (suffix “.3p” indicates occurrence downstream of the STOP codon). The enrichment E (see Methods) is color-coded, with a cut-off at . Rows were ordered by clustering the E values with hclust [105]. Table S5 lists all cluster motif/site combinations with a p-value in cumulative hypergeometric distribution tests and Datasets S5 & S6 provide all results. For binding sites and motifs associated with a specific proteins, the cluster assignments of the respective transcripts are shown as row colors. (TIFF) [file pone.0037906.s003.tiff]

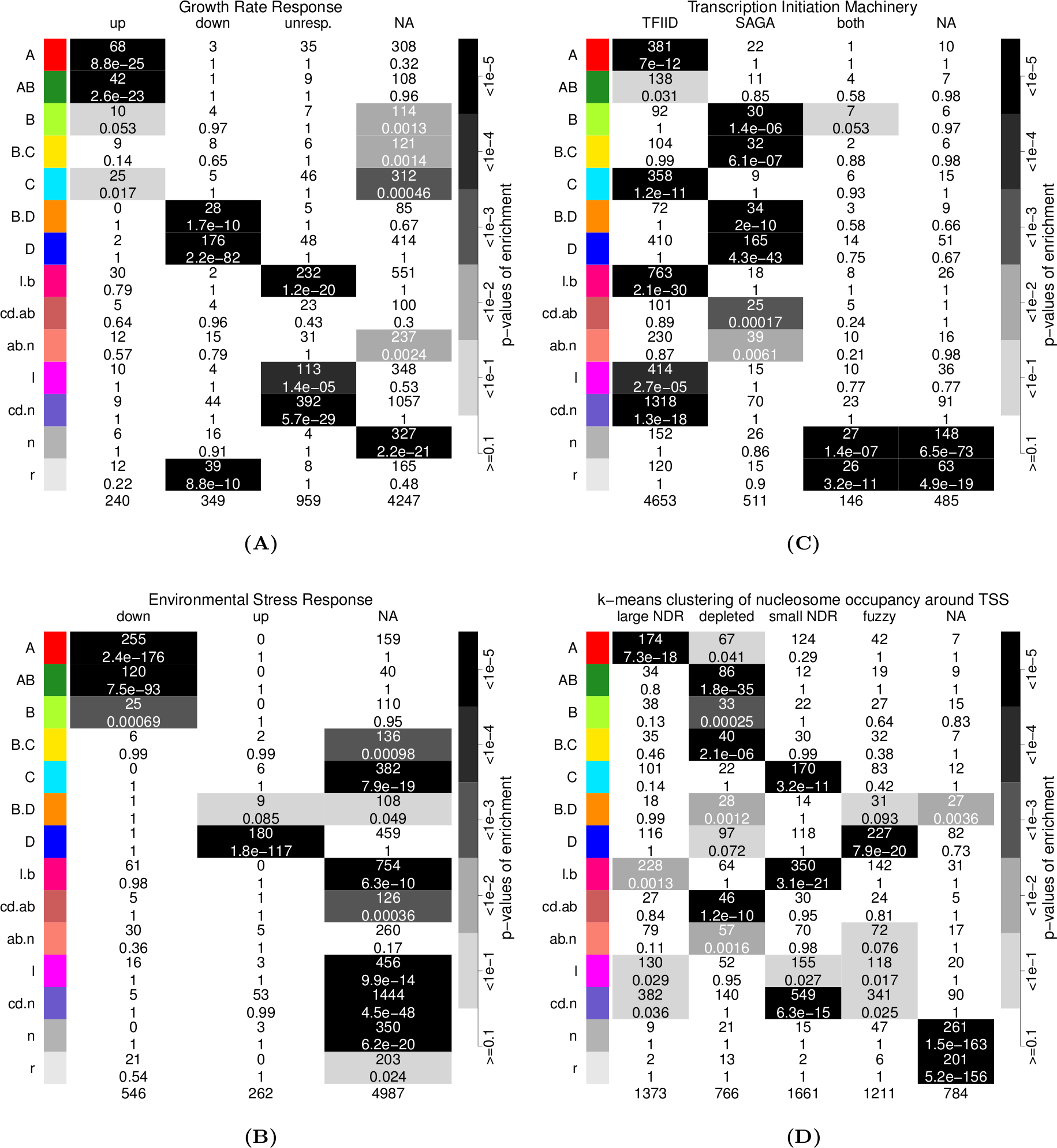

Supplement: Figure S4 — Overlap of the consensus clusters with promoter classes, and stress & growth rate response genes. As Figures 2A–2D of the main article, but for all clusters. All data are available in Dataset S7. (TIFF) [file pone.0037906.s004.tiff]

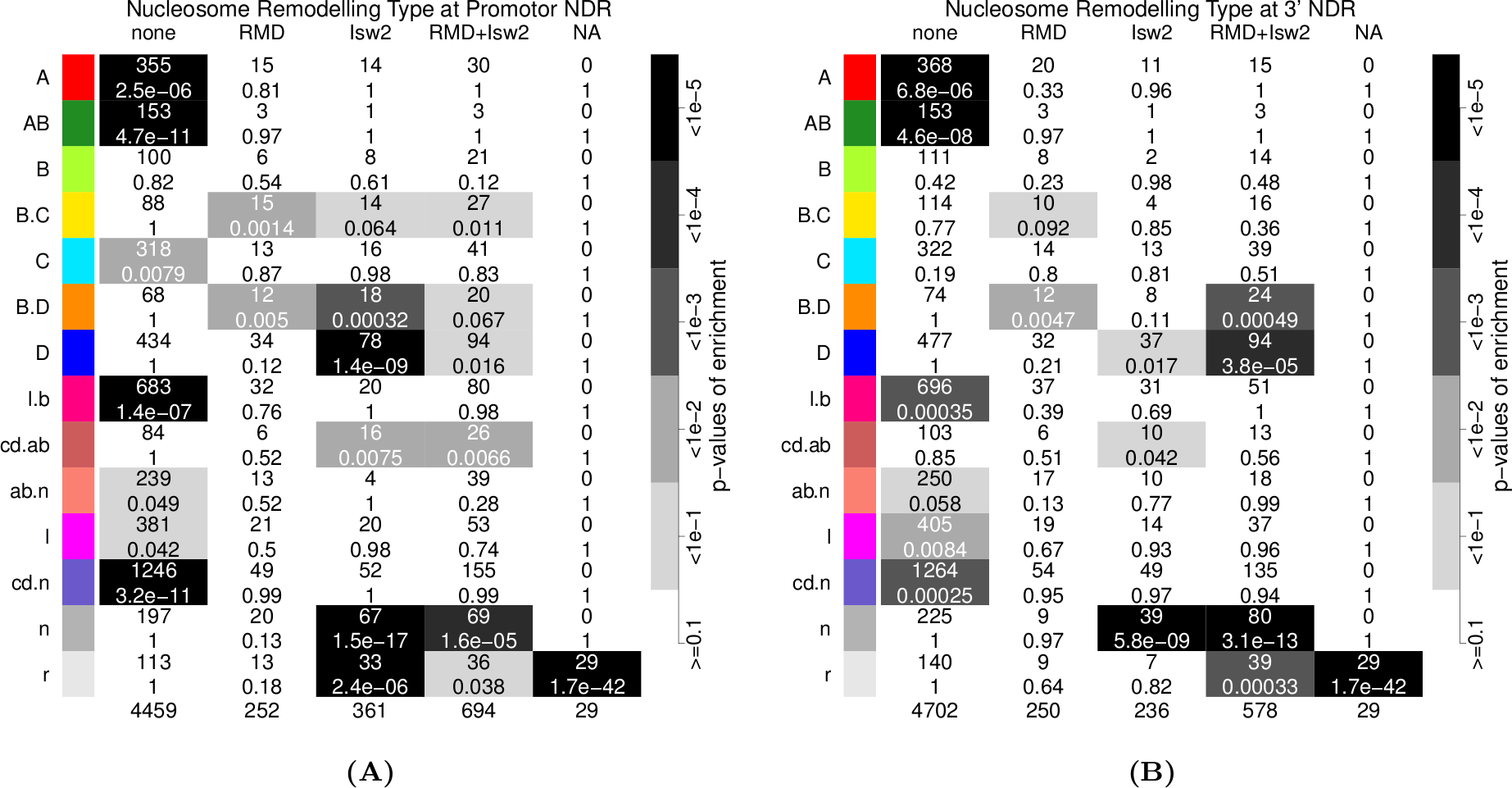

Supplement: Figure S5 — Isw2-bound and affected genes. As Figures 2E & 2F of the main article but for all clusters. All data are available in Dataset S7. (TIFF) [file pone.0037906.s005.tiff]

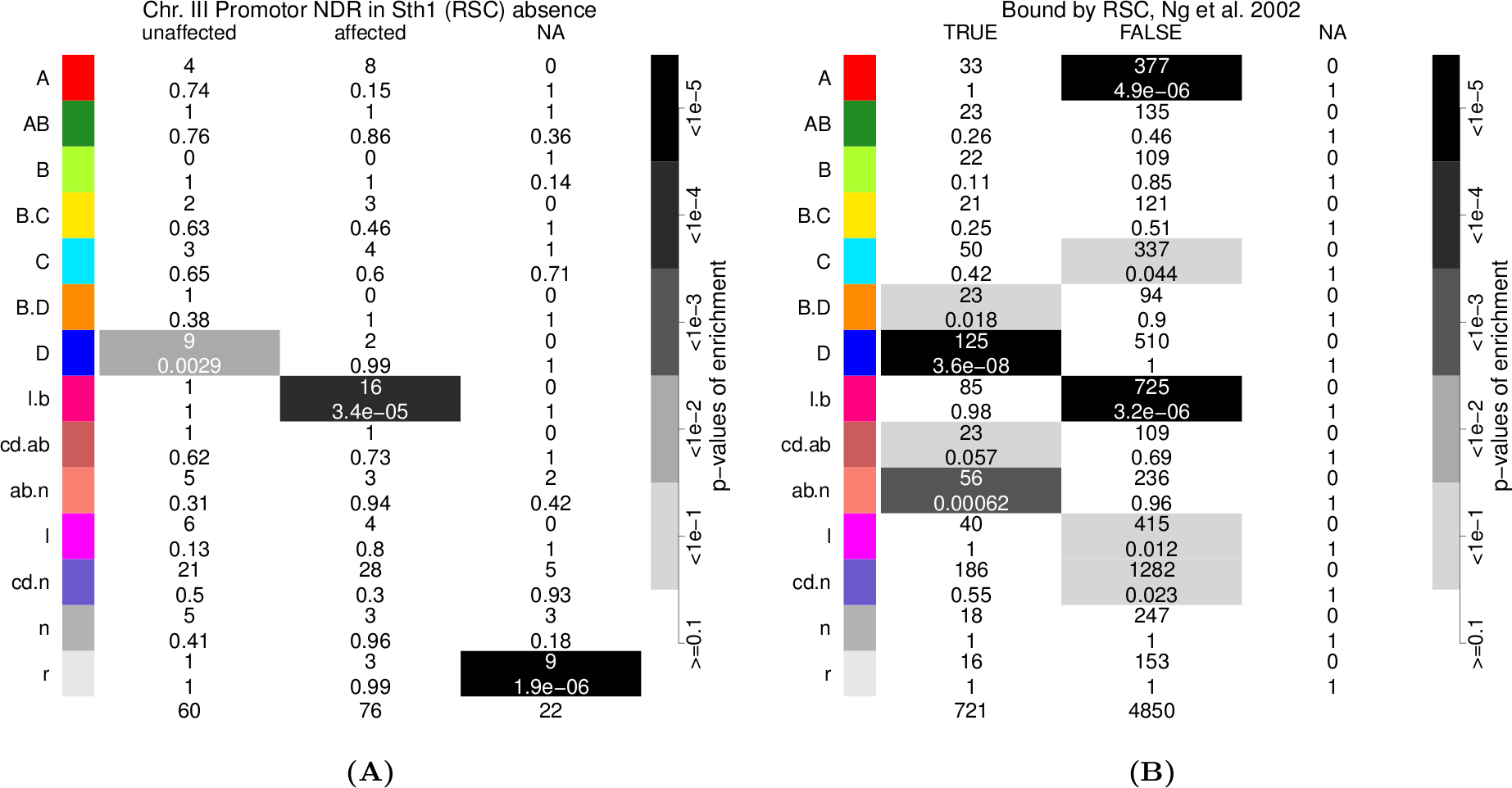

Supplement: Figure S6 — RSC-bound and -affected promoter classes. S6A: promoters on chromosome III were “affected” or “unaffected” (or not analyzed, “NA”) upon inactivation (by induced intein-splicing) of Sth1, the catalytic component of the RSC complex, from [42]. S6B: genes bound by the RSC complex defined via a “combined p-value” calculated from several complex components in [69], “TRUE”: and “FALSE”: . All data are available in Dataset S7. (TIFF) [file pone.0037906.s006.tiff]

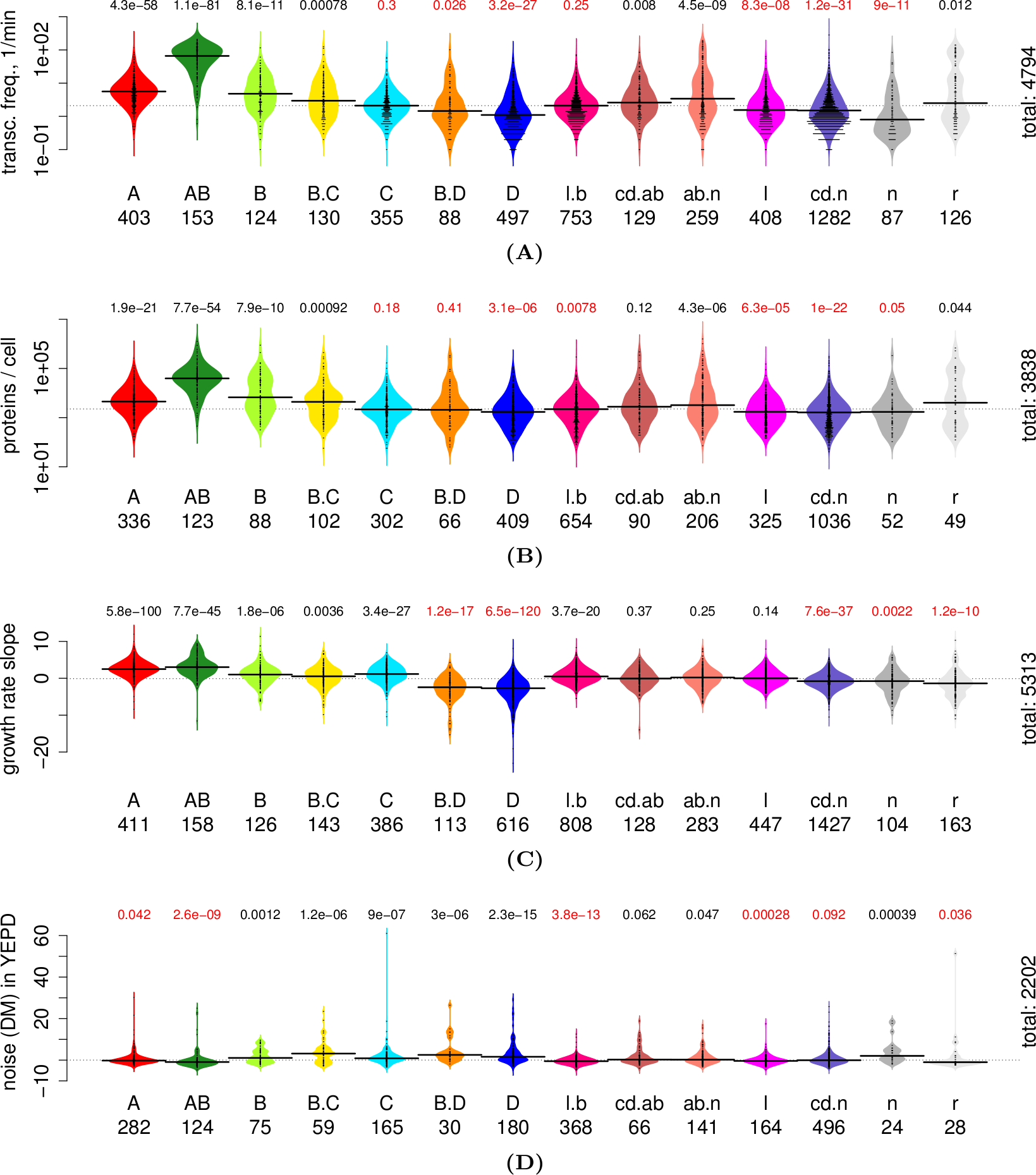

Supplement: Figure S7 — Transcriptional frequency, noise & growth-rate. Statistical biases that distinguish anabolic from catabolic superclusters. Cluster distributions are shown as bean-plots [106]. S7A: transcriptional frequencies, data from [107]; S7B: numbers of proteins per cell, data from [108]; S7D: transcriptional noise, data from [61]; S7C: correlation of expression with growth rates in nutrient-limiting conditions, data from [31]. Two-sided Wilcoxon rank-sum tests were applied to compare the distribution of n values in each cluster to the m values of all other genes. The number of cluster genes (n) for which a value was available in the given dataset is shown in the bottom row, and the total number of available values (m + n) is shown on the right y-axis. The dotted and solid lines show the total and cluster medians, respectively. The resulting p-values are shown above each plot and the text color indicates whether the cluster distribution is higher (black) or lower (red) then the distribution of the respective rest of the genome. All data are available in Dataset S7. (TIFF) [file pone.0037906.s007.tiff]

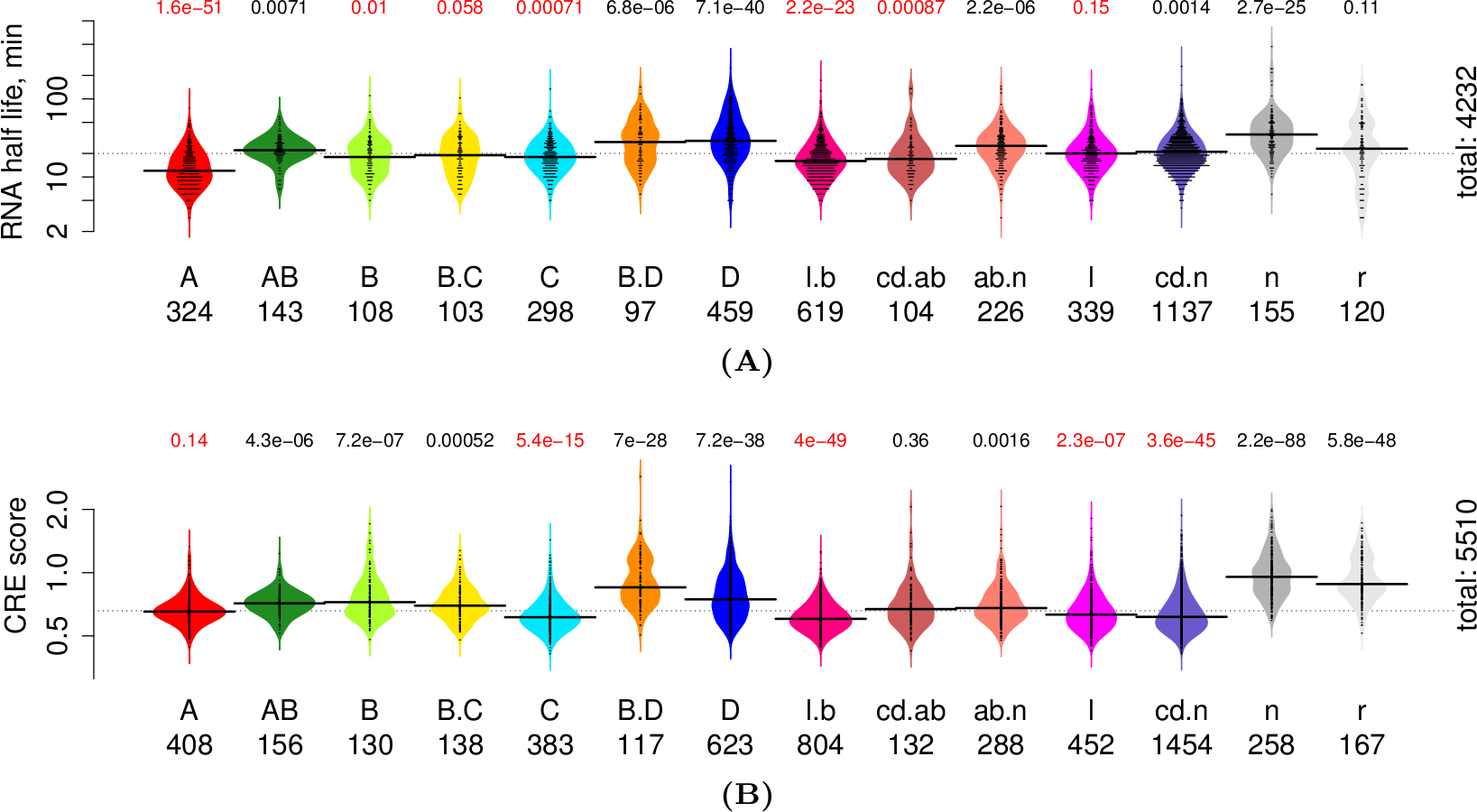

Supplement: Figure S8 — mRNA half-lives and Chromatin Regulation Scores. Statistical biases that distinguish ribosomal from metabolic superclusters. Same as Fig. S7 but for S8A: RNA half-lives, data from [109]; and S8B: chromatin-regulation score (CRE), data from [64]. Axis annotations as described for Fig. S7. All data are available in Dataset S7. (TIFF) [file pone.0037906.s008.tiff]

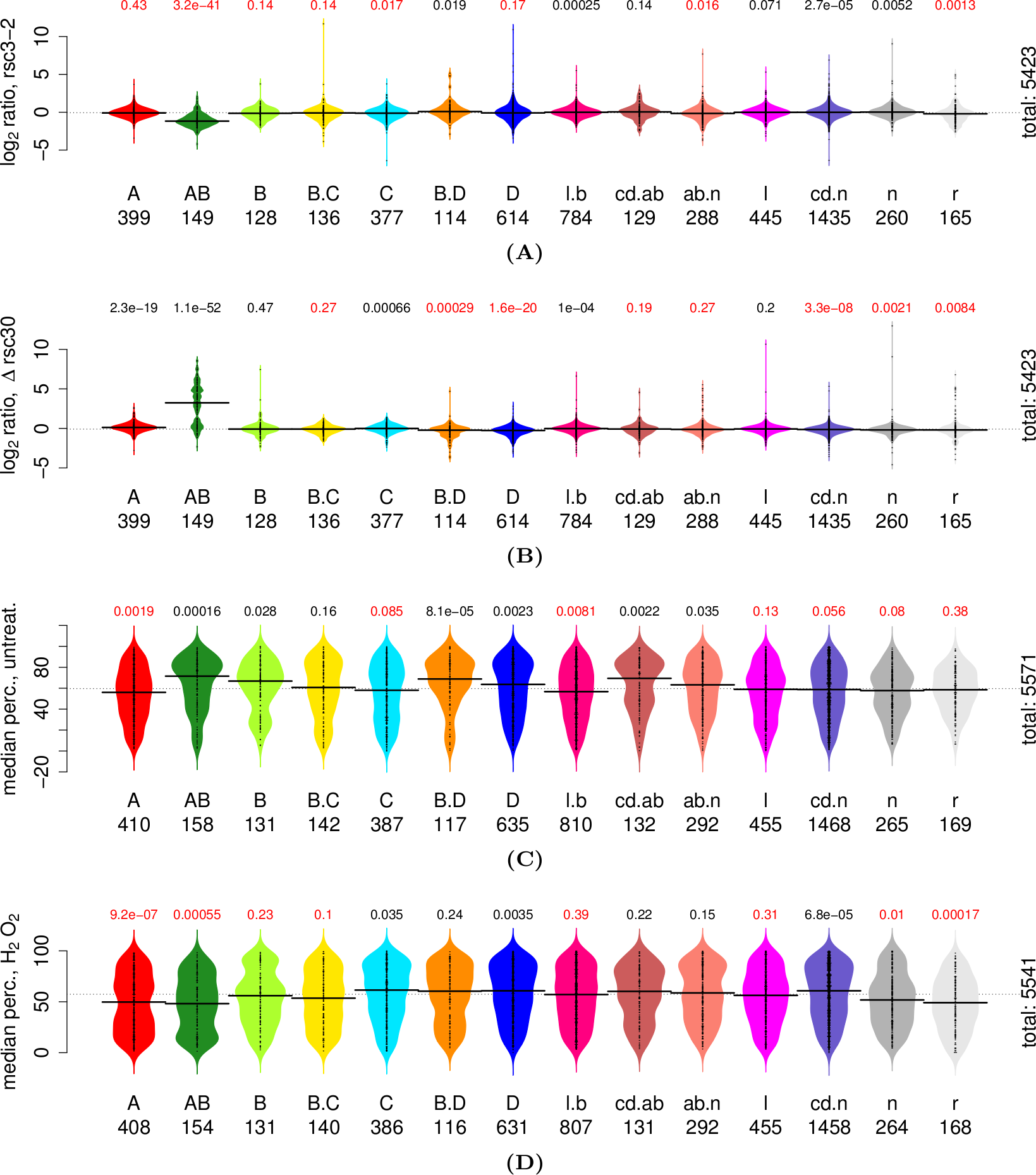

Supplement: Figure S9 — Expression in rsc3-2 and rsc30 strains and Rsc9p location. Change of transcript levels in strains carrying the rsc3-1 3-2 (16) and rsc30 (16) mutations; data from [70]. Rsc9p binding in untreated (16 and H2O2-treated cells, from [72]. Axis annotations as described for Fig. S7. All data are available in Dataset S7. (TIFF) [file pone.0037906.s009.tiff]

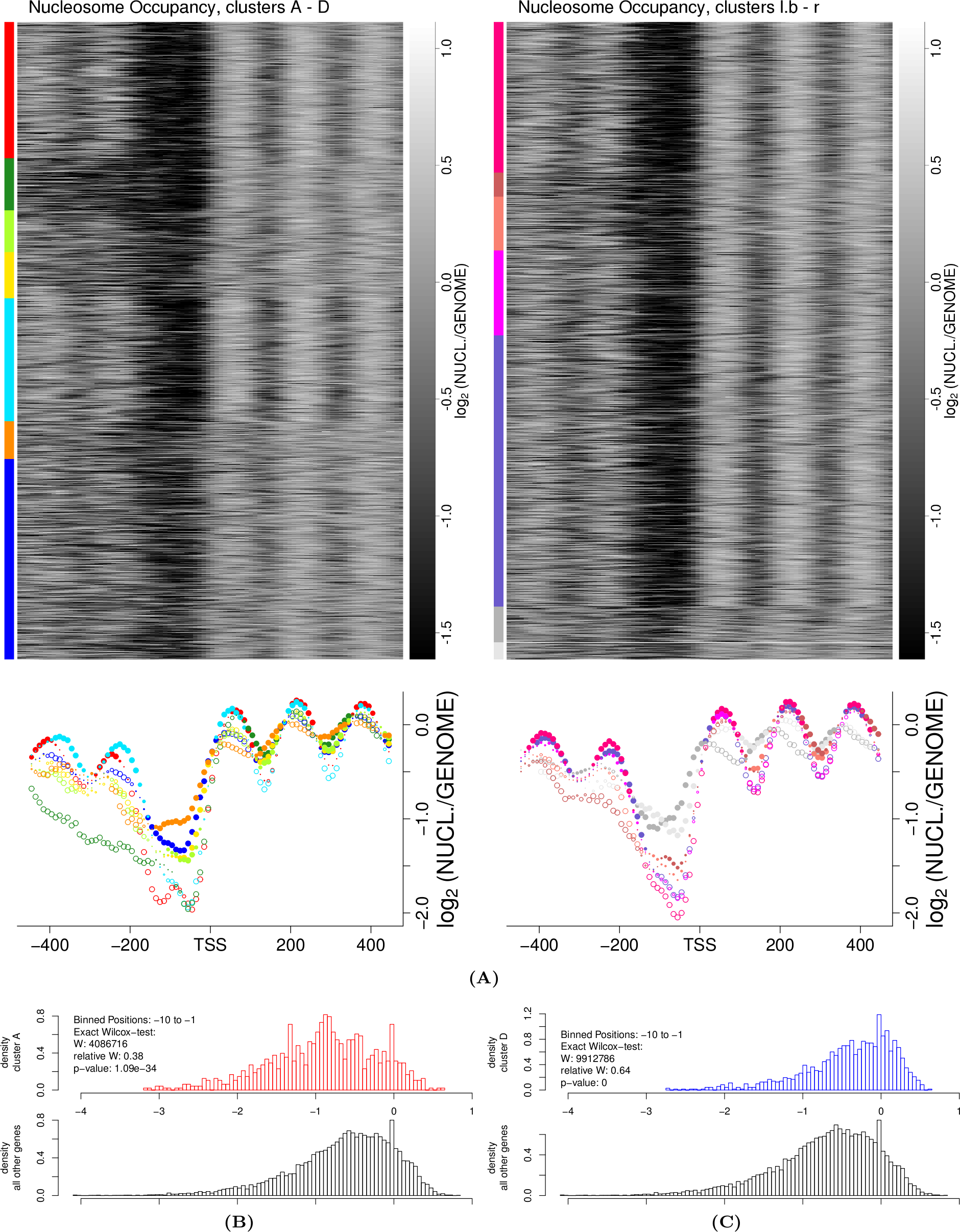

Supplement: Figure S10 — Nucleosome Occupancy: Heatmap and SDP construction. S10A: as Figure 4 of the main article, but for all clusters. Figures S10B and S10C show distrubtions and test results for the bin between positions -10 and -1 (from the TSS) for clusters A and D, respectively. The “relative W” value corresponds to . (TIFF) [file pone.0037906.s010.tiff]

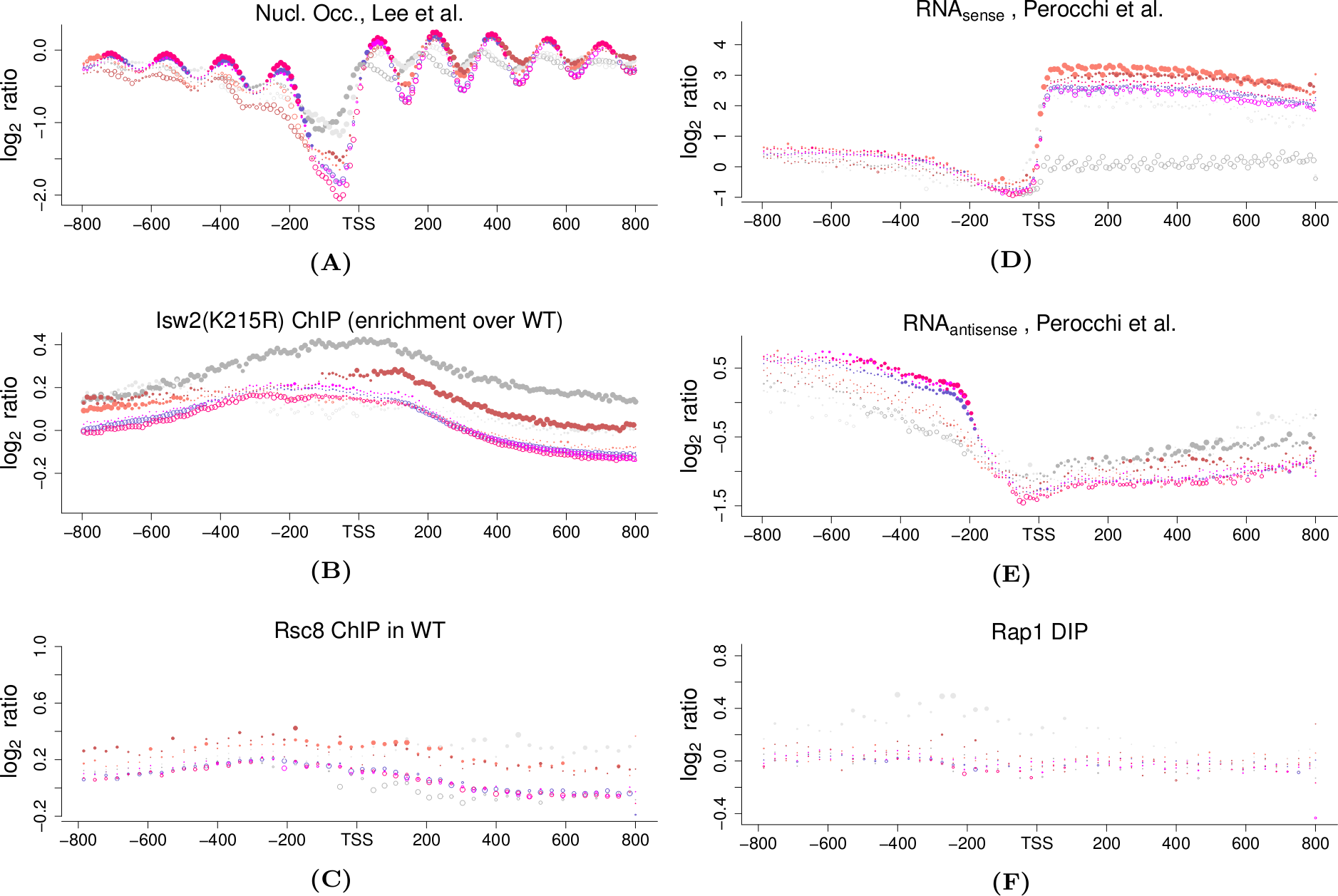

Supplement: Figure S11 — Statistical DNA profiles (SDP) of nucleosome occupancy, Isw2(K215R) ChIP, Rap1p DIP, Rsc8p ChIP & transcriptome tiling array datasets. Same as Figure 5 of the main article, but for background clusters. (TIFF) [file pone.0037906.s011.tiff]

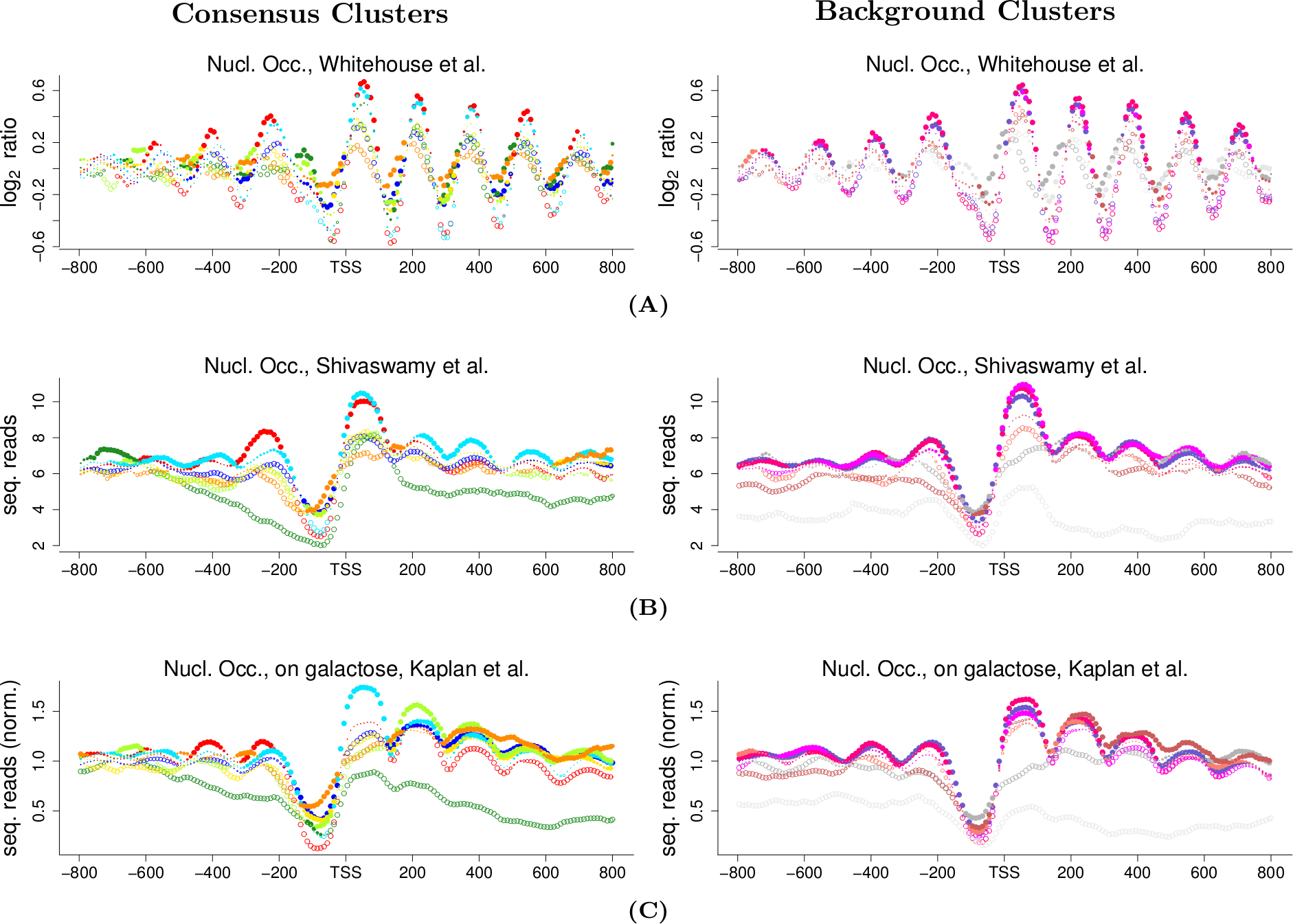

Supplement: Figure S12 — Statistical DNA profiles (SDP) of additional nucleosome occupancy datasets. SDP were constructed as described for Figure 4 of the main article, but for additional nucleosome occupancy datasets. The left panels show main and the right panels show background clusters. S12A: tiling-array data in 5 bp resolution [40]; S12B: sequencing-based data in 1 bp resolution [65]; S12C: sequencing-based data in 1 bp resolution from cells grown on galactose [37]. (TIFF) [file pone.0037906.s012.tiff]

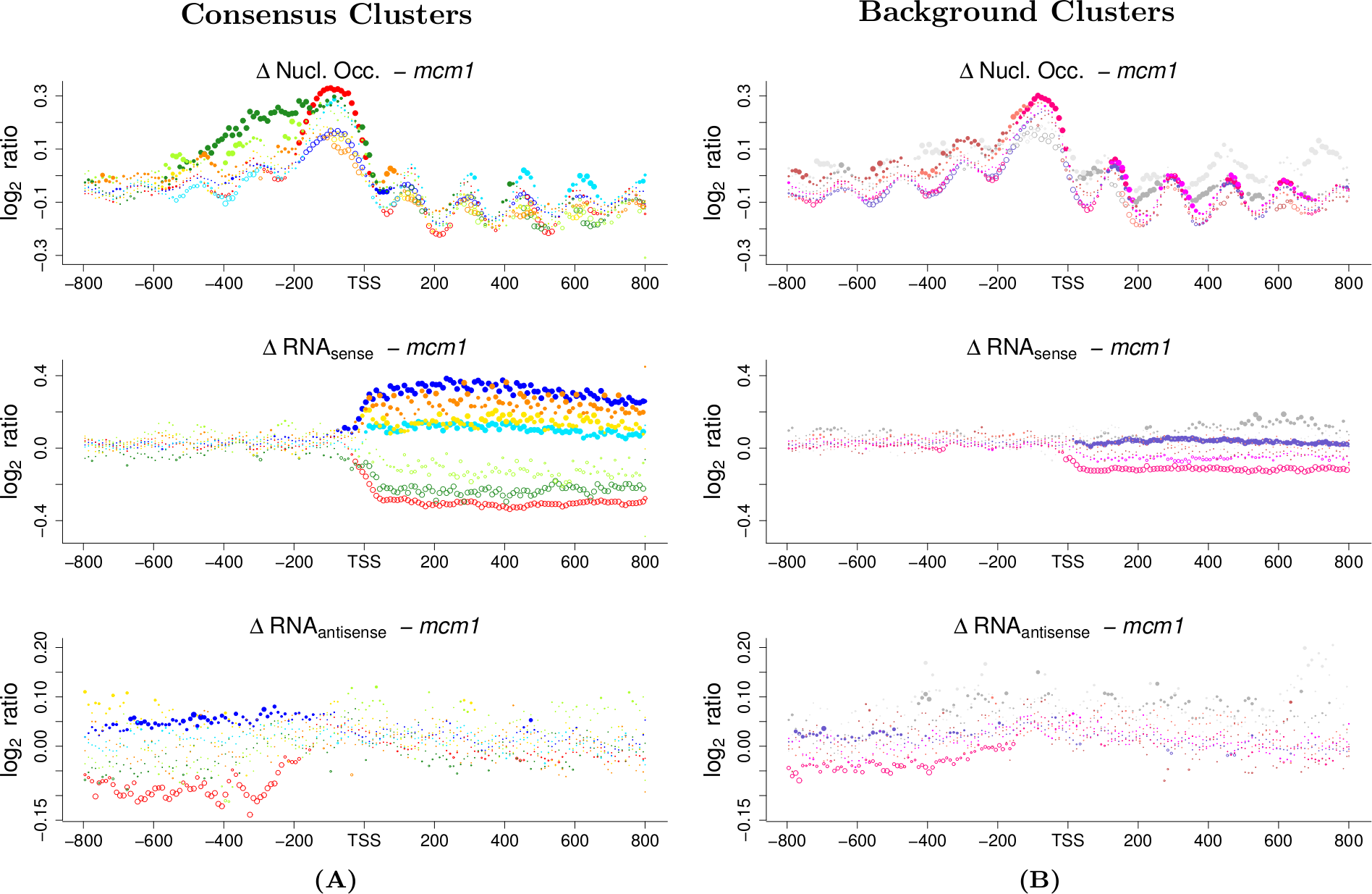

Supplement: Figure S13 — Changes in nucleosome occupancy and transcription in the mcm1-1 strain. Same as Figure 6A of the main article but for all clusters. (TIFF) [file pone.0037906.s013.tiff]

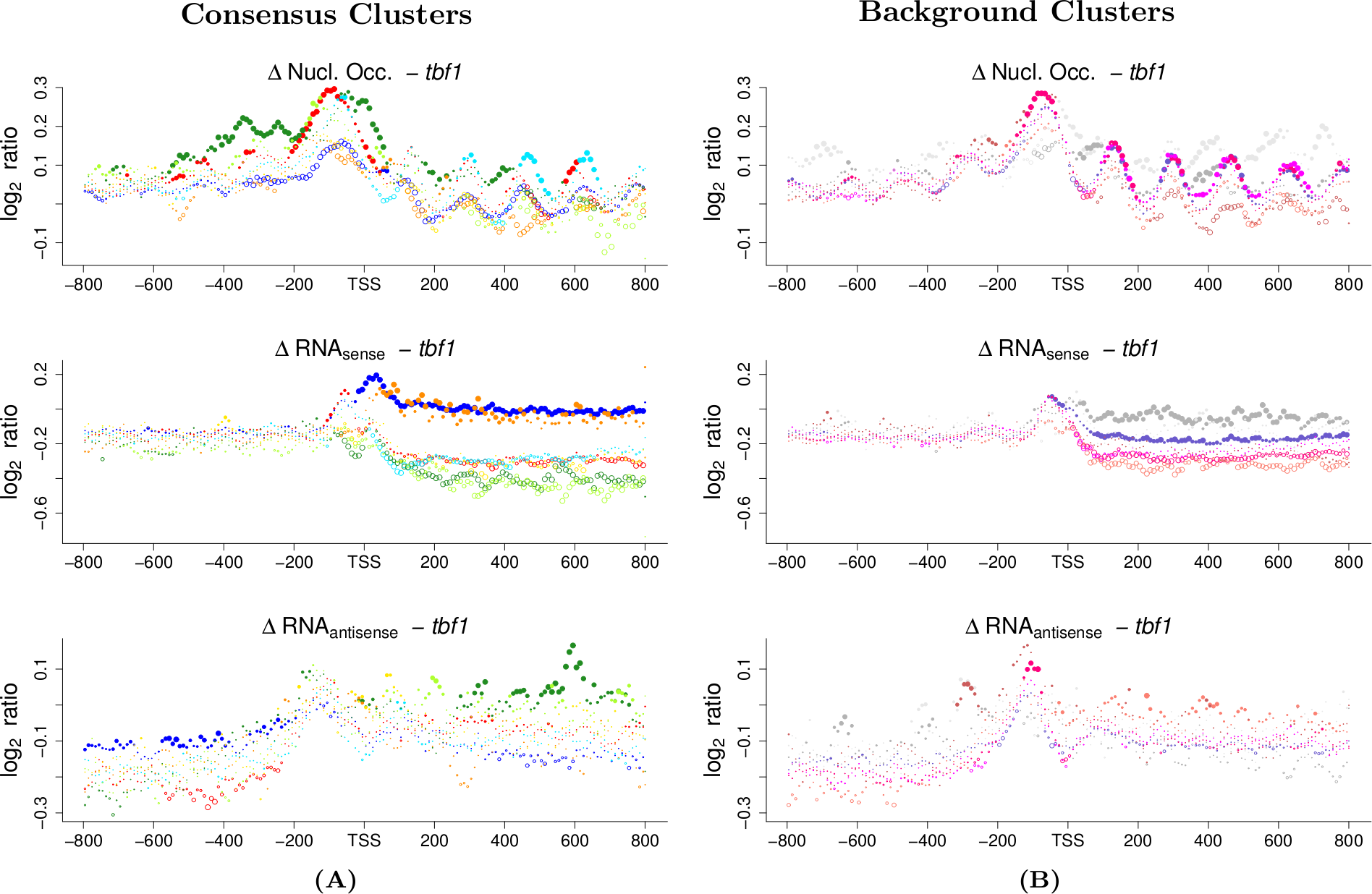

Supplement: Figure S14 — Changes in nucleosome occupancy and transcription in the tbf1 strain. Same as Figure 6 of the main article but for all clusters and data from the tbf1 strain. (TIFF) [file pone.0037906.s014.tiff]

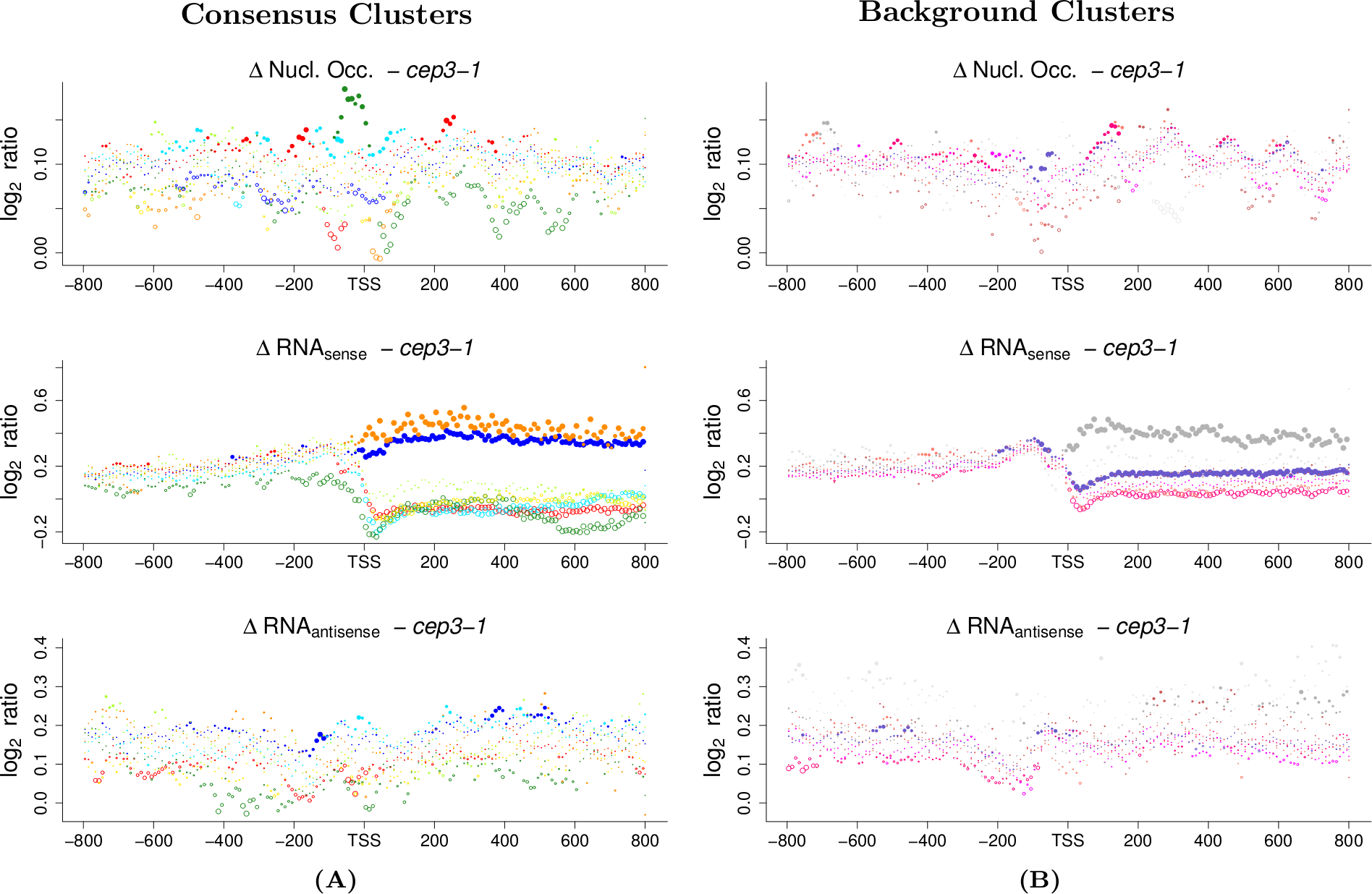

Supplement: Figure S15 — Changes in nucleosome occupancy and transcription in the cep3 strain. Same as Figure 6 of the main article but for all clusters and data from the cep3 strain. (TIFF) [file pone.0037906.s015.tiff]

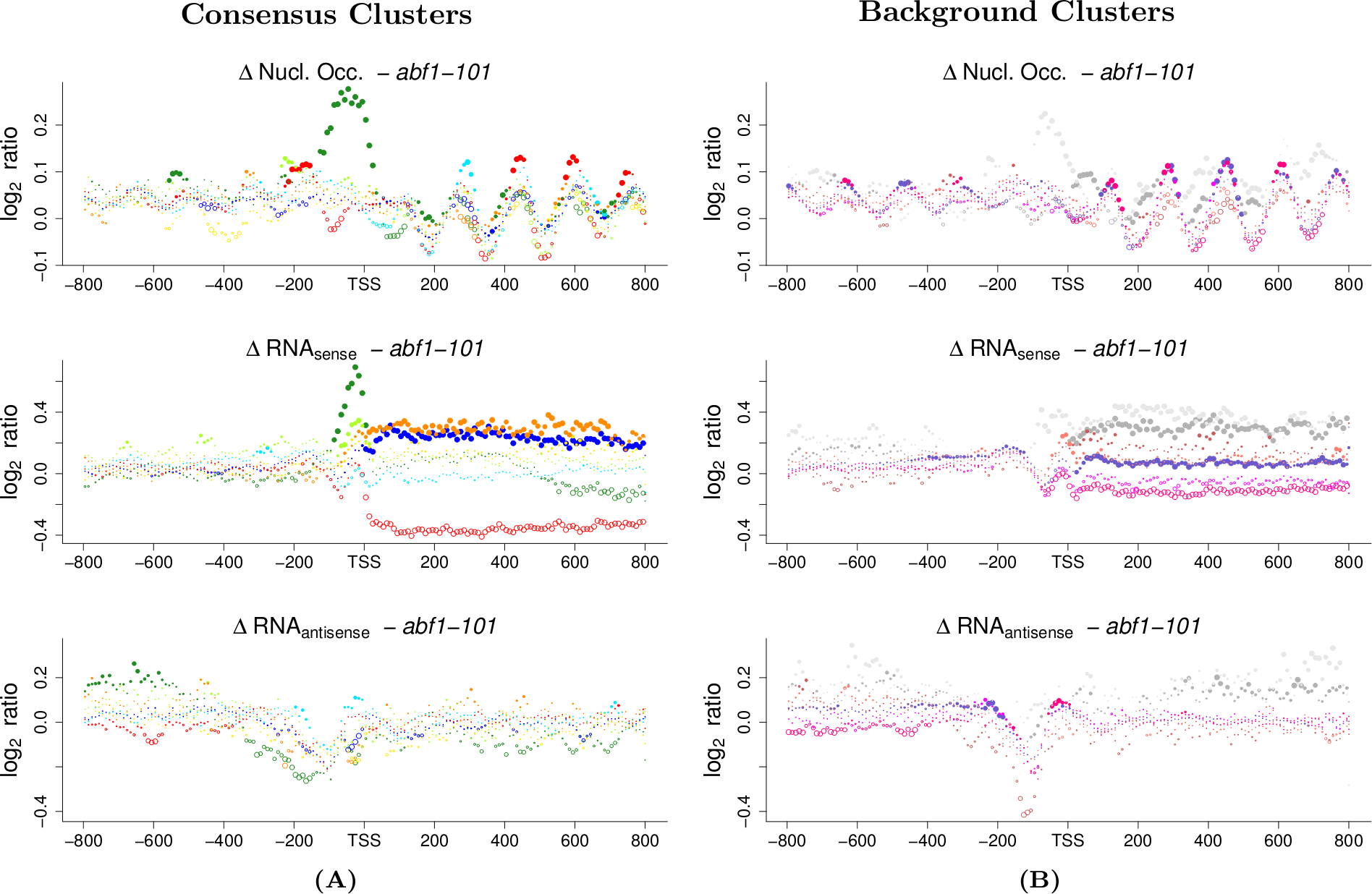

Supplement: Figure S16 — Changes in nucleosome occupancy and transcription in the abf1-101 strain. Same as Figure 6 of the main article but for all clusters and data from the abf1-101 strain. (TIFF) [file pone.0037906.s016.tiff]

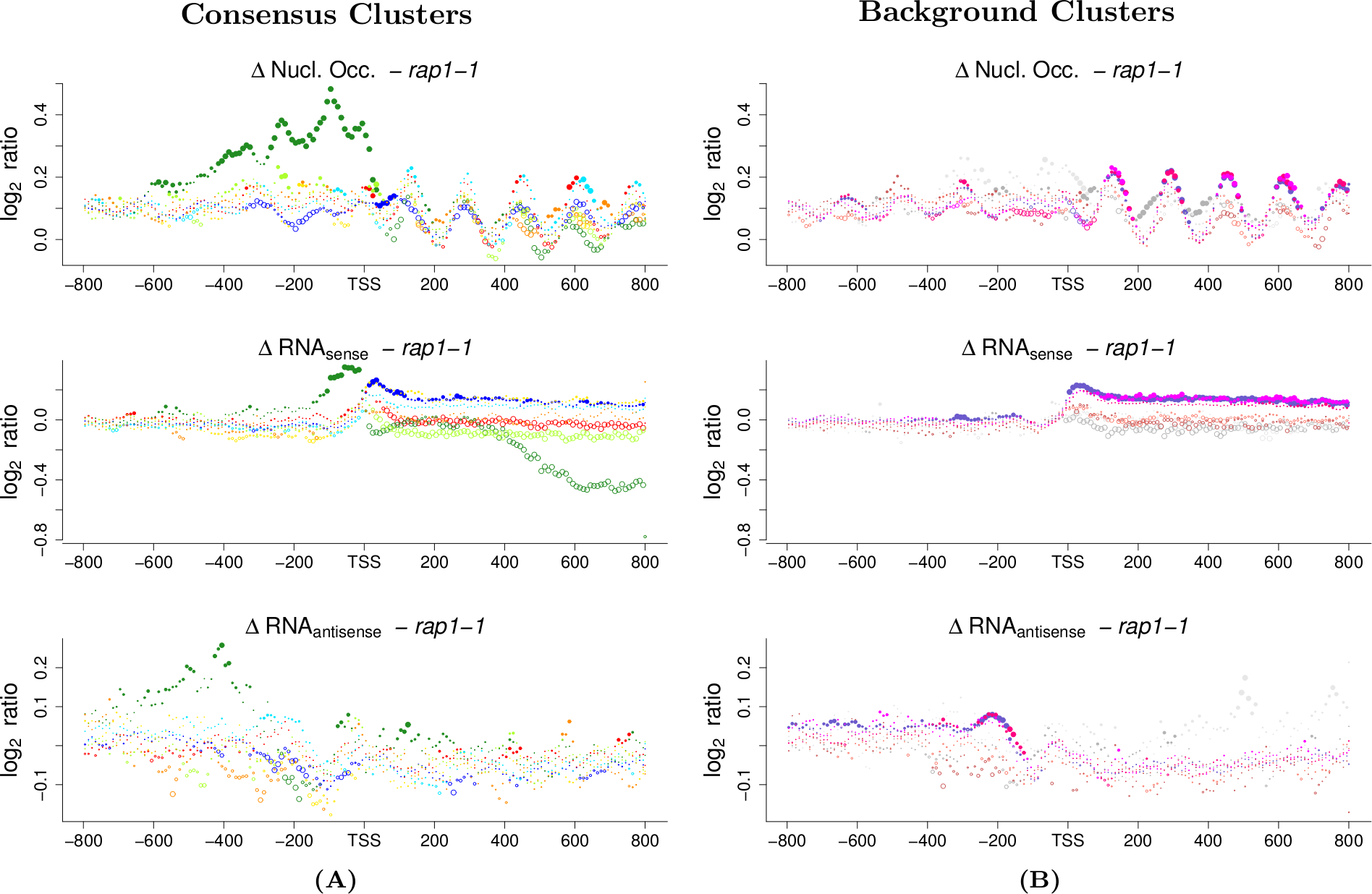

Supplement: Figure S17 — Changes in nucleosome occupancy and transcription in the rap1-1 strain. Same as Figure 6 of the main article but for all clusters and data from the rap1-1 strain. (TIFF) [file pone.0037906.s017.tiff]

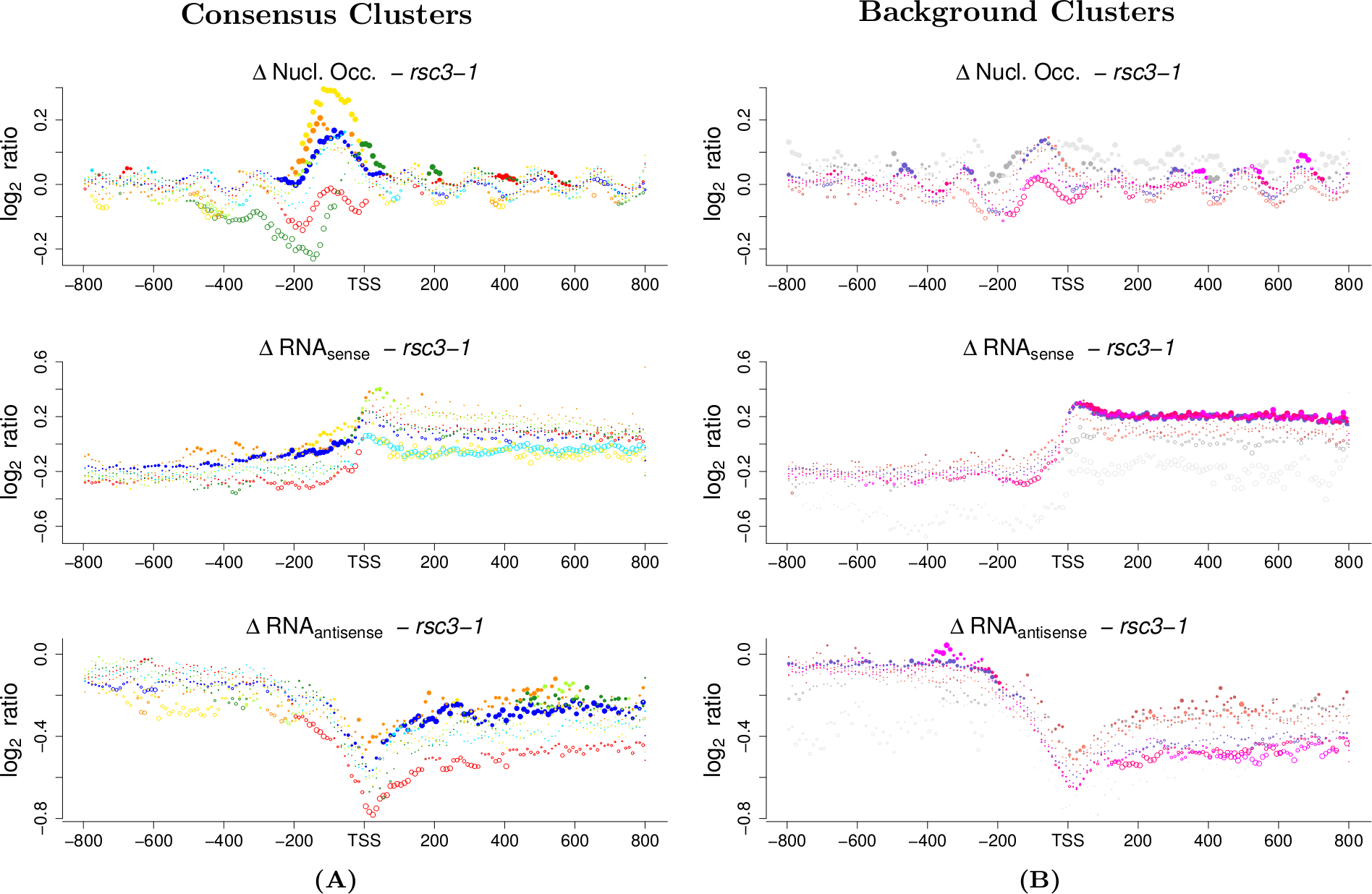

Supplement: Figure S18 — Changes in nucleosome occupancy and transcription in the rsc3-1 strain. Same as Figure 5 of the main article but for all clusters. (TIFF) [file pone.0037906.s018.tiff]

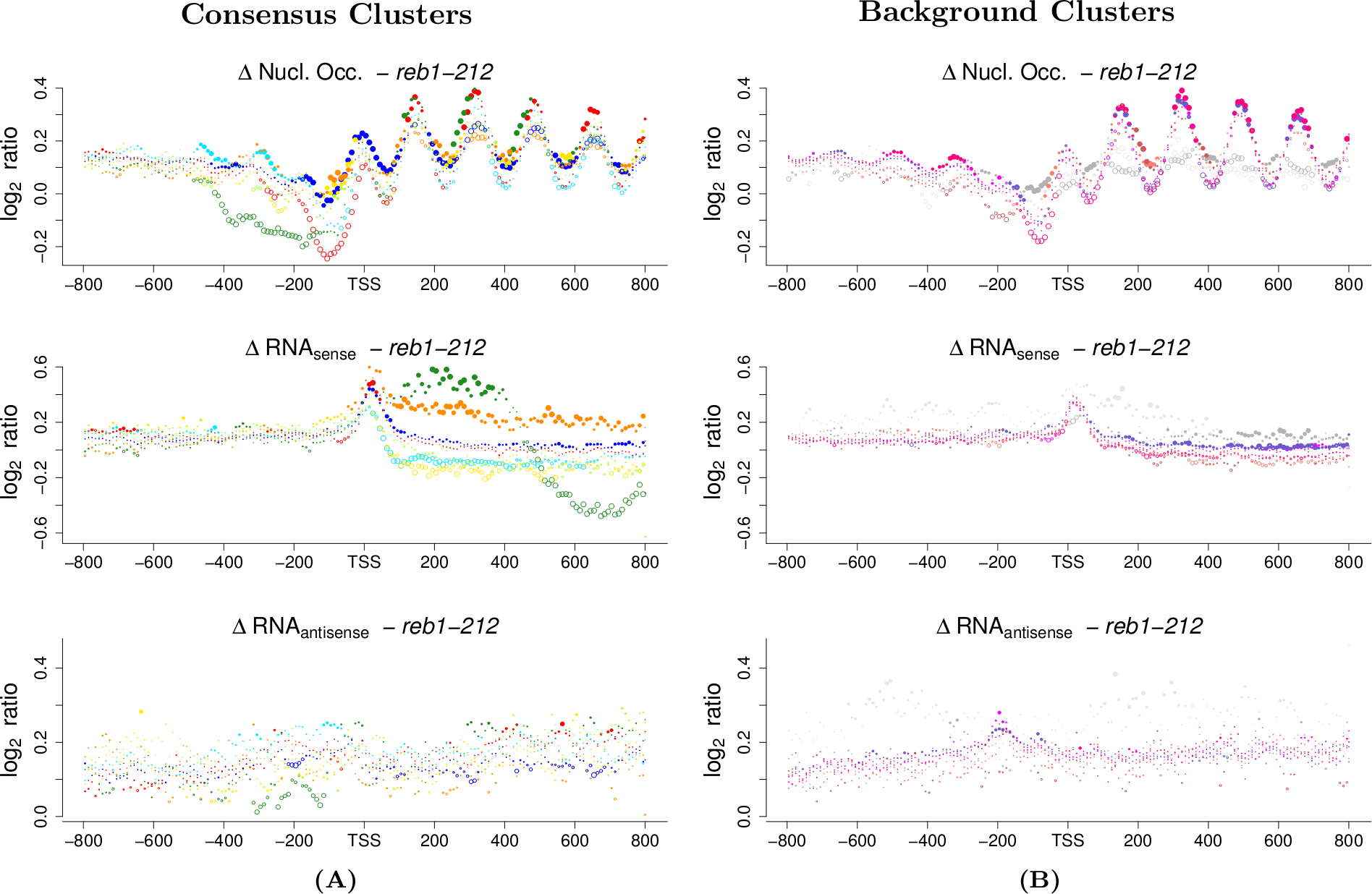

Supplement: Figure S19 — Changes in nucleosome occupancy and transcription in the reb1-212 strain. Same as Figure 6 of the main article but for all clusters and data from the reb1-212 strain. (TIFF) [file pone.0037906.s019.tiff]

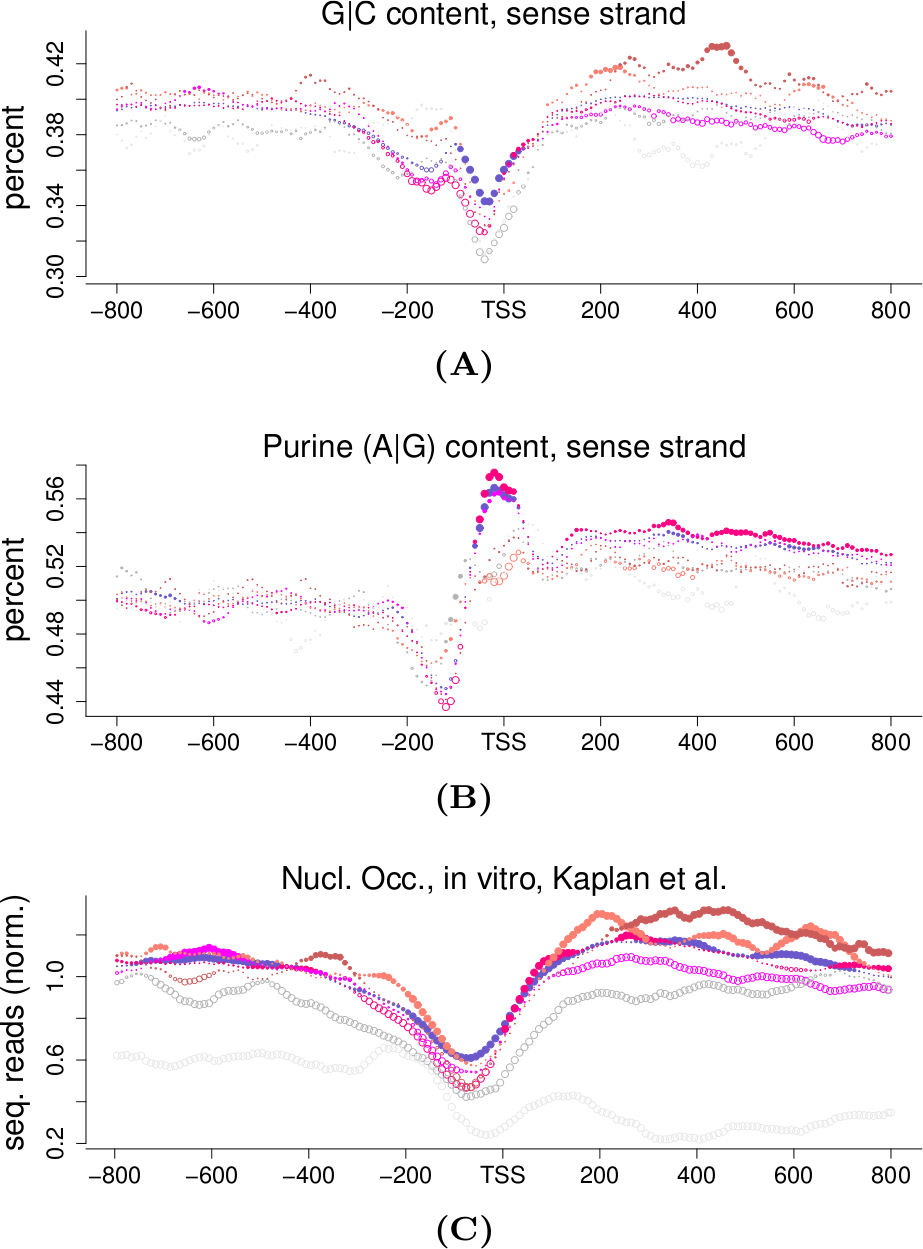

Supplement: Figure S20 — Nucleotide content & in vitro nucleosome occupancy. As Figure 8 of the main article but for background clusters. (TIFF) [file pone.0037906.s020.tiff]
