# Supplementary material for: The Yin and Yang of Yeast Transcription: Elements of a Global Feedback System between Metabolism and Chromatin
Source: PLoS One. 2012 Jun 7;7(6):e37906. doi: 10.1371/journal.pone.0037906 (PMC3369881; doi:10.1371/journal.pone.0037906)
Supplement: Table S1 — Strains and culture conditions used for the respiratory oscillation datasets. (PDF) [file pone.0037906.s021.pdf]

Supporting Table S1. Strains and culture conditions used for the respiratory oscillation datasets.

	**Li and Klevecz 2006**	**Tu et al. 2005**
**STRAIN**	IFO 0233	CEN.PK122
**MEDIA (per 1 L):**		
$(NH_4)_2SO_4$	5 g	5 g
KH_2PO_4	2 g	2 g
$MgSO_4.7H_2O$	0.5 g	0.5 g
$CaCl_2.2H_2O$	0.1 g	0.1 g
$FeSO_4.7H_2O$	0.02 g	0.02 g
$ZnSO_4.7H_2O$	0.01 g	0.01 g
$CuSO_4.5H_2O$	0.005 g	0.005 g
$MnCl_2.4H_2O$	0.001 g	0.001 g
antifoam A/204	(A) 0.2 mL	(204) 0.5 mL
Yeast extract	1 g	1 g
70% H_2SO_4	1 mL	0.5 mL
Glucose.1H_2O	19.25 g	10 g
**CULTURE PARAMETERS:**		
Volume	650 mL	1 L
Aeration rate	150 mL/min	1 L/min
Dilution rate	0.086 /h	0.09-0.1 /h
Temperature	30°C	30°C
pH (NaOH)	4	3.4
**PHENOTYPE:**		
cell density	5×10^8 cells/mL	OD 8-9
oscillation period	42-72 min	300 min

OD: optical density
