# Supplementary material for: The Yin and Yang of Yeast Transcription: Elements of a Global Feedback System between Metabolism and Chromatin
Source: PLoS One. 2012 Jun 7;7(6):e37906. doi: 10.1371/journal.pone.0037906 (PMC3369881; doi:10.1371/journal.pone.0037906)
Supplement: Table S2 — Cluster size, TSS fraction and phase angle density peaks. Cluster size, TSS fraction and phase angle density peaks. Number of genes in each cluster, fraction of cluster genes for which TSS could be found (see Methods section “Transcription Start Sites”), circular density peaks of cluster gene phase angles, and peak time (time of experiment, with the first sample as origin time 0) in the first cycle, estimated from phase angle density peaks and the cycle periods (42 min and 300 min, respectively). (PDF) [file pone.0037906.s022.pdf]

Supporting Table S2. Cluster size, TSS fraction and phase angle density peaks.

	**A**	**AB**	**B**	**B.C**	**C**	**B.D**	**D**	**l.b**	**cd.ab**	**ab.n**	**l**	**cd.n**	**n**	**r**
# of genes	414	160	135	144	388	118	640	815	132	295	475	1502	353	224
% with TSS	97.8	96.9	91.9	95.8	94.1	95.8	93.1	95.1	93.9	93.6	92	93	51.8	39.3
# with TSS	405	155	124	138	365	113	596	775	124	276	437	1397	183	88
phase angles, $^\circ$														
0.7 h	28	58	89	106	211	118	213	90	189	99	134	204	105	NA
5 h	20	43	40	83	92	294	265	39	27	39	61	42	1	3
combined	26	54	71	96	128	132	234	52	57	70	89	221	41	NA
1st peak, min														
0.7 h	24	27	31	33	45	34	45	31	42	32	36	44	33	NA
5 h	192	211	208	244	252	420	396	208	197	207	226	210	176	177

Cluster size, TSS fraction and phase angle density peaks. Number of genes in each cluster, fraction of cluster genes for which TSS could be found (see Methods section "Transcription Start Sites"), circular density peaks of cluster gene phase angles, and peak time (time of experiment, with the first sample as origin time 0) in the first cycle, estimated from phase angle density peaks and the cycle periods (42 min and 300 min, respectively).
