# Supplementary material for: The Yin and Yang of Yeast Transcription: Elements of a Global Feedback System between Metabolism and Chromatin
Source: PLoS One. 2012 Jun 7;7(6):e37906. doi: 10.1371/journal.pone.0037906 (PMC3369881; doi:10.1371/journal.pone.0037906)
Supplement: Table S3 — Significantly enriched GO terms of background clusters. Functional analysis of background Clusters. Same as Table 1 of the main article (see there for abbreviations), but for background clusters. Results for all GO terms and clusters are provided as Dataset S2. (PDF) [file pone.0037906.s023.pdf]

Supporting Table S3. Significantly enriched GO terms of background clusters.

cluster	**Cell Structure & Growth**	**Metabolism & Chromatin Regulation**	**Cell Division & Life Cycle**
l.b (815)	nuclear pore (23/51), snRNA export from nucleus (12/23), snRNP protein import into nucleus(12/23), RNA elongation from RNA PolII promoter (30/54), nuclear mRNA splicing, via spliceosome (25/72), mRNA export from nucleus (20/58), mRNA catabolic process (17/37), *ER* to Golgi vesicle-mediated transport (26/81), *ER* membrane (20/78), cytoskeleton organization (11/18), phospholipid transport (5/5)	chromatin remodeling (20/48), chromatin silencing at telomere (19/54), RSC complex (14/17), SAGA complex (9/20), histone methylation (10/17), Set1C/COMPASS complex (6/8), CCR4-NOT core complex (4/7), ISW1 complex (3/4), RENT complex (3/4)	pre-replicative complex (9/15), DNA replication initiation (11/25), cyclin-dependent protein kinase activity (6/8), G1/S (13/41) & G2/M transition of mitotic cell cycle (13/32), S phase of mitotic cell cycle (6/14)
cd.ab (132)	plasma membrane (16/215), microsome (3/20)	glycerol *BSP* (2/3), pentose transmembrane transporter activity (2/4)	
ab.n (295)	mitochondrial nucleoid (6/23)	threonine (4/5) & methionine (5/14) *MP*, homoserine (3/3), cysteine (2/2), biotin (3/6) & fatty acid (4/8) *BSP*, galactose *CP* (3/5), ATP synthesis coupled proton transport (5/20), glycolysis (5/16) & gluconeogenesis (4/15), pyruvate *MP* (3/8), vacuolar acidification (5/24)	DNA replication preinitiation complex (5/21)
l (475)	vacuolar membrane (8/31), mRNA catabolic process (9/37), AP-1 adaptor (3/6) complex, regulation of microtubule polymerization or depolymerization (5/10)	FACT complex (2/2)	DNA damage checkpoint (6/14), spindle (10/23), kinetochore (6/20), DASH (5/10) & CBF3 (3/4) complex, mitosis (4/9), septin ring assembly (4/9), cellular bud neck (18/99)
cd.n (1502)	positive regulation of transcription from RNA PolII promoter (28/71), ubiquitin-dependent protein catabolic process (51/64), autophagy (17/30), endocytosis (41/80), vacuolar transport (8/12), endosome (33/55), *ER* (84/238), actin filament organization (31/54), response to osmotic stress (19/42)	histone acetylation (19/40), condensed nuclear chromosome, centromeric region (8/12), ceramide biosynthetic process (4/4)	cellular bud tip (26/53)
n (353)	component (185/818), function (237/2049) & process (184/1313) unknown, ascospore wall (3/3) & assembly (25/43)	hexose transport (6/16)	synapsis (7/8), condensed nuclear chromosome (9/18), reciprocal meiotic recombination (12/39), meiosis (10/43), synaptonemal complex assembly (3/6), cell cycle arrest (3/3)
r (224)	component (123/818), function (111/2049) & process (119/1313) unknown, cell wall-bounded periplasmic space (4/9), endonuclease activity (6/13), cytosolic large ribosomal subunit (11/87), structural constituent of ribosome (18/216), DNA helicase activity (8/14)	thiamin biosynthetic process*BSP* (8/19), pyridoxine *MP* (5/8), asparagine *CP* (4/5), glucose transmembrane transporter activity (5/18)	telomere maintenance via recombination (7/19)

Functional analysis of background Clusters. Same as Table 1 of the main article (see there for abbreviations), but for background clusters. Results for all GO terms and clusters are provided as Dataset S2.
