# Supplementary material for: The Yin and Yang of Yeast Transcription: Elements of a Global Feedback System between Metabolism and Chromatin
Source: PLoS One. 2012 Jun 7;7(6):e37906. doi: 10.1371/journal.pone.0037906 (PMC3369881; doi:10.1371/journal.pone.0037906)
Supplement: Table S4 — Significantly enriched metabolic subsystems of clusters. Metabolic activities of clusters. Metabolic pathway or subsystem annotations for each gene were derived from a full-scale reconstruction of the metabolic network of baker’s yeast [59]. The “SUBSYSTEM” annotation was only available in the first version v1.0 of the network. Cumulative hypergeometric distribution tests were performed as described for GO analysis, and only significantly enriched subsystems are shown (). The number of genes (cluster/total) and p-values (“p”) for enrichment are given in brackets. (PDF) [file pone.0037906.s024.pdf]

Supporting Table S4. Significantly enriched metabolic subsystems of clusters.

cluster	**PATHWAY**
A (414)	Purine and Pyrimidine Biosynthesis (16/48, p=9.3e-08), Cysteine Metabolism (5/10, p=3.4e-4), Methionine Metabolism (6/16, p=5.5e-4)
AB (160)	Glycolysis/Gluconeogenesis (4/29, p=7.8e-3), Methionine Metabolism (3/16, p=8.9e-3)
B (135)	Valine, Leucine and Isoleucine (7/10, p=3.6e-10), Threonine and Lysine (6/11, p=6e-8), Histidine (6/16, p=9.5e-7), Alanine and Aspartate (4/10, p=5.3e-5), Tyrosine, Tryptophan and Phenylalanine (6/35, p=1.3e-4) & Arginine and Proline (4/26, p=2.8e-3) Metabolism, tRNA charging (8/37, p=1.5e-6), Transport, Extracellular (12/96, p=1.8-06)
B.C (144)	Oxidative Phosphorylation (18/73, p=7.7e-14), Citric Acid Cycle (10/22, p=3.3e-11), Anaplerotic reactions (3/9, p=1.1e-3), Pyruvate Metabolism (5/21, p=1.3e-4)
C (388)	Porphyrin and Chlorophyll Metabolism (5/14, p=1.6e-3), tRNA charging (13/37, p=3.6e-7)
B.D (118)	Fatty Acid Metabolism (3/3, p=8.2e-6), Arginine and Proline (6/26, p=1e-5), Anaplerotic reactions (4/9, p=1.9e-5)
D (640)	Alternate Carbon Metabolism (20/35, p=3.6e-11), Fatty Acid Degradation (7/7, 2e-7), Other (6/11, $p = 5e - 4$), Glycerolipid Metabolism (6/12, 9e-4), Xylose Metabolism (3/3, p=1.3e-3), Citric Acid Cycle (7/22, p=7.4e-3),
l.b (815)	
cd.ab (132)	Thiamine (4/6, p=3.7e-6) & Sterol (4/27, p=3e-3) Metabolism
ab.n (295)	Oxidative Phosphorylation (17/73, p=8.7e-8), Tyrosine Tryptophan and Phenylalanine (10/35, p=5.9e-06) & Arginine and Proline (6/26, p=1e-3) Metabolism, Glycolysis/Gluconeogenesis (7/29, p=4.9e-4), Fatty Acid Biosynthesis (5/22, p=4.3e-3)
l (475)	Fructose and Mannose Metabolism (3/6, p=9e-3)
cd.n (1502)	
n (353)	Transport, Extracellular (14/96, p=1.8e-3)
r (224)	Asparagine (4/5, p=1.1e-5) & Galactose (5/13, p=8.2e-05) Metabolism

Metabolic activities of clusters. Metabolic pathway or subsystem annotations for each gene were derived from a full-scale reconstruction of the metabolic network of baker's yeast [1]. The "SUBSYSTEM" annotation was only available in the first version `v1.0` of the network. Cumulative hypergeometric distribution tests were performed as described for GO analysis, and only significantly enriched subsystems are shown ($p < 0.01$). The number of genes (cluster/total) and p-values ("p") for enrichment are given in brackets.

References

1. Herrgard M, Swainston N, Dobson P, Dunn W, Arga K, et al. (2008) A consensus yeast metabolic network reconstruction obtained from a community approach to systems biology. Nat Biotechnol 26: 1155-1160.
