# Supplementary material for: The Yin and Yang of Yeast Transcription: Elements of a Global Feedback System between Metabolism and Chromatin
Source: PLoS One. 2012 Jun 7;7(6):e37906. doi: 10.1371/journal.pone.0037906 (PMC3369881; doi:10.1371/journal.pone.0037906)
Supplement: Table S5 — Enriched transcription factor binding sites and motifs. Enriched transcription factor binding sites and motifs. The presence of experimental protein binding sites (left) and DNA sequence motifs (right) in promoters and 3′UTRs were establishedd as described in the Methods section of the main article. Only significantly enriched sites/motifs ( in cumulative hypergeometric distribution tests) are shown. The numbers in brackets show the number of genes in the cluster and the total number of genes with one or more occurrences of the given motif or site in the promoter region or downstream of 3′ends (indicated by suffix “.3p”). The full set of tested bindings sites and motifs are shown in Figure S3 and provided as Datasets S5 & S6. (PDF) [file pone.0037906.s025.pdf]

Supporting Table S5. Enriched transcription factor binding sites and motifs.

	Binding Site	Motif Presence
A (414)	ABF1 (45/374), MET31 (17/80), BAS1 (14/57), MET32 (12/26), MET4 (7/27)	Sum1 (401/5414), Sfp1 (348/2942), dT7 (321/3804), A4T3 (315/2010), Sfl1 (303/3882), Stb3 (301/2030), PAC (287/1674), YBL054W (268/1057), DOT6 (258/1004), Rgt1 (166/1574), PUF4.3p (148/848), RRPE (139/559), bZIPl.3p (84/878), Bas1 (13/86)
AB (160)	FHL1 (85/182), Rap1.lieb01 (82/346), RAP1 (73/238), igRap1.lieb01 (47/291), SFP1 (35/87), YAP5 (25/126), PHO2 (17/310), GAT3 (14/50), MET31 (8/80), Sir3.lieb01 (6/43), inRap1.lieb01 (6/10), INO4 (6/65), PDR1 (4/19)	lstm.2.3p (143/4461), dT7 (122/3804), Sfp1 (117/2942), A4T3 (91/2010), Stb3 (79/2030), Gal4 (76/2062), RRPE (34/559), Rap1 (31/212), IS.Sir2 (29/566)
B (135)	GCN4 (40/220), GLN3 (18/148), GAT1 (15/72), HAP2 (15/102), ROX1 (10/175), DAL82 (10/155), RTG3 (10/130), LEU3 (7/18), igSir3.lieb01 (6/53), ARG81 (5/34), AZF1 (3/18)	Nhp6a (119/4532), lstm.2.3p (116/4461), dT7 (105/3804), GCN4 (93/2828), Gcn4 (85/2077), Gat1 (80/2511), Stb3 (68/2030), A4T3 (66/2010), GATA (53/1325), Gzf3 (51/1362), YBL054W (36/1057), DOT6 (35/1004), Lys14 (30/838), bZIPs (28/243), Stp2 (21/502)
B.C (144)	MBP1 (47/227), SWI6 (25/165), SWI4 (22/165), HAP4 (17/81), RTG3 (11/130), HAP5 (8/77), HAP2 (8/102), STB1 (6/33), ASH1 (4/32)	AREc.3p (82/2673), lstm.4 (56/1417), Fhl1 (55/1608), Mbp1 (33/259), Cbf1 (15/285)
C (388)		lstm.2.3p (327/4461), PUF3.3p (279/1793), Usv1 (39/381)
B.D (118)	GCN4 (30/220), RTG3 (18/130), UME6 (13/183), HSF1 (11/61), YAP7 (10/192), STB4 (7/11), ARG81 (4/34)	GCN4 (76/2828), TATA (69/2257), Gcn4 (64/2077), TATA.350 (61/1915), Gal4 (56/2062), Rph1 (39/1278), Ydr520c (35/1119), Ynr063w (31/935), bZIPs (22/243), Mbp1 (12/259), Ecm22 (11/215), UME6 (9/165), HSE (9/84), HSE.step (8/125), dG7 (7/67)
D (640)	MSN2 (68/267), MSN4 (60/250), SKN7 (50/214), FKH2 (45/194), UME6 (39/183), CIN5 (35/212), NDD1 (35/130), NRG1 (32/173), HAP1 (32/125), ROX1 (30/175), ACE2 (28/86), SWI5 (28/138), MCM1 (18/79), SNT2 (9/24)	STRE (385/1999), Hal9 (340/2622), TATA (301/2257), TATA.350 (266/1915), Ykl222c (241/1762), Rph1 (236/1278), Rds2 (198/1455), Yer130c (185/1078), dG5 (184/991), Oaf1 (167/1289), Sip4 (144/1014), Tbf1 (137/998), Yap1 (134/1008), Nrg1 (131/931), Ynr063w (130/935), Gsm1 (72/473), Usv1 (66/381), Cha4 (61/387), Yml081w (57/344), Stp4 (38/224), Mig3 (21/101), dG7 (19/67)
l.b (815)	ABF1 (77/374)	Sfl1 (594/3882), dT7 (573/3804), Fkh2 (467/3057), Stb3 (325/2030), Fkh1 (241/1448)
cd.ab (132)	SOK2 (12/154), SUT1 (12/127), PHD1 (12/211), NRG1 (12/173), SWI4 (11/165), CIN5 (11/212), SWI6 (11/165), THI2 (5/17), SKO1 (4/33)	dT7 (104/3804), TATA (66/2257), Spt15 (63/2154), TATA.350 (58/1915), lstm.3 (46/1469), Rsc30 (6/75)
ab.n (295)	GCN4 (44/220), CBF1 (24/215), SKN7 (20/214), HAP2 (18/102), GCR2 (16/120), HAP4 (13/81), INO4 (12/65), MET31 (11/80), HAP5 (10/77), INO2 (10/49), HAP3 (7/40), MET32 (7/26), MET4 (6/27), GCR1 (5/26), GAL4 (5/18), YML081W (3/9), GAL80 (2/2)	Gcn4 (142/2077), Pho4 (92/1233), GATA (88/1325), Met32 (49/615), bZIPs (36/243), Rtg3 (33/394), Cbf1 (28/285)
l (475)	MBP1 (30/227), CBF1 (29/215), CST6 (14/68)	Fkh2 (282/3057), Fkh1 (141/1448), Mbp1 (35/259)
cd.n (1502)	RPN4 (49/105), ADR1 (44/116)	Spt15 (598/2154)
n (353)	SUM1 (33/75), STE12 (31/327), CIN5 (24/212), UME6 (22/183), NRG1 (21/173), GAT3 (8/50), RGT1 (4/6)	TATA.350 (139/1915), Ypr013c (119/1623), Ndt80 (63/516), Ume6 (54/465), UME6 (32/165), Aft1 (22/210), Rsc3 (13/101)
r (224)	Rap1.lieb01 (70/346), Sir2.lieb01 (45/70), igRap1.lieb01 (45/291), igSir2.lieb01 (37/57), igSir4.lieb01 (27/43), Sir4.lieb01 (25/44), PHD1 (23/211), YAP6 (22/205), igSir3.lieb01 (20/53), AFT2 (20/191), CIN5 (19/212), YAP5 (19/126), FHL1 (15/182), XBP1 (14/67), Sir3.lieb01 (9/43), ig2Rap1.lieb01 (9/18), ig2Sir2.lieb01 (8/10), ig2Sir4.lieb01 (8/9), GAT3 (8/50), CST6 (8/68), ig2Sir3.lieb01 (7/8), MIG1 (6/24), THI2 (4/17)	TATA (116/2257), TATA.350 (112/1915), Smp1 (51/923), Mcm1 (29/364), Stp4 (25/224), Rsc3 (15/101), Rsc30 (10/75), Ygr067c (8/45), GCn (7/27)

Enriched transcription factor binding sites and motifs. The presence of experimental protein binding sites (left) and DNA sequence motifs (right) in promoters and 3'UTRs were establishedd as described in the Methods section of the main article. Only significantly enriched sites/motifs ($p < 0.01$ in cumulative hypergeometric distribution tests) are shown. The numbers in brackets show the number of genes in the cluster and the total number of genes with one or more occurrences of the given motif or site in the promoter region or downstream of 3'ends (indicated by suffix ".3p"). The full set of tested bindings sites and motifs are shown in Figure S7 and provided as Datasets S5 & S6.
