# Supplementary material for: The Yin and Yang of Yeast Transcription: Elements of a Global Feedback System between Metabolism and Chromatin
Source: PLoS One. 2012 Jun 7;7(6):e37906. doi: 10.1371/journal.pone.0037906 (PMC3369881; doi:10.1371/journal.pone.0037906)
Supplement: Table S6 — Data sources: URLs from which the original data was downloaded. Data Sources. The URLs from which the analyzed data was originally downloaded. If the links are not active anymore, the data can be obtained from the authors on request. (PDF) [file pone.0037906.s026.pdf]

Supporting Table S6. Data sources: URLs from which the original data was downloaded.

Data	Download URL
SGD Genome Release	ftp://ftp.yeastgenome.org/yeast/chromosomal_feature/archive/saccharomyces_cerevisiae.gff.20080202.gz
0.7 h period transcr. [1]	from authors
5 h period transcr. [2]	http://yeast.swmed.edu/PUBLIC_DATA/
Metabolic network [3]	http://www.comp-sys-bio.org/yeastnet/YeastMetabolicNetwork-nocomp-1.0.xml
Transcr. Compendium [4]	http://thebrain.bwh.harvard.edu/CRACR/1327cond.txt
Nucl. occ. WT [5]	http://chemogenomics.stanford.edu/supplements/03nuc/files/analyzed_data_complete_bw20.txt
Nucl. classes [5]	http://chemogenomics.stanford.edu/supplements/03nuc/files/clusters/polyA_segments_verified_K_G4.kgg
Nucl. occ. WT [6]	http://labs.fhcrc.org/tsukiyama/supplemental_data/global_nucleosome_mapping/WT_nucs.sgr.zip
Nucl. occ. isw2$^{-/-}$ [6]	http://labs.fhcrc.org/tsukiyama/supplemental_data/global_nucleosome_mapping/isw2_nucs.sgr.zip
Nucl. Remod. Score [6]	http://labs.fhcrc.org/tsukiyama/supplemental_data/global_nucleosome_mapping/Chr_remodelling.sgr.zip
Isw2 ChIP [6]	http://labs.fhcrc.org/tsukiyama/supplemental_data/global_nucleosome_mapping/Isw2_ChIP.sgr.zip
Remod./Isw2 classes [6]	http://www.nature.com/nature/journal/v450/n7172/extref/nature06391-s2.xls
Transcr. data [7]	http://www.ebi.ac.uk/huber-srv/actinomycinD/actD_normalized.txt.gz
TSS data [7]	http://www.ebi.ac.uk/huber-srv/actinomycinD/Supplementary%20Table2.xls
TSS data [8]	ftp://genome-ftp.stanford.edu/pub/yeast/data_download/systematic_results/transcription/Miura_2006_pmid_17101987/Miura2006_cDNAclones.gff
TSS data [9]	ftp://genome-ftp.stanford.edu/pub/yeast/data_download/systematic_results/transcription/Zhang_2005_pmid_15905473/Zhang2005_TSS.gff
TF Binding data [10]	http://fraenkel.mit.edu/improved_map/p005_c1.gff
TF Motif data [11]	http://the_brain.bwh.harvard.edu/pbms/webworksY/downloads/yeast_pwm_all.zip
ESR & GRR classes [12]	http://www.molbiolcell.org/content/vol0/issue2007/images/data/E07-08-0779/DC1/TableS1.xls
TFIID/SAGA classes [13]	http://www.molecule.org/cgi/content/full/13/4/573/DC1/Table%20S2.xls
Transcr. freq. [14]	http://web.wi.mit.edu/young/pub/data/orf_transcriptome.txt
Protein levels [15]	http://www.nature.com/nature/journal/v425/n6959/extref/nature02046-s2.xls
Expr. noise [16]	http://www.nature.com/nature/journal/v441/n7095/extref/nature04785-s04.xls
RNA half-life [17]	http://www-genome.stanford.edu/turnover/Yuleidatafiles/halflifeTable.txt
rsc3-1, *rsc30*Δ expr. [18]	http://download.cell.com/molecular-cell/mmcs/journals/1097-2765/PIIS1097276501002192.mmc2.xls
Rsc9 locations [19]	http://www.sciencedirect.com/science/article/pii/S1097276502004756#MMCvA_2
CRE score [20]	from authors
RSC chrIII remod. class [21]	from authors
RSC ChIP [22]	http://jura.wi.mit.edu/young_public/RSC/data/RSC_genelist_new.txt
Nucl. occ. WT [23]	http://genome.icmb.utexas.edu/nucleosome/normal.wig.gz
Nucl. occ. EtOH [24]	http://genie.weizmann.ac.il/pubs/nucleosomes08/data/YPEtOH_dMean.chv.gz
Δ Transcr. & Nucl. occ. [25]	http://hugheslab.ccbr.utoronto.ca/supplementary-data/yeastDBD/normalized-txt-files.zip
Rsc8 ChIP [25]	http://hugheslab.ccbr.utoronto.ca/supplementary-data/yeastDBD/Rsc8_ChIP-chip_data.zip
Diverse DIP ChIP [25]	http://hugheslab.ccbr.utoronto.ca/supplementary-data/yeastDBD/DIP_chip_GFF_files.zip

Data Sources. The URLs from which the analyzed data was originally downloaded. If the links are not active anymore, the data can be obtained from the authors on request.

References

1. Li CM, Klevecz RR (2006) A rapid genome-scale response of the transcriptional oscillator to perturbation reveals a period-doubling path to phenotypic change. Proc Natl Acad Sci U S A 103: 16254-9.

2. Tu BP, Kudlicki A, Rowicka M, McKnight SL (2005) Logic of the yeast metabolic cycle: temporal compartmentalization of cellular processes. Science 310: 1152-8.

3. Herrgard M, Swainston N, Dobson P, Dunn W, Arga K, et al. (2008) A consensus yeast metabolic network reconstruction obtained from a community approach to systems biology. Nat Biotechnol 26: 1155-1160.

4. McCord R, Berger M, Philippakis A, Bulyk M (2007) Inferring condition-specific transcription factor function from DNA binding and gene expression data. Mol Syst Biol 3: 100.

5. Lee W, Tillo D, Bray N, Morse R, Davis R, et al. (2007) A high-resolution atlas of nucleosome occupancy in yeast. Nat Genet 39: 1235-1244.

6. Whitehouse I, Rando O, Delrow J, Tsukiyama T (2007) Chromatin remodelling at promoters suppresses antisense transcription. Nature 450: 1031-1035.

7. Perocchi F, Xu Z, Clauder-Munster S, Steinmetz L (2007) Antisense artifacts in transcriptome microarray experiments are resolved by actinomycin D. Nucleic Acids Res 35: e128.

8. Miura F, Kawaguchi N, Sese J, Toyoda A, Hattori M, et al. (2006) A large-scale full-length cDNA analysis to explore the budding yeast transcriptome. Proc Natl Acad Sci U S A 103: 17846-51.

9. Zhang Z, Dietrich FS (2005) Mapping of transcription start sites in Saccharomyces cerevisiae using 5' SAGE. Nucleic Acids Res 33: 2838-51.

10. MacIsaac K, Wang T, Gordon D, Gifford D, Stormo G, et al. (2006) An improved map of conserved regulatory sites for Saccharomyces cerevisiae. BMC Bioinformatics 7: 113.

11. Zhu C, Byers K, McCord R, Shi Z, Berger M, et al. (2009) High-resolution DNA-binding specificity analysis of yeast transcription factors. Genome Res 19: 556-566.

12. Brauer M, Huttenhower C, Airoldi E, Rosenstein R, Matese J, et al. (2008) Coordination of growth rate, cell cycle, stress response, and metabolic activity in yeast. Mol Biol Cell 19: 352-367.

13. Huisinga KL, Pugh BF (2004) A genome-wide housekeeping role for TFIID and a highly regulated stress-related role for SAGA in Saccharomyces cerevisiae. Mol Cell 13: 573-85.

14. Holstege F, Jennings E, Wyrick J, Lee T, Hengartner C, et al. (1998) Dissecting the regulatory circuitry of a eukaryotic genome. Cell 95: 717-728.

15. Ghaemmaghami S, Huh W, Bower K, Howson R, Belle A, et al. (2003) Global analysis of protein expression in yeast. Nature 425: 737-741.

16. Newman J, Ghaemmaghami S, Ihmels J, Breslow D, Noble M, et al. (2006) Single-cell proteomic analysis of S. cerevisiae reveals the architecture of biological noise. Nature 441: 840-846.

17. Wang Y, Liu C, Storey J, Tibshirani R, Herschlag D, et al. (2002) Precision and functional specificity in mRNA decay. Proc Natl Acad Sci U S A 99: 5860-5865.

18. Angus-Hill M, Schlichter A, Roberts D, Erdjument-Bromage H, Tempst P, et al. (2001) A rsc3/rsc30 zinc cluster dimer reveals novel roles for the chromatin remodeler RSC in gene expression and cell cycle control. Mol Cell 7: 741-751.

19. Damelin M, Simon I, Moy T, Wilson B, Komili S, et al. (2002) The genome-wide localization of rsc9, a component of the RSC chromatin-remodeling complex, changes in response to stress. Mol Cell 9: 563-573.

20. Choi J, Kim Y (2008) Epigenetic regulation and the variability of gene expression. Nat Genet 40: 141-147.

21. Hartley P, Madhani H (2009) Mechanisms that specify promoter nucleosome location and identity. Cell 137: 445-458.

22. Ng H, Robert F, Young R, Struhl K (2002) Genome-wide location and regulated recruitment of the RSC nucleosome-remodeling complex. Genes Dev 16: 806-819.

23. Shivaswamy S, Bhinge A, Zhao Y, Jones S, Hirst M, et al. (2008) Dynamic remodeling of individual nucleosomes across a eukaryotic genome in response to transcriptional perturbation. PLoS Biol 6: e65.

24. Kaplan N, Moore I, Fondufe-Mittendorf Y, Gossett A, Tillo D, et al. (2009) The DNA-encoded nucleosome organization of a eukaryotic genome. Nature 458: 362-366.

25. Badis G, Chan E, van Bakel H, Pena-Castillo L, Tillo D, et al. (2008) A library of yeast transcription factor motifs reveals a widespread function for rsc3 in targeting nucleosome exclusion at promoters. Mol Cell 32: 878-887.
