# Supplementary material for: The Yin and Yang of Yeast Transcription: Elements of a Global Feedback System between Metabolism and Chromatin
Source: PLoS One. 2012 Jun 7;7(6):e37906. doi: 10.1371/journal.pone.0037906 (PMC3369881; doi:10.1371/journal.pone.0037906)
Supplement: Table S7 — Saccharomyces cerevisiae strains used in analyzed datasets. Strain information for all datasets used in this study, derived from original publications. (PDF) [file pone.0037906.s027.pdf]

Supporting Table S7. ***Saccharomyces cerevisiae*** **strains used in analyzed datasets.**

**Data**	**Strains (genotypes)**
SGD Genome Release	S288C
0.7 h period transcriptome [1]	IFO 0233
5 h period transcriptome [2]	CEN.PK122
Metabolic network [3]	mapped to S288c
Transcriptome compendium [4]	data compendium from many strains
Nucleosome occupancy [5]	BY4741, parent strain of the Mat-a yeast knockout collection
Nucleosome occupancy, isw2$^{-/-}$, Isw2 ChIP [6]	S288C, S288C (Δ*isw2*)
Transcription and TSS data [7]	S1003 (*MAT***a**/α *gal2/gal2 lys5/lys5*), a S288C back-ground strain)
full-length cDNA TSS data [8]	S288C NBRC1136 (*MAT*α *SUC2 mal mel gal2 CUP1 [cir+]*), S288C ATCC204722 (*MAT***a**/α *HO can1 gal2 cup1*)
5'SAGE TSS data [9]	W303-1A (*MAT***a** *ura3-1 leu2-3112 trp1-1 can1-100 ade2-1 his3-11,15 [psi+]*)
TF binding sites [10] generated from ChIP data in [11]	W303 strain Z1256
TF Motif data [12]	*in vitro*
ESR classes [13]	DBY7286, DBY8768, DBY9434, DBY9435, DBY9439, DBY9440, DBY9441
GRR classes [14]	DBY10085, DBY9492 *ura3-52*, DBY9497 *leu2-3 leu2-11*; all (*MAT***a** MAL2-8C)
TFIID/SAGA classes [15]	YJS6, YKH100, YJS7, YJS8, YKH101, YJS9, YKH105, YKH104, YKH106, YKH108, YKH107, YKH109
Transcriptional frequencies [16]	many, see `http://web.wi.mit.edu/young/expression/genotype.html`
Protein levels [17]	ATCC 201388 (*MAT***a** *his3*Δ*1 leu2*Δ*0 met15*Δ*0 ura3*Δ*0*)
Expression noise [18] *via* [19]	ATCC 201388 (*MAT***a** *his3*Δ*1 leu2*Δ*0 met15*Δ*0 ura3*Δ*0*)
RNA half-life [20]	Y262 (*MAT***a** *ura3-52 his4-939am rpb1-1*)
rsc3-1, *rsc30*Δ expr. [21]	S288C derivates YBC76 and YBC610
Rsc9 locations [22]	PSY2439 (Z1256 with myc-tagged RSC9
CRE score [23]	meta-study
RSC chrIII remodeling [24]	YM3202, YM3531, YM3532, YM3533, YM3534, YM3535, YM3536, YM3537, YM3538, YM3539, YM3540, YM3541, YM3542
	see Table S1 in `http://www.cell.com/supplemental/S0092-8674%2809%2900257-8`
RSC ChIP [25]	FT4 (*MAT***a** *ura3-52 trp1-*Δ*63 his3-*Δ*200 leu2::PET56*)
Nucleosome occupancy [26]	S288C
Nucleosome occupancy [27]	BY4741
Transcription, Nucleosome occupany, Rsc8-ChIP [28]	*abf1-101*, *cep3*, *mcm1-1*, *rap1-1*, *reb1-212*, *rsc3-1*, *tbf1*, *rsc3-1* Rsc8-TAP, Rsc8-TAP
	see Table S3 in `http://hugheslab.ccbr.utoronto.ca/supplementary-data/yeastDBD/PIIS1097276508008423.mmc1.pdf`

Strain information for all datasets used in this study, derived from original publications.

References

1. Li CM, Klevecz RR (2006) A rapid genome-scale response of the transcriptional oscillator to perturbation reveals a period-doubling path to phenotypic change. Proc Natl Acad Sci U S A 103: 16254-9.

2. Tu BP, Kudlicki A, Rowicka M, McKnight SL (2005) Logic of the yeast metabolic cycle: temporal compartmentalization of cellular processes. Science 310: 1152-8.

3. Herrgard M, Swainston N, Dobson P, Dunn W, Arga K, et al. (2008) A consensus yeast metabolic network reconstruction obtained from a community approach to systems biology. Nat Biotechnol 26: 1155-1160.

4. McCord R, Berger M, Philippakis A, Bulyk M (2007) Inferring condition-specific transcription factor function from DNA binding and gene expression data. Mol Syst Biol 3: 100.

5. Lee W, Tillo D, Bray N, Morse R, Davis R, et al. (2007) A high-resolution atlas of nucleosome occupancy in yeast. Nat Genet 39: 1235-1244.

6. Whitehouse I, Rando O, Delrow J, Tsukiyama T (2007) Chromatin remodelling at promoters suppresses antisense transcription. Nature 450: 1031-1035.

7. Perocchi F, Xu Z, Clauder-Munster S, Steinmetz L (2007) Antisense artifacts in transcriptome microarray experiments are resolved by actinomycin D. Nucleic Acids Res 35: e128.

8. Miura F, Kawaguchi N, Sese J, Toyoda A, Hattori M, et al. (2006) A large-scale full-length cDNA analysis to explore the budding yeast transcriptome. Proc Natl Acad Sci U S A 103: 17846-51.

9. Zhang Z, Dietrich FS (2005) Mapping of transcription start sites in Saccharomyces cerevisiae using 5' SAGE. Nucleic Acids Res 33: 2838-51.

10. MacIsaac K, Wang T, Gordon D, Gifford D, Stormo G, et al. (2006) An improved map of conserved regulatory sites for Saccharomyces cerevisiae. BMC Bioinformatics 7: 113.

11. Harbison C, Gordon D, Lee T, Rinaldi N, Macisaac K, et al. (2004) Transcriptional regulatory code of a eukaryotic genome. Nature 431: 99-104.

12. Zhu C, Byers K, McCord R, Shi Z, Berger M, et al. (2009) High-resolution DNA-binding specificity analysis of yeast transcription factors. Genome Res 19: 556-566.

13. Gasch A, Spellman P, Kao C, Carmel-Harel O, Eisen M, et al. (2000) Genomic expression programs in the response of yeast cells to environmental changes. Mol Biol Cell 11: 4241-4257.

14. Brauer M, Huttenhower C, Airoldi E, Rosenstein R, Matese J, et al. (2008) Coordination of growth rate, cell cycle, stress response, and metabolic activity in yeast. Mol Biol Cell 19: 352-367.

15. Huisinga KL, Pugh BF (2004) A genome-wide housekeeping role for TFIID and a highly regulated stress-related role for SAGA in Saccharomyces cerevisiae. Mol Cell 13: 573-85.

16. Holstege F, Jennings E, Wyrick J, Lee T, Hengartner C, et al. (1998) Dissecting the regulatory circuitry of a eukaryotic genome. Cell 95: 717-728.

17. Ghaemmaghami S, Huh W, Bower K, Howson R, Belle A, et al. (2003) Global analysis of protein expression in yeast. Nature 425: 737-741.

18. Newman J, Ghaemmaghami S, Ihmels J, Breslow D, Noble M, et al. (2006) Single-cell proteomic analysis of S. cerevisiae reveals the architecture of biological noise. Nature 441: 840-846.

19. Huh W, Falvo J, Gerke L, Carroll A, Howson R, et al. (2003) Global analysis of protein localization in budding yeast. Nature 425: 686-691.

20. Wang Y, Liu C, Storey J, Tibshirani R, Herschlag D, et al. (2002) Precision and functional specificity in mRNA decay. Proc Natl Acad Sci U S A 99: 5860-5865.

21. Angus-Hill M, Schlichter A, Roberts D, Erdjument-Bromage H, Tempst P, et al. (2001) A rsc3/rsc30 zinc cluster dimer reveals novel roles for the chromatin remodeler RSC in gene expression and cell cycle control. Mol Cell 7: 741-751.

22. Damelin M, Simon I, Moy T, Wilson B, Komili S, et al. (2002) The genome-wide localization of rsc9, a component of the RSC chromatin-remodeling complex, changes in response to stress. Mol Cell 9: 563-573.

23. Choi J, Kim Y (2008) Epigenetic regulation and the variability of gene expression. Nat Genet 40: 141-147.

24. Hartley P, Madhani H (2009) Mechanisms that specify promoter nucleosome location and identity. Cell 137: 445-458.

25. Ng H, Robert F, Young R, Struhl K (2002) Genome-wide location and regulated recruitment of the RSC nucleosome-remodeling complex. Genes Dev 16: 806-819.

26. Shivaswamy S, Bhinge A, Zhao Y, Jones S, Hirst M, et al. (2008) Dynamic remodeling of individual nucleosomes across a eukaryotic genome in response to transcriptional perturbation. PLoS Biol 6: e65.

27. Kaplan N, Moore I, Fondufe-Mittendorf Y, Gossett A, Tillo D, et al. (2009) The DNA-encoded nucleosome organization of a eukaryotic genome. Nature 458: 362-366.

28. Badis G, Chan E, van Bakel H, Pena-Castillo L, Tillo D, et al. (2008) A library of yeast transcription factor motifs reveals a widespread function for rsc3 in targeting nucleosome exclusion at promoters. Mol Cell 32: 878-887.
