# Supplementary material for: The Yin and Yang of Yeast Transcription: Elements of a Global Feedback System between Metabolism and Chromatin
Source: PLoS One. 2012 Jun 7;7(6):e37906. doi: 10.1371/journal.pone.0037906 (PMC3369881; doi:10.1371/journal.pone.0037906)
Supplement: Table S8 — Coordinate-based Data for SDP Plots. This table maps y-axis labels of SDP plots to a data ID used in the underlying data collection. This collection is provided as a big archive file (295 MB) at http://www.tbi.univie.ac.at/raim/data/2011/yeast/clusters/geneData.tar.gz. Each file in the archive corresponds to one SDP. The rows are all genes for which a TSS could be defined (see Methods of the main paper), and the columns give values for each position from −1500 upstream to +1500 downstream of the TSS (+1). TSS were aligned to the genome in the SGD release from Feb. 2008. The main results and underlying data of this paper are made available in CSV format (comma-separated values) at http://www.tbi.univie.ac.at/raim/data/2011/yeast/clusters/. In the following, the content of each file (column headers are in quotes) is described in detail: (PDF) [file pone.0037906.s028.pdf]

Supporting Table S8. Coordinate-based Data for SDP Plots.

ID	SDP plot label	Reference
rNucl.rsc3	Δ Nucl. Occ. - *rsc3-1*	[1]
rNucl.abf1	Δ Nucl. Occ. - *abf1-101*	[1]
rNucl.mcm1	Δ Nucl. Occ. - *mcm1*	[1]
rNucl.rap1	Δ Nucl. Occ. - *rap1-1*	[1]
rNucl.reb1	Δ Nucl. Occ. - *reb1-212*	[1]
rNucl.tbf1	Δ Nucl. Occ. - *tbf1*	[1]
rNucl.cep3	Δ Nucl. Occ. - *cep3-1*	[1]
rsc3.TS.p	Δ RNA_{sense} - *rsc3-1*	[1]
abf1.TS.p	Δ RNA_{sense} - *abf1-101*	[1]
mcm1.TS.p	Δ RNA_{sense} - *mcm1*	[1]
rap1.TS.p	Δ RNA_{sense} - *rap1-1*	[1]
reb1.TS.p	Δ RNA_{sense} - *reb1-212*	[1]
tbf1.TS.p	Δ RNA_{sense} - *tbf1*	[1]
cep3.TS.p	Δ RNA_{sense} - *cep3-1*	[1]
rsc3.TS.m	Δ $RNA_{antisense}$ - *rsc3-1*	[1]
abf1.TS.m	Δ $RNA_{antisense}$ - *abf1-101*	[1]
mcm1.TS.m	Δ $RNA_{antisense}$ - *mcm1*	[1]
rap1.TS.m	Δ $RNA_{antisense}$ - *rap1-1*	[1]
reb1.TS.m	Δ $RNA_{antisense}$ - *reb1-212*	[1]
tbf1.TS.m	Δ $RNA_{antisense}$ - *tbf1*	[1]
cep3.TS.m	Δ $RNA_{antisense}$ - *cep3-1*	[1]
Rsc8.Rsc3.badis08	Rsc8 ChIP in *rsc3-1*	[1]
Rsc8.badis08	Rsc8 ChIP in WT	[1]
Rap1.badis08	Rap1 DIP	[1]
Nucl.Lee	Nucl. Occ., Lee et al.	[2]
Nucl.WT	Nucl. Occ., Whitehouse et al.	[3]
Nucl.norm	Nucl. Occ., Shivaswamy et al.	[4]
Nucl.heat	Nucl. Occ., heat-shocked, Shivaswamy et al.	[4]
Nucl.EtOH	Nucl. Occ., on ethanol, Kaplan et al.	[5]
Nucl.Gal	Nucl. Occ., on galactose, Kaplan et al.	[5]
Nucl.inVitro	Nucl. Occ., in vitro, Kaplan et al.	[5]
Isw2	Isw2(K215R) ChIP (enrichment over WT)	[3]
TS.p	RNA_{sense} , Perocchi et al.	[6]
TS.m	$RNA_{antisense}$, Perocchi et al.	[6]
Nt	Nucleotide sequence	SGD, Feb. 2008

This table maps y-axis labels of SDP plots to a data ID used in the underlying data collection. This collection is provided as a big archive file (295 MB) at
`http://www.tbi.univie.ac.at/~raim/data/2011/yeast/clusters/geneData.tar.gz`.
Each file in the archive corresponds to one SDP. The rows are all genes for which a TSS could be defined (see Methods of the main paper), and the columns give values for each position from -1500 upstream to +1500 downstream of the TSS (+1). TSS were aligned to the genome in the SGD release from Feb. 2008.

References

1. Badis G, Chan E, van Bakel H, Pena-Castillo L, Tillo D, et al. (2008) A library of yeast transcription factor motifs reveals a widespread function for rsc3 in targeting nucleosome exclusion at promoters. Mol Cell 32: 878-887.

2. Lee W, Tillo D, Bray N, Morse R, Davis R, et al. (2007) A high-resolution atlas of nucleosome occupancy in yeast. Nat Genet 39: 1235-1244.

3. Whitehouse I, Rando O, Delrow J, Tsukiyama T (2007) Chromatin remodelling at promoters suppresses antisense transcription. Nature 450: 1031-1035.

4. Shivaswamy S, Bhinge A, Zhao Y, Jones S, Hirst M, et al. (2008) Dynamic remodeling of individual nucleosomes across a eukaryotic genome in response to transcriptional perturbation. PLoS Biol 6: e65.

5. Kaplan N, Moore I, Fondufe-Mittendorf Y, Gossett A, Tillo D, et al. (2009) The DNA-encoded nucleosome organization of a eukaryotic genome. Nature 458: 362-366.

6. Perocchi F, Xu Z, Clauder-Munster S, Steinmetz L (2007) Antisense artifacts in transcriptome microarray experiments are resolved by actinomycin D. Nucleic Acids Res 35: e128.
