# Supplementary material for: The Yin and Yang of Yeast Transcription: Elements of a Global Feedback System between Metabolism and Chromatin
Source: PLoS One. 2012 Jun 7;7(6):e37906. doi: 10.1371/journal.pone.0037906 (PMC3369881; doi:10.1371/journal.pone.0037906)
Supplement: Text S1 — outlines problems with global microarray normalization and the choice of a “least-oscillating set” of genes as an alternative normalization reference (S1.1), the choice of DFT components for clustering (S1.2) and a general reasoning behind our clustering approach and the chosen algorithm (S1.3). (PDF) [file pone.0037906.s037.pdf]

The Yin and Yang of Yeast Transcription: Elements of a Global Feedback System Between Metabolism and Chromatin.

Supporting Text S1

Rainer Machné[1,*], Douglas B. Murray[2]

1 Institute for Theoretical Chemistry, University of Vienna, Austria

2 Institute for Advanced Biosciences, Keio University, Japan

∗ **E-mail: raim@tbi.univie.ac.at**

Contents

S1 Supporting Text **2**

S1.1 Normalization Problems & "Least-Oscillating" Reference Sets 2

S1.2 Selection of DFT Components for Clustering 3

S1.3 Choice of the Clustering Algorithm `flowClust` 4

List of Figures

ST1 Global Effects of Normalization. 6

ST2 Fourier Analysis of Gene Expression Data 7

ST3 DFT-based Clustering of Li and Klevecz 2006 Data 8

ST4 DFT-based Clustering of Tu et al. 2005 Data 9

S1 Supporting Text

S1.1 Normalization Problems & "Least-Oscillating" Reference Sets

The processing of microarray datasets usually involves a normalization step which serves to remove systematic non-biological array-to-array variation and commonly employed methods rely on a reference set of genes that do not vary between samples or assume that the majority of transcripts remain unchanged over the experiment. The original publications reported that a majority of transcripts oscillated in the datasets analyzed here [1–3]. Thus common microarray normalization methods may introduce an artificial bias for our datasets. A less-well discussed but related problem is an experimental normalization step present in typical microarray protocols. This step assumes a constant total RNA content of cells between experimental conditions or over a time-series. Thus, the protocols require that the same amount of RNA is applied from each sample with the goal to remove variations in RNA extraction efficiency between samples. However, if the actual total RNA content per cell, or only the fraction of RNA transcripts present in the extract but not probed on the array, undergoes periodic changes, then this step will superpose this periodicity on all measured transcripts. For example, ribosomal RNA constitutes the majority of total RNA and has been shown to undergo specific degradation processes in starvation conditions [4] which can in principle be imagined to (transiently) occur over the respiratory oscillation (see ATP:ADP ratio in Fig. 3b of the main article). Indeed, several tested algorithmic normalization strategies lead to an oscillation of the total chip signal and to global shifts of the phase angles ϕ_{k_c} (calculated from the DFT components k_c, corresponding to the number of phenotypic cycles in the experiment, see Methods section of the main article), between different normalization methods and raw data (Fig. ST1). This clearly indicates that at least one of the two normalization steps did indeed introduce a global bias and at least low-level oscillations in the dataset have to be taken with a grain of salt.

Thus, we refrain from any global microarray normalization for data analysis (DFT-based clustering). We do, however, use a DFT-based normalization strategy for clarity of data visualization (Fig. 1a and 1b of the main article). The approach is similar to the "least-variant set of genes" (LVS) normalization [5] (available as the R package `FLUSH.LVS.bundle`) but using a custom-made definition of "least-oscillating set" of probe sets as a normalization reference. The LOS set was defined as those with an oscillation p-value $p_{k_c} > 0.6$ (see below, section S1.2), using all probe sets on the Affymetrix arrays `YG_S98` (9,307 probe sets) and `Yeast_2` (10,928), which also contain sequences from intergenic regions of *S. cerevisiae*,

and from protein-coding genes of *S. pombe* and other organisms (where the fluorescence stems from cross-hybridization). This resulted in reference sets of 198 probe sets for the `Yeast_2` array (0.7 h cycle dataset, [2]) and 527 probe sets for the `YG_S98` array (5 h cycle dataset, [3]). For each array a `LOESS` fit, local fitting of a (here) 2nd order polynomial with span parameter $\alpha = 0.75$ (R function `loess`), was calculated between the intensity values of the reference probe set and their median over the complete time series. This fit was then used to interpolate values for all other probe sets (equivalent to the LVS normalization with the `FLUSH.LVS.bundle` R package, function `normalize.lvs` with "ref.fun=median" and "use.loess=TRUE" settings). The raw values of the time-series and the p-values p_{k_c} are given in the Dataset 1 to ensure reproducibility.

This strategy avoids the global bias of phase angles introduced by other normalization methods (Fig. ST1F and ST1H). It may however introduce other biases such as damping of potentially real (non-noise) signals at time-scales different from the respiratory oscillation. Thus, in general, we would not recommend to use even this normalization for other then visualization purposes without inspection of the raw data and further investigation of the outlined problems.

S1.2 Selection of DFT Components for Clustering

The amplitude at the DFT component k_c, corresponding to the number of phenotypic cycles in the experiment, only carries the harmonic (sine-like) contribution to the real signal. Since RNA transcription and degradation involve a variety of mechanisms and kinetics, more complex wave-forms can be expected in the real dataset. These would result in further contributions at upper harmonic cycle numbers (integer multiples of k_c).

To estimate the significance of periodicity at a given cycle number k of a transcript time series a permutation test can be applied [6–8]. Random scaled amplitudes $\tilde{a}_k$ were calculated for a series of $M = 50,000$ random permutations of the time series x. The frequency of permutations where the random amplitude is larger then the observed amplitude, $p_k = \frac{N_{\tilde{a}_k \geq a_k}}{M}$, is then interpreted as a probability (p-value) to observe the given amplitude a_k by random chance. Fluorescence profiles associated with 4,160 yeast transcripts in the 0.7 h dataset and 3,201 in 5 h dataset have a p-value $p_{k_c} < 0.01$ at the respective phenotypic cycle number k_c, but we indeed find significant contributions at upper harmonic periods in both datasets (Fig. ST2A).

Additionally, the time series measurement in dataset [2] included an experimental interference (ad-

dition of phenelzine after the first cycle) resulting in an increase of the phenotypic (dissolved O_2) and transcriptome oscillation periods [2]. This period increase most likely explains the significant contributions at $k = \{1, 3\}$ (Fig. ST2A, left plot). System drift, *e. g.*, the change in amplitude in the DOT curve seen in figure 1 of the original publication of the 5 h period dataset [3], can be expected to contribute to DFT components at $k = 1$. Variations in the temporal spacing of sampling times, perhaps more likely for the 25 min sampling in the 5 h period dataset, may lead to contributions at DFT components adjacent to k_c. Finally, random array-to-array variation (real noise) is expected to contribute mostly to higher cycle numbers. Based on these observations the cycle numbers $k_s = \{1, 3, 4, 5, 8, 12\}$ (Fig. ST3A) and $k_s = \{1, 2, 3, 4, 6, 9\}$ (Fig. ST4A) were selected for the 0.7 h [2] and the 5 h [3] period datasets, respectively, for clustering analysis.

S1.3 Choice of the Clustering Algorithm `flowClust`

Several in part inter-related problems in pre-processing and clustering of time series data of microarray time series data led to our data processing strategy and choice of the clustering algorithm. Firstly, and independent of clustering, global normalization of a set of microarrays becomes problematic when some critical assumptions are not met, and we show above (Section S1.1) that the experimental systems in question and the resulting datasets clearly violate standard assumptions. Our strategy allows to avoid prior normalization, since the non-biological array-to-array variability will be mainly present in high-frequency components of the DFT, which were not subjected to the clustering algorithm (Section S1.2). Secondly, a general problem with clustering is the choice of the number of clusters [9]. Model-based clustering, which assumes a mix of several normal distributions ("Gaussian mixture model") in each data dimension, has previously been applied to appropriately transformed microarray data and is available as the `R` package `MClust` [10]. One major advantage of model-based clustering is, that it implicitly provides information, the "Bayesian Information Criterion" (BIC), which helps to choose the best number of clusters that is supported by the data (Fig. ST3B & ST4B). Our specific choice of the `flowClust` algorithm, however, depended on further facets of the given clustering problem.

Thirdly, when subjecting a pure time series to a clustering algorithm the most important information is lost, namely the pattern of change, since data dimensions are usually treated independently of each other by common clustering algorithms. An increasing number of approaches deal with this issue [11–20], but there is no accepted standard solution. One group applied a model-based clustering algorithm to

Fourier coefficients of transcript time series [21, 22], very similar to our approach. Finally, however, microarray fluorescence is not a quantitative measure of transcript levels, mostly due to the strong sequence-dependent variability of hybridization energies [23]. The dependence of fluorescence on RNA transcript concentration may be linear, but the slope of the curve can again strongly differ between transcripts. Thus, amplitudes only have semiquantitative character and we further want to de-emphasize it in favor of the overall pattern of change, when clustering microarray time series. This is in part handled by our amplitude scaling (see Methods section of the main manuscript), which, to some extent, normalizes the given amplitude by the overall variability of the time-series. However, when inspecting the polar plots of the scaled DFT components (whose x- and y-dimensions were subjected to clustering) or the raw time-series data, we can see that a few strong oscillatory transcripts have a higher amplitude than many other genes at the same phase (Fig. ST3A & ST4A). Thus, the distributions of the individual suspected clusters are not Gaussian but tailed. This observation led to the final choice of `flowClust` as a clustering algorithm. It was initially designed for clustering of flow cytometry data [24, 25] but the algorithm is obviously agnostic to the source of data; it is built to handle tailed distributions ("t-mixture model"). In short, the algorithm fits the parameter λ of a Box-Cox transformation, which allows to transform a given tailed into a more "Gaussian-like" distribution. In flow cytometry of surface-labelled cell populations, these data tails represent cells that show the strongest signal within a given class of cells. In our case, the tails of distributions in the polar plot reflect high amplitudes of the fluorescence signal. Thus, we reasoned that `flowClust` is the best choice for our clustering strategy. We tested several algorithms by the criteria described in the Methods sections (2-objective plots [9], automated ranking of cluster validation criteria [26] and, for model-based algorithms, the BIC). However, the usability of such validation measures was limited and manual inspection of clustered time-series data contributed most to the evaluation of clustering quality and stability. Indeed, `flowClust` provided the most consistent and stable results. These observations were subject of and confirmed by a recent more systematic computational analysis of clustering strategies of circadian transcriptome data from the cyanobacterium *Synechocystis sp.* PCC6803 (Lehmann R. *et al.*, manuscript in preparation).

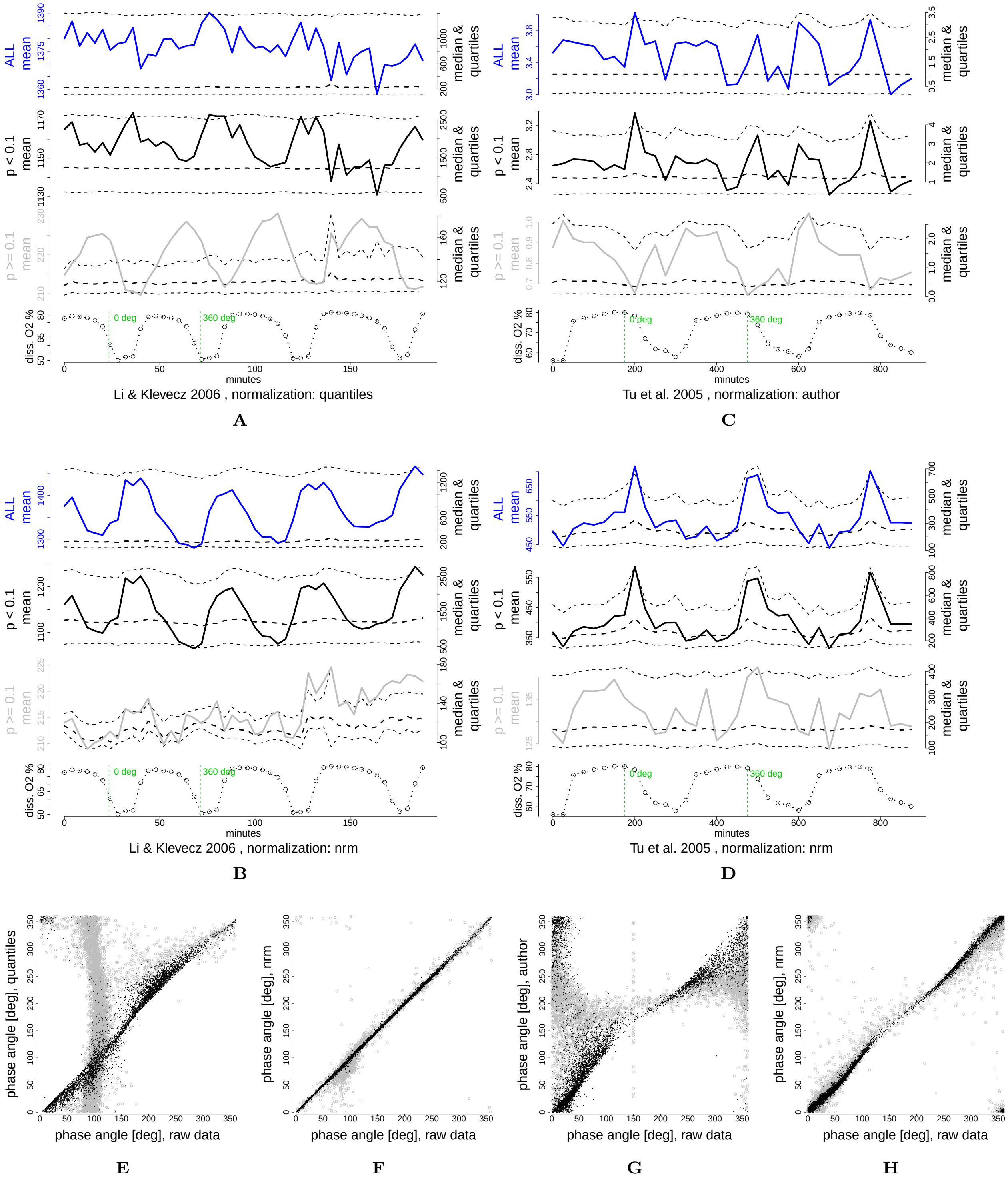

Supporting Text Figure ST1. Global effects of normalization. ST1A–ST1D: average chip signal per probe set (after summarization), for all probe sets (blue), and split between significantly periodic (black, p-value $p_{k_c} < 0.1$) and non-periodic (gray, $p_{k_c} >= 0.1$) probe sets. Median and quartiles are shown as dashed and dotted gray lines (right y-axis), respectively. ST1E–ST1H: phase angles ϕ_{k_c} from raw *vs.* normalized data, (black and gray colors as above). ST1A and ST1E: "quantiles" normalization of the dataset from [2]. ST1C and ST1G: *median polishing* as used in the original publication of this dataset [3]. ST1B, ST1D, ST1F and ST1H: normalization of datasets using "least-oscillating" probe sets as reference.

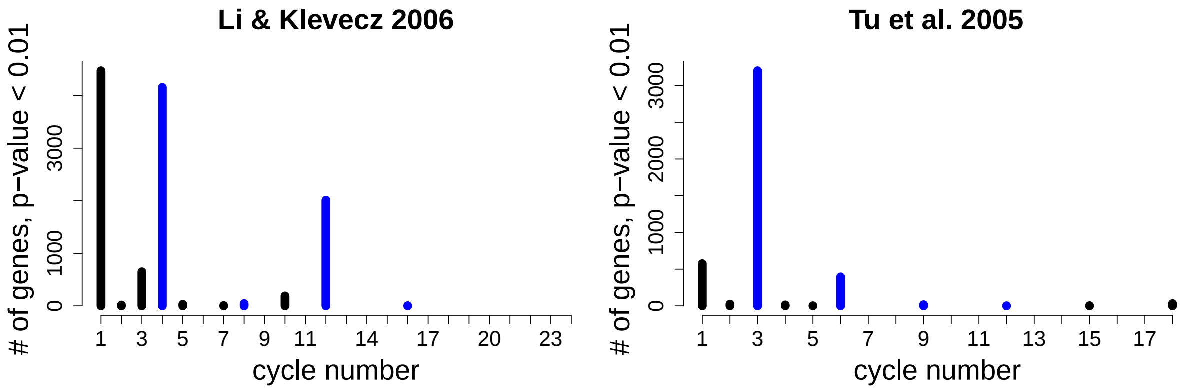

A

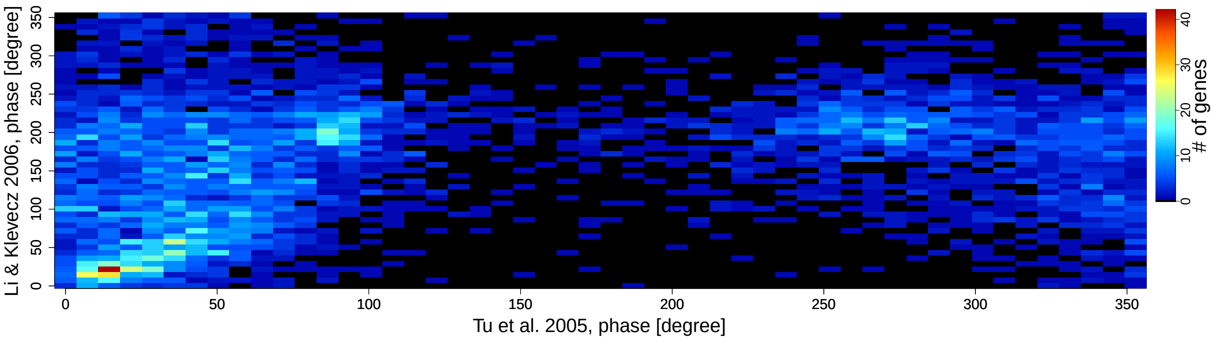

B

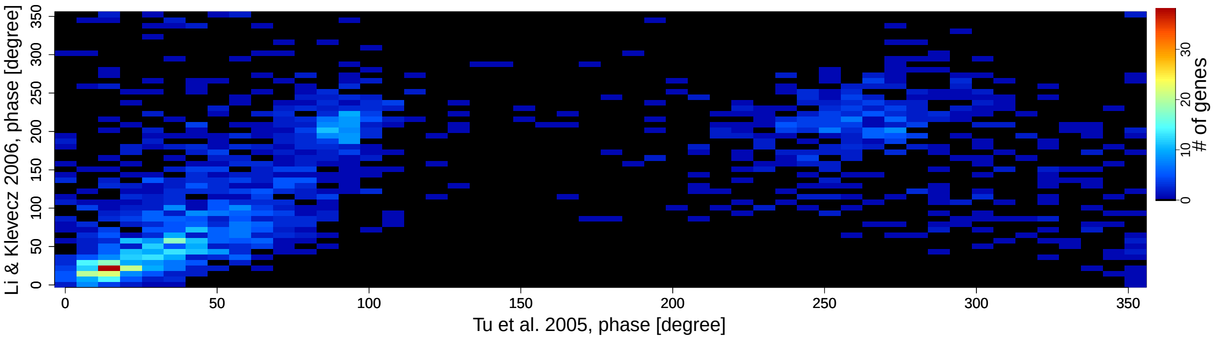

C

Supporting Text Figure ST2. Fourier analysis of gene expression data. ST2A: oscillation p-value spectra; number of genes with a permutation test p-value $p_k < 0.01$ at different cycle numbers k (see Text S1). The cycle numbers k_c, 4 and 3, resp., and their harmonics (integer multiples of k_c are shown in blue). ST2B: phase-phase comparison; 2D histogram (density plot) of the phase angles of the DFT component at k_c of the respective dataset. ST2C: same as ST2B but only for genes with an oscillation p-value $p_{k_c} < 0.001$ in both datasets.

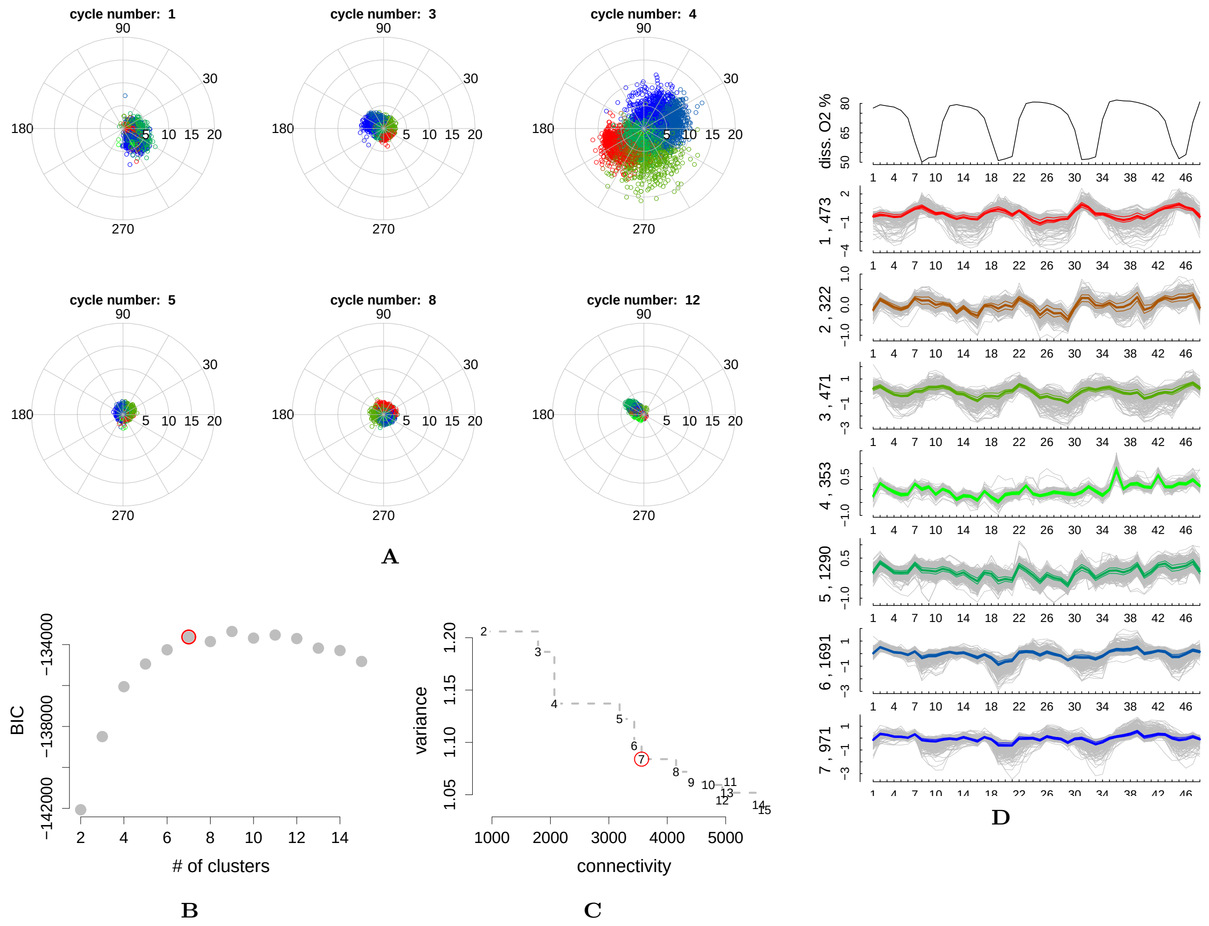

Supporting Text Figure ST3. DFT-based clustering of Li and Klevecz 2006 data. Discrete Fourier Transform (DFT) and clustering of the microarray dataset from [2]. ST3A: polar plots of the DFT components used for clustering, *i. e.*, $k_s = \{1, 3, 4, 5, 8, 12\}$. Colors indicate the selected clustering. ST3B and ST3C, evaluation of clusterings: the clustering selected for further analysis is indicated by a red circle. ST3B: Bayesian Information Criterion (BIC) from the clustering algorithm [25]. ST3C: 2-objective plot of the respective cluster variance and connectivity calculated from the original time series data (as the log-ratio, $\log_2(x/\bar{x})$) after [9]; both, variance and connectivity, should be minimized but behave different with increasing cluster number. ST3D: individual time courses of microarray fluorescence (log-ratio) of the resulting clusters, after sorting by (phase-shifted) circular density peaks of the phase angles ϕ_{k_c} and re-labelling. The y-axis labels give the cluster assignments and the number of genes in each cluster. The thick and thin colored lines are the cluster mean and upper and lower quartiles, respectively, and gray lines are individual transcript time series. The clustering is available in Dataset S1.

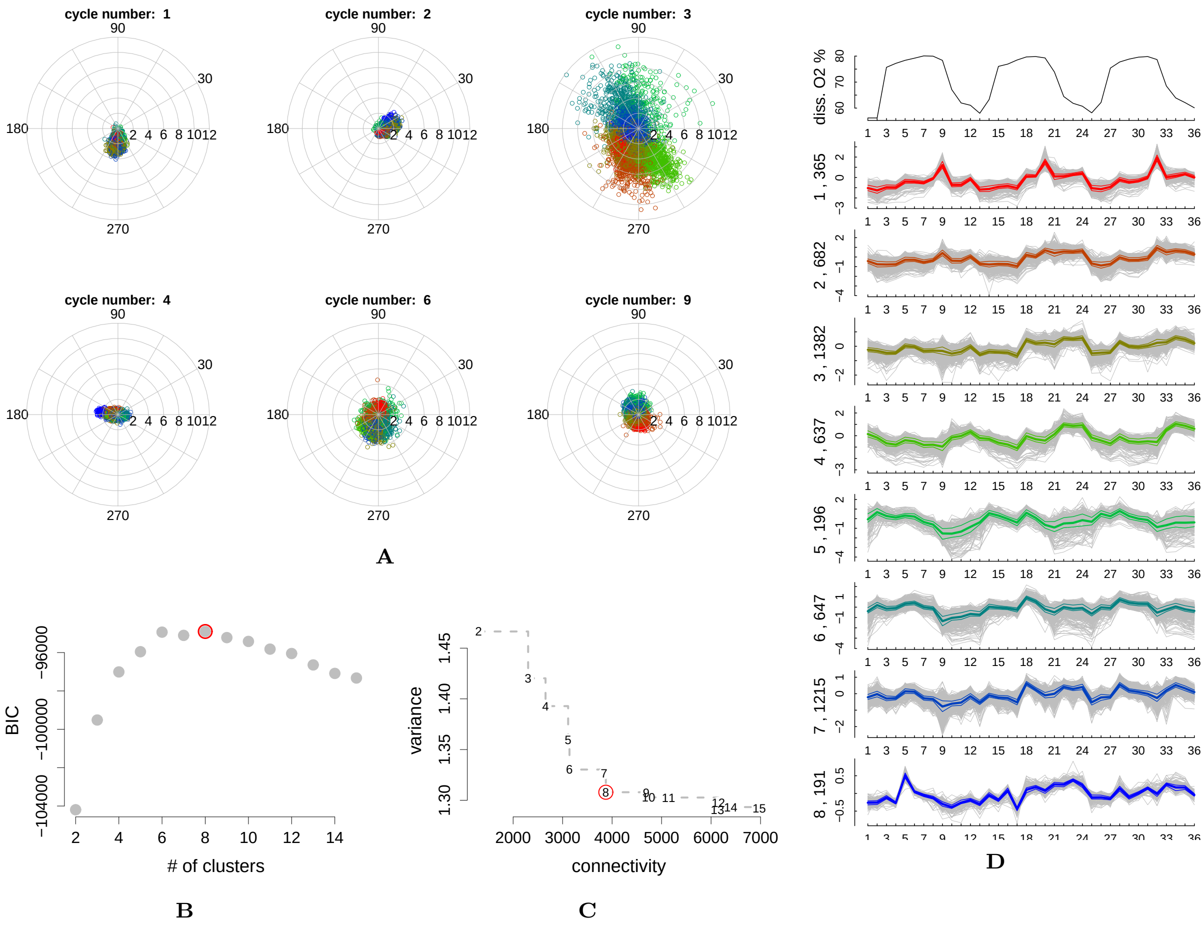

Supporting Text Figure ST4. DFT-based clustering of Tu et al. 2005 data. Discrete Fourier Transform (DFT) and clustering of the microarray dataset from [3]. ST4A: polar plots of the DFT components used for clustering, *i. e.*, $k_s = \{1, 2, 3, 4, 6, 9\}$. Colors indicate the selected clustering. ST4B and ST4C, evaluation of clusterings: the clustering selected for further analysis is indicated by a red circle. ST4B: Bayesian Information Criterion (BIC) from the clustering algorithm [25]. ST4C: 2-objective plot of the respective cluster variance and connectivity calculated from the original time series data (as the log-ratio, $\log_2(x/\bar{x})$) after [9]; both, variance and connectivity, should be minimized but behave different with increasing cluster number. ST4D: individual time courses of microarray fluorescence (log-ratio) of the resulting clusters, after sorting by (phase-shifted) circular density peaks of the phase angles ϕ_{k_c} and re-labelling. The y-axis labels give the cluster assignments and the number of genes in each cluster. The thick and thin colored lines are the cluster mean and upper and lower quartiles, respectively, and gray lines are individual transcript time series. The clustering is available in Dataset S1.

References

1. Klevecz RR, Bolen J, Forrest G, Murray DB (2004) A genomewide oscillation in transcription gates DNA replication and cell cycle. Proc Natl Acad Sci U S A 101: 1200-5.

2. Li CM, Klevecz RR (2006) A rapid genome-scale response of the transcriptional oscillator to perturbation reveals a period-doubling path to phenotypic change. Proc Natl Acad Sci U S A 103: 16254-9.

3. Tu BP, Kudlicki A, Rowicka M, McKnight SL (2005) Logic of the yeast metabolic cycle: temporal compartmentalization of cellular processes. Science 310: 1152-8.

4. Kraft C, Deplazes A, Sohrmann M, Peter M (2008) Mature ribosomes are selectively degraded upon starvation by an autophagy pathway requiring the Ubp3p/Bre5p ubiquitin protease. Nat Cell Biol 10: 602-610.

5. Calza S, Valentini D, Pawitan Y (2008) Normalization of oligonucleotide arrays based on the least-variant set of genes. BMC Bioinformatics 9: 140.

6. Spellman P, Sherlock G, Zhang M, Iyer V, Anders K, et al. (1998) Comprehensive identification of cell cycle-regulated genes of the yeast Saccharomyces cerevisiae by microarray hybridization. Mol Biol Cell 9: 3273-3297.

7. de Lichtenberg U, Jensen L, Fausboll A, Jensen T, Bork P, et al. (2005) Comparison of computational methods for the identification of cell cycle-regulated genes. Bioinformatics 21: 1164-1171.

8. Ptitsyn A, Zvonic S, Gimble J (2006) Permutation test for periodicity in short time series data. BMC Bioinformatics 7 Suppl 2: S10.

9. Handl J, Knowles J, Kell D (2005) Computational cluster validation in post-genomic data analysis. Bioinformatics 21: 3201-3212.

10. Yeung K, Fraley C, Murua A, Raftery A, Ruzzo W (2001) Model-based clustering and data transformations for gene expression data. Bioinformatics 17: 977-987.

11. Qian J, Dolled-Filhart M, Lin J, Yu H, Gerstein M (2001) Beyond synexpression relationships: local clustering of time-shifted and inverted gene expression profiles identifies new, biologically relevant interactions. J Mol Biol 314: 1053-1066.

12. Bar-Joseph Z, Gerber G, Gifford D, Jaakkola T, Simon I (2003) Continuous representations of time-series gene expression data. J Comput Biol 10: 341-356.

13. Peddada S, Lobenhofer E, Li L, Afshari C, Weinberg C, et al. (2003) Gene selection and clustering for time-course and dose-response microarray experiments using order-restricted inference. Bioinformatics 19: 834-841.

14. Moeller-Levet C, Klawonn F, Cho KH, Yin H, Wolkenhauer O (2005) Clustering of unevenly sampled gene expression time-series data. Fuzzy Sets and Systems 152: 49 - 66.

15. Ernst J, Bar-Joseph Z (2006) STEM: a tool for the analysis of short time series gene expression data. BMC Bioinformatics 7: 191.

16. Dejean S, Martin P, Baccini A, Besse P (2007) Clustering time-series gene expression data using smoothing spline derivatives. EURASIP J Bioinform Syst Biol : 70561.

17. Kim J, Kim H (2008) Clustering of change patterns using fourier coefficients. Bioinformatics 24: 184-191.

18. Wang X, Wu M, Li Z, Chan C (2008) Short time-series microarray analysis: methods and challenges. BMC Syst Biol 2: 58.

19. Subhani N, Rueda L, Ngom A, Burden C (2010) Multiple gene expression profile alignment for microarray time-series data clustering. Bioinformatics 26: 2281-2288.

20. Koenig L, Youn E (2011) Hierarchical signature clustering for time series microarray data. Adv Exp Med Biol 696: 57-65.

21. Kim BR, Littell RC, Wu R (2006) Clustering periodic patterns of gene expression based on Fourier approximations. Current Genomics 7: 197-203.

22. Li N, McMurry T, Berg A, Wang Z, Berceli S, et al. (2010) Functional clustering of periodic transcriptional profiles through ARMA(p,q). PLoS One 5: e9894.

23. Fasold M, Stadler P, Binder H (2010) G-stack modulated probe intensities on expression arrays - sequence corrections and signal calibration. BMC Bioinformatics 11: 207.

24. Lo K, Brinkman RR, Gottardo R (2008) Automated gating of flow cytometry data via robust model-based clustering. Cytometry Part A : the journal of the International Society for Analytical Cytology 73: 321–332.

25. Lo K, Hahne F, Brinkman R, Gottardo R (2009) flowClust: a Bioconductor package for automated gating of flow cytometry data. BMC Bioinformatics 10: 145.

26. Pihur V, Datta S, Datta S (2009) RankAggreg, an R package for weighted rank aggregation. BMC Bioinformatics 10: 62.
